# Comparative Efficacy of Different Protein Supplements on Muscle Mass, Strength, and Physical Indices of Sarcopenia among Community-Dwelling, Hospitalized or Institutionalized Older Adults Undergoing Resistance Training: A Network Meta-Analysis of Randomized Controlled Trials

**DOI:** 10.3390/nu16070941

**Published:** 2024-03-25

**Authors:** Chun-De Liao, Shih-Wei Huang, Hung-Chou Chen, Mao-Hua Huang, Tsan-Hon Liou, Che-Li Lin

**Affiliations:** 1 International Ph.D. Program in Gerontology and Long-Term Care, College of Nursing, Taipei Medical University, New Taipei City 110301, Taiwan; 08415@s.tmu.edu.tw; 2 Department of Physical Medicine and Rehabilitation, Shuang Ho Hospital, Taipei Medical University, New Taipei City 235041, Taiwan; 13001@s.tmu.edu.tw (S.-W.H.); 10462@s.tmu.edu.tw (H.-C.C.); peter_liou@s.tmu.edu.tw (T.-H.L.); 3 Department of Physical Medicine and Rehabilitation, School of Medicine, College of Medicine, Taipei Medical University, New Taipei City 110301, Taiwan; 4 Department of Biochemistry, University of Washington, Seattle, WA 98015, USA; huangkevin507@gmail.com; 5 Department of Orthopedic Surgery, Shuang Ho Hospital, Taipei Medical University, New Taipei City 23561, Taiwan; 6 Department of Orthopedics, School of Medicine, College of Medicine, Taipei Medical University, New Taipei City 11031, Taiwan

**Keywords:** sarcopenia, protein supplement, resistance exercise, muscle mass, strength, mobility

## Abstract

Aging-related sarcopenia exerts harmful impacts on muscle mass, strength, and physical mobility. Protein supplementation has been demonstrated to augment efficacy of resistance training (RT) in elderly. This study compared the relative effects of different protein supplements on muscle mass, strength, and mobility outcomes in middle-aged and older individuals undergoing RT. A comprehensive search of online databases was performed to identify randomized controlled trials (RCTs) examining the efficacy of protein supplement plus RT in untrained community-dwelling adults, hospitalized, or institutionalized residents who suffered acute or chronic health conditions. Network meta-analysis (NMA) was performed using a frequentist method for all analyses. Treatment effects for main outcomes were expressed as standard mean difference (SMD) with 95% confidence interval (CI). We used the surface-under-the cumulative-ranking (SUCRA) scores to rank probabilities of effect estimation among all identified treatments. Meta-regression analyses were performed to identify any relevant moderator of the treatment efficacy and results were expressed as β with 95% credible interval (CrI). We finally included 78 RCTs (5272 participants) for analyses. Among the six protein sources identified in this NMA, namely whey, milk, casein, meat, soy, and peanut, whey supplement yielded the most effective treatments augmenting efficacy of RT on muscle mass (SMD = 1.29, 95% CI: 0.96, 1.62; SUCRA = 0.86), handgrip strength (SMD = 1.46, 95% CI: 0.92, 2.00; SUCRA = 0.85), and walking speed (SMD = 0.73, 95% CI: 0.39, 1.07; SUCRA = 0.84). Participant’s health condition, sex, and supplementation dose were significant factors moderating the treatment efficacy on muscle mass (β = 0.74; 95% CrI: 0.22, 1.25), handgrip strength (β = −1.72; 95% CrI: −2.68, −0.77), and leg strength (β = 0.76; 95% CrI: 0.06, 1.47), respectively. Our findings suggest whey protein yields the optimal supplements to counter sarcopenia in older individuals undergoing RT.

## 1. Introduction

Sarcopenia, an age-driven skeletal muscle condition, has been recognized as a muscle disease characterized by progressive attenuation of muscle mass [1,2]. The loss of muscle mass can further impact muscle strength and its function performance which in turn results in high risks of physical difficulty and disability in elderly [3,4,5,6]. Senile primary sarcopenia commonly occurs with advancing age which is accompanied with a number of chronic comorbidities or long-term health conditions such as cardiovascular diseases [7], arthritis [8,9,10,11], diabetes mellitus [12], and stroke [13]; accordingly, sarcopenia has become epidemic in the ageing population. Depending on the various diagnostic criteria, 5–13% of people who aged older than 60 years are identified as having sarcopenia, with an increasing prevalence up to 50% in elderly aged older than 80 years [14,15]. In worldwide, an overall estimate of prevalence of sarcopenia is reported to be around 10% in community-dwelling older individuals, 31–51% in long-term care institutions and 24% in the acute hospital-care population [16,17]. Because of the growing numbers on prevalence and the accompanied adverse effects of sarcopenia [18,19,20,21,22], such disease is responsible for considerable burden in healthcare system [23,24]. Therefore, it is imperative to investigate effective treatment strategies developed to preserve or restore muscle mass as early as possible for elderly people.

The primary sarcopenia is defined based on handgrip strength, lean mass, and mobility due to progressive skeletal muscle wasting and related physical decline with aging. The major underlying mechanism driving the disease development and progression of sarcopenia has been addressed to the blunted muscle protein synthetic response (i.e., myogenesis) and negative muscle net balance in elder people [25,26,27]. Additionally, sarcopenia is commonly recognized as a multi-etiological disease associated with a multiplicity of physical, psychological, and social factors in community-dwelling older adults, among which physical inactivity as well as malnutrition are determinant factors that have great impacts on sarcopenia [28]. Therefore, the most effective managements for sarcopenia rely on the adoption of healthy lifestyle behaviors, including adherence to regular physical activity and high-quality diets [29,30,31,32]. Under such scenario, the optimal treatment strategy of sarcopenia comprises multidisciplinary interventions including exercise and nutrition treatment [33,34,35]. With respect to physical activity intervention, resistance training (RT) has been identified as a successful exercise therapy which have promising effects on counteracting sarcopenia in terms of increased whole-body lean mass and muscular strength in elderly population [36,37,38,39]. Aside from regular physical activity, nutritional interventions using nutrient supplements are targeted to prevent or postpone the onset of such geriatric conditions derived from sarcopenia [32,40,41]. Especially, a high-protein diet or protein supplementation is believed to have surplus benefits in augmenting efficacy of RT for healthy older people [42,43,44,45], as well as those who have high risks of sarcopenia [41,43,44,45,46,47,48,49,50,51]. Sufficient protein intake plays a key role to stimulate maximal rate of muscle protein synthesis leading to anabolism and net new muscle protein accretion during RT [52,53]. Such resultant net of muscle protein is in turn accumulated over the training period to yield muscle mass gain or morphological hypertrophy.

The postprandial muscle protein synthetic response appears to be modulated by the amount, source, and type of protein consumed [54]. Numerous protein nutrients are available for individuals to intake and meet their daily protein needs, including animal-derived (e.g., whey, casein, milk, or beef etc.) and plant-derived (e.g., soy, pea, or rice etc.) dietary protein sources, and most of which have been shown to effectively stimulate post-exercise myofibrillar protein synthesis rates [55,56,57,58]. Due to that rate of digestibility and amount of leucine content are varied among different protein supplements, the muscle protein synthesis rates in response to the ingestion of such protein supplements may differ at rest and following RT. Empirical support for this inference have been observed [59,60,61]. Therefore, in terms of muscle protein synthesis rate, the protein source and type deem an important factor determining the acute treatment efficacy of such intervention of nutrient supplementation along with RT. However, it remains unclear whether the variety type or source of protein supplements obtain different long-term outcome of muscle mass and its function after RT intervention. Identifying relative treatment efficacy among different protein supplements assists the clinical practitioners establishing optimal and efficient intervention strategies.

Previous systemic review and meta-analysis studies have compared the effects of different protein sources during exercise training [44,62,63,64,65,66,67], among which the compared protein supplements included whey [44,62,63,64,65,67], milk [44,63,65,67], casein [44,62,65,67], beef [63,64], soy [62,63,65,67], and cereal protein [63,65]. However, all such protein supplements were not yet fully compared in single meta-analysis study and the most effective treatment option remains unknown. In addition, most of the previous systemic reviews enrolled both young and older adults, only few targeted the elderly population alone [44,65,67]. Based on the increasing number of sarcopenic individual among elderly population, there is an urgent need to identify the most effective and efficient treatment strategy for such population restoring their muscle strength and function. With respect to the above considerations, the purpose of this study was to (1) compare the relative efficacy of different type of protein supplements combined with RT on muscle mass, strength, and physical mobility by using network meta-analysis (NMA); (2) identify if there is any relevant moderator affecting the relative treatment efficacy; and (3) determine the optimal treatment by ranking the probability of each supplement type for community-dwelling or institutionalized older adults undergoing RT.

## 2. Materials and Methods

### 2.1. Study Protocol

This NMA adapted the guidelines of the Preferred Reporting Items for Systematic Reviews and Meta-Analyses (PRISMA) [68] and the extension statement for reporting of systematic reviews incorporating NMA [69,70]. The protocol of the present NMA was registered at PROSPERO (registration number: CRD42023472942). 

### 2.2. Search Strategy

Electronic databases including PubMed, the Cochrane Library Database, EMBASE, the ClinicalKey, the China Knowledge Resource Integrated Database, the Physiotherapy Evidence Database, and Google Scholar were searched from inspection to 31 July 2023 to identify eligible randomized controlled trials (RCTs) investigating the efficacy of protein supplementation (PS), resistance training (RT), or combined treatment for older people. We also performed manual search for RCTs cited in articles retrieved from the databases. There was no limitation for the publication year or language.

The following search terms were based on PICOS (Participants, Intervention, Control, Outcomes, Study design) criteria: (1) Participants’ conditions: (“older adult” OR “elder individual”) AND (“untrained” OR “sedentary” OR “frailty” OR “sarcopenia” OR “nursing home” OR “institutionalization” OR “hospitalization”). (2) Intervention: (“resistance exercise training” OR “strengthening exercise”) AND (“protein supplementation” OR “high-protein diet”); (3) Comparisons: placebo supplementation with or without RT, RT alone, usual medication or regular care (RC) without any PS or RT; (4) Outcomes: lean mass OR strength OR mobility. The details for main outcome measures were described in the following Section 2.4; (5) Study design: a parallel or crossover RCT with two or multiple study arms. The detailed search formulas for each database are presented in Supplementary Table S1.

### 2.3. Criteria for Selecting Studies

Trials were included if they met the following criteria: (1) the study design was an RCT (2) the study enrolled institutionalized residents or community-dwelling elders who aged ≥50 years, were untrained, sedentary, frail, or identified as having sarcopenia. Participants were excluded if they had considerable conditions in hematological, hepatic, or renal functions; (3) the treatment groups received PS in combination with RT. The PS involved different type of protein supplements derived from animal or plant sources; (4) the comparator group received an active treatment including comparative PS alone, placebo supplementation, RT with or without placebo supplementation, or regular care (RC) which was not related to PS or RT; and (5) the study reported main outcome measures defined in the following Section 2.4.

The exclusion criteria were as follows: (1) a vitro or vivo study; (2) a case report or case series study; and (3) a prospective study without randomised allocation.

Study search, screen, and selection for relevant studies were independently performed by two authors (CDL and SWH). Another team members (THL and CLL) were consulted for any disagreement between the authors until consensus reached.

### 2.4. Main Outcomes

The main outcomes in this NMA included measures of muscle mass, muscle strength, and physical mobility, all of which are diagnostic indicators of sarcopenia recommended by the Asian Working Group for Sarcopenia [1] and the European Working Group on Sarcopenia in Older People [2]. The measures assessing skeletal muscle mass and volume included, but were not limited to, whole body lean mass, fat-free mass, appendicular lean mass, muscle thickness, muscle cross-sectional area, and circumference of arms or legs. Regarding muscle strength measures, handgrip strength was extracted as priori [2]. The strength measures for lower extremity were extracted based on the following sequence of preference: Peak torque and maximum voluntary isometric contraction of quadriceps hamstring muscles, and hip abductors. Physical mobility was assessed using walking speed, chair rising ability, timed up-and-go (TUG) task, or the Short Physical Performance Battery (SPPB) score.

### 2.5. Data Extraction

We established a worksheet to extract the following data from each included study: (1) characteristics of study arm according to the intervention nature; (2) characteristics of the participants including age, sex distribution, and body mass index (BMI); (3 characteristics of the PS and RT protocols; (4) follow-up time points; and (5) main outcome measures. The relevant data from included trials were initially extracted by the author (CDL), and another author (SWH) validated and confirmed the extracted data. In cases of inconclusiveness, the author (CLL) was consulted to discuss the disagreements in a consensus meeting. 

Subgroup analysis was performed based on the follow-up time frame which was defined as short-term (≤3 months), medium-term (>3 months and ≤6 months), and long-term (>6 months) for each outcome; when multiple time points were conducted within the same time frame, the longest one was selected for analysis (e.g., if the follow-up time points for muscle mass measurement were 6 and 12 weeks, the data measured at the 3-month time point were used as the short-term results). We also examined the compliance as well as side effects responding to PS and RT interventions among the included studies.

### 2.6. Risks of Bias in Individual Study and across Studies

Two reviewers (CDL and SWH) independently assessed the risk of bias of the included studies using the Cochrane Collaboration’s risk-of-bias tool for quality assessment [71,72]. Any discrepancy between reviewers’ assessments was resolved by a consensus meeting and, if necessary, discussed with a third reviewer (THL) until consensus was reached. The following six domains seven judgement items related to biased estimates of treatment effects were assessed [72]: (1) the selection bias determined by two judgements regarding random sequence generation and allocation concealment; (2) the performance bias judged by blinding of participants; (3) the detection bias associated with blind of outcome assessors; (4) the attrition bias corresponded with incomplete outcome data; (5) the reporting bias raised from selective reporting; and (6) other bias considering other sources of potential bias such as lack of information on sponsors, funding sources, or author conflict of interest disclosures which raise agenda bias. Each item was judged as low-risk, unclear-risk (specific details or description were not reported) or high-risk (not fulfilling the criteria) according to criteria specified by Cochrane. Based on the judged scores of the six domains of bias, an overall bias was identified as low, unclear, or high risk for each study [72].

To assess publication bias for each outcome, a funnel plot was employed to visually inspect any asymmetry among all effect sizes of treatment arms. In addition, we performed the Begg–Mazumdar rank correlation test to identify any significant publication bias across all included RCTs in each outcome [73].

### 2.7. Data Synthesis and Analysis

Treatment effects were expressed as standard mean differences (SMDs) alongside 95% confidence intervals (CIs) of each main outcome across the included RCTs. The mean value of change scores from baseline and its standard deviation (SD) were directly extracted from each study arm of the included RCTs. The missing SD of change score was estimated using the SD of baseline (SD_base_) and posttest (SD_post_) measured data by the following calculation:

$${SD}_{change}=\surd [({SD}_{base}{)}^{2}+({SD}_{post}{)}^{2}-2\times {Corr}_{base–post}\times {SD}_{base}\times {SD}_{post}]$$

Based on the recommended method [74], the within-participant correlation coefficient (Corr_base–post_) represents the mean of the available correlations from the included studies in which the SD change was available. Accordingly, we used the resulted correlation coefficient of 0.9 for muscle mass measures and 0.7 for strength and mobility measures. Wherever the SD was not presented, it was estimated using *p*-values or 95% CIs. If data were reported as the median, range, or interquartile range, we followed the Wan’s methods to estimate the mean value [75]; and accordingly, the SD was estimated by dividing the interquartile range by 1.35 [74]. The magnitude of the SMD was interpreted based on Cohen’s criteria of effect size (*d*) and was categorized in accordance with the criteria [76]: trivial (*d* < 0.10), small (0.10 ≤ *d* < 0.25), medium (0.25 ≤ *d* < 0.40), and large (*d* ≥ 0.40).

A random-effects NMA model was performed using a frequentist method to estimate effects from direct and indirect comparisons among all treatment arms. Heterogeneity as well as global consistency were assessed using the *I*^2^ statistic alongside *τ*^2^ values to estimate variance across the comparisons. The node-splitting method was used to assess consistency between direct and indirect comparisons and results were resented in a forest plot [77]. In addition, a full design-by-treatment interaction model was established to test total inconsistency across all treatment arms [78]. Ranking probabilities of effect estimation among all treatment arms per outcome were performed and expressed as the surface under the cumulative ranking (SUCRA) score [79].

We established the network meta-regression (NMR) models to identify any relevant moderators affecting the relative efficacy among treatment options. For each main outcome, the univariate NMR model was using an individual moderator as a covariate performed accordingly [80]. Substantial moderators were identified on the basis of (1) participant characteristics including age, BMI, female sex distribution of study sample (i.e., percentage proportion of women of total number of participants), world region of study application, and health status (i.e., relatively healthy versus subhealth with acute conditions or chronic diseases [81]); (2) the study methodology, comprising ROB and follow-up time frame; and (3) the intervention protocol, including PS dose, RT intensity (i.e., percentage of one repetition maximum), and treatment time period. The analyses results of NMR were expressed as β with 95% credible interval (CrI).

The participant compliance in response to PS plus RT was measured by all-cause withdrawal rate. In addition, any adverse event reported by the included RCTs was extracted and the corresponded occurrence rate was calculated to evaluate the adverse effects. The analyses results were presented as odds ratios (ORs) alongside 95% CIs.

All NMA and NMR analyses were performed using R statistical software (version 4.2.3, R Foundation for Statistical Computing, Vienna, Austria) [80,82]. For all the analyses, the statistical significance was accepted by a two-tailed *p* value of <0.05.

### 2.8. Certainty of Evidence

The Grading of Recommendation Assessment, Development, and Evaluation (GRADE) system was employed to assess the quality of evidence contributing to NMA estimates for all main outcomes [83]. The GRADE framework evaluates five domains of each treatment arm derived from the included RCTs, namely study limitations, inconsistency, imprecision, incoherence, and publication bias. Based on the ranked score of each domain which was considered to upgrade or downgrade the quality of evidence, the evidence certainty was overall graded as high, moderate, low, and very low. The ranking and grading procedures were independently performed by two individuals (CDL, SWH) and validated by the others (HCC, MHH, THL, CLL).

We assessed the weighted contribution of direct comparisons to the NMA estimates for each outcome using the contributions matrix [84,85]. The downgrading level for the study limitation was based on the amounts of contribution of each direct estimate and corresponding risk of bias to the NMA estimates [86,87].

## 3. Results

### 3.1. Trial Selection Flowchart

**Figure 1:**
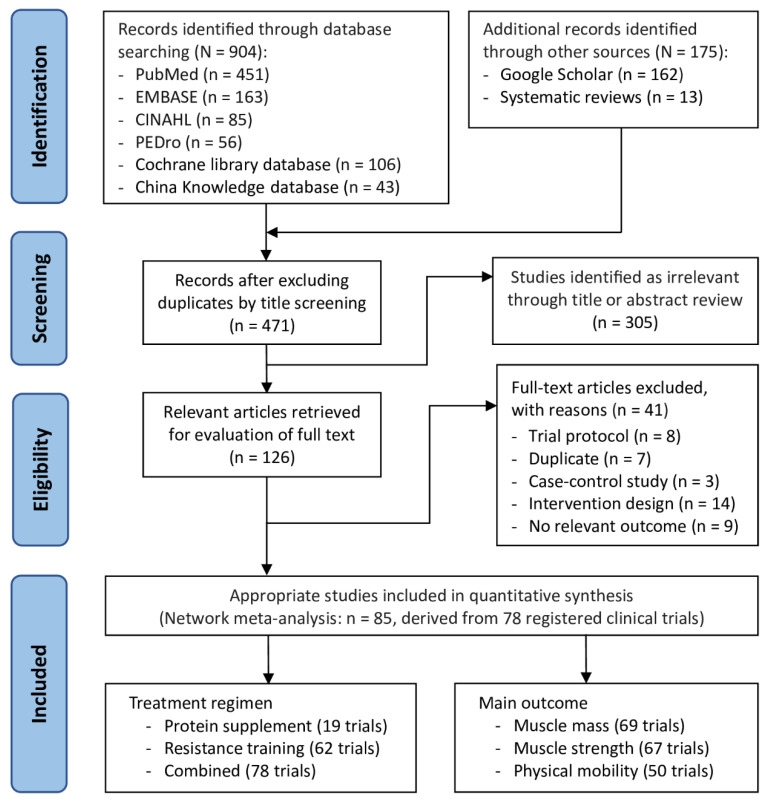
RRISMA flow diagram of study selection. PRISMA, Preferred Reporting Items for Systematic Reviews and Meta-Analyses.

The PRISMA flowchart of the trial selection process was presented in Figure 1. Initially, a total of 1079 articles were identified by electronic searches and manual screen of literature. After excluding duplicates, we examining the retrieved titles of 471 studies and reviewed the abstracts of to assess their eligibility; followed by the full-text assessment of 126 relevant articles. A total of 78 registered clinical RCTs, corresponding with 85 published studies [88,89,90,91,92,93,94,95,96,97,98,99,100,101,102,103,104,105,106,107,108,109,110,111,112,113,114,115,116,117,118,119,120,121,122,123,124,125,126,127,128,129,130,131,132,133,134,135,136,137,138,139,140,141,142,143,144,145,146,147,148,149,150,151,152,153,154,155,156,157,158,159,160,161,162,163,164,165,166,167,168,169,170,171,172], were included in this NMA and all of which were published between 1994 and 2023. The following included studies shared a common trial protocol respectively: 2 studies [89,90], 2 studies [94,110], 2 studies [107,160], 2 studies [120,121], 2 studies [127,128], 2 studies [130,131], and 2 studies [147,148].

### 3.2. Study Characteristics

Table 1 summarizes the participant demographic data and study characteristics of the included RCTs. The details of each trial are presented in the Supplementary Table S2. A total of 5272 participants were recruited among all included RCTs. In overall, the sample had a mean age of 70.2 (range: 47.0–89.2) years and a mean BMI of 25.8 (range: 18.6–33.3) kg/m^2^. Among the 78 included RCTs (Table S2), 39 enrolled both women and men with an average proportion of female participants being 57.6% (range: 12.5–90.2%) whereas 22 had men-specific study design [93,95,96,97,106,109,110,116,121,122,124,127,130,132,137,141,149,156,158,163,167,170], 16 enrolled women only [102,113,123,135,136,143,145,146,147,151,152,155,157,161,165,168], and one did not report the sex distribution of the study sample [103]. 

With respective to participant physical conditions, 33 RCTs out of 78 [92,93,95,96,97,102,106,108,110,112,113,116,118,119,121,123,124,125,130,133,134,136,138,139,141,143,146,151,155,161,162,163,167,168] enrolled community-dwelling untrained middle-aged and older adults who were medically stable and relatively healthy, whereas others recruited individuals who were identified as sedentary or inactive (7 RCTs [91,103,109,126,129,149,153]), overweight or obesity (5 RCTs [140,156,159,164,170]), dynapenia (4 RCTs [88,98,104,169]), frailty (12 RCTs [101,105,111,114,117,132,142,147,150,152,157,160]), and sarcopenia (18 RCTs [89,99,100,115,122,127,135,137,144,145,154,158,165,166,169,171,172]). The study population were enrolled in America (29 RCTs), Asia (18 RCTs), Europe (23 RCTs), and Oceania areas (8 RCTs; Table 1 and Table S2).

Among all participants, 2458 (46.6%) received PS in combination with RT, 415 (7.9%) received PS alone, 1664 (31.6%) received RET alone, and 735 (13.9%) received RC (Table 1). With respect to the follow-up time points for outcome assessment, 57 out of 78 included RCTs conducted a short-term duration of ≤12 weeks; 23 out of 78 RCTs [91,98,99,100,101,102,110,112,123,126,134,136,137,140,143,144,150,151,152,158,160,164,172] employed a medium-term time frame ranging from 14 to 24 weeks, and 4 RCTs [127,130,139,144] assessed a long-term outcome ranging from 9 to 12 months (Table S2).

### 3.3. Protocol of Protein Supplementation

The characteristics for PS protocol are summarized in the Supplementary Table S2. In this NMA, a total of 6 types of protein supplement were identified (Table 1), namely whey protein (WP, 43 RCTs); milk protein (MP, 17 RCTs); soy protein (SP, 14 RCTs); casein (4 RCTs); beef meat (5 RCTs); and peanut (1 RCT). Regarding the PS protocol, 25 out of 78 included RCTs prescribed supplementation immediately after RT on the training days with a mean dosage of 26 (range 10–60) g/day; and the other 53 employed daily supplementation by a mean intake amount of 30 (range 10–80) g during an RT intervention (Table S2).

### 3.4. Protocol of Resistance Training 

Table S2 summaries the characteristics of RT protocols. In overall, the training load was moderate to high with a median (range) intensity of 75% (30–85%) of one-repetition maximum or 7 (5–9) of 10-point rating of perceived exertion. Regarding the training duration, 54 out of 78 included RCTs conducted a short intervention of 12 weeks or less, corresponding with 6–84 sessions; 21 RCTs employed a medium intervention of 14 weeks to 6 months, corresponding with 6–84 sessions; and 3 RCTs [127,130,139] had a long intervention up to 12 months.

### 3.5. Risks of Bias in Individual Study and Across Studies

**Figure 2:**
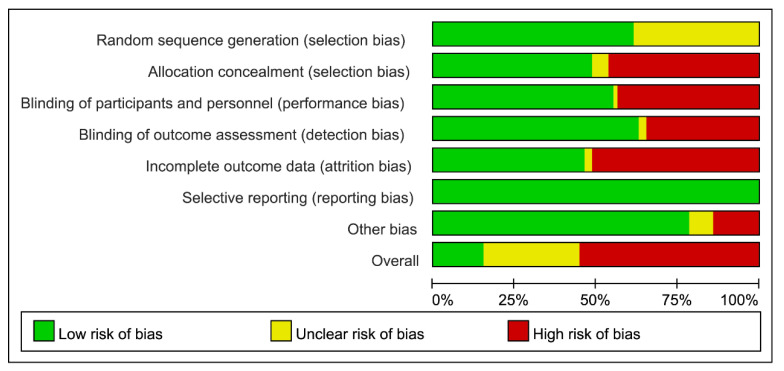
Risk of bias graph crossing trials.

Figure 2 shows a summary of the risk-of-bias analysis for each domain and an overall rank crossing included RCTs. The details of each ranked risk-of-bias item per trial are presented in the Supplementary Figure S1. Using the Cochrane risk-of-bias tool, over half of the included RCTs (43 out of 78, 55.1%) were ranked as having an overall high risk of bias whereas it determined 12 RCTs (15.4%) had low risk of bias and 23 RCTs (29.5%) had some concerns of bias (Figure S1). In total, the common domain of biases which obviously contributed to high risk of bias across all RCTs included at least one of the follows: selection, performance, detection, and attrition biases (Figure 2).

Risk of bias analysis crossing trials showed that 41 RCTs (52.6%) had a substantial unclear or high risk of selection bias raising from insufficient information regarding procedures of randomization or group assignment. Thirty-four RCTs out of 78 (43.6%) were considered having high risks of performance bias for blinding the participants and 27 RCTs (34.6%) were judged having high risks of detection bias because of the unblinded assessors. In addition, 40 RCTs (51.3%) which did not perform intention-to-treat analyses were scored as high risks of attrition bias; and low risk of reporting bias was ranked among all the included trials. Regarding other bias, we assumed that use of PS products supported by any funds or grants may arise potential agenda bias and therefore information of funding sources and conflict-of-interest declaration were assessed. There was potentially high risk of agenda bias in 11 RCTs (14.1%) which reported funding sources without any conflict-of-interest disclosures whereas 6 RCTs (7.7%) which did not report any information regarding funding sources and conflict of interest were considered unclear risk of agenda bias (Figure 2).

### 3.6. Treatment Efficacy for Muscle Mass

**Figure 3:**
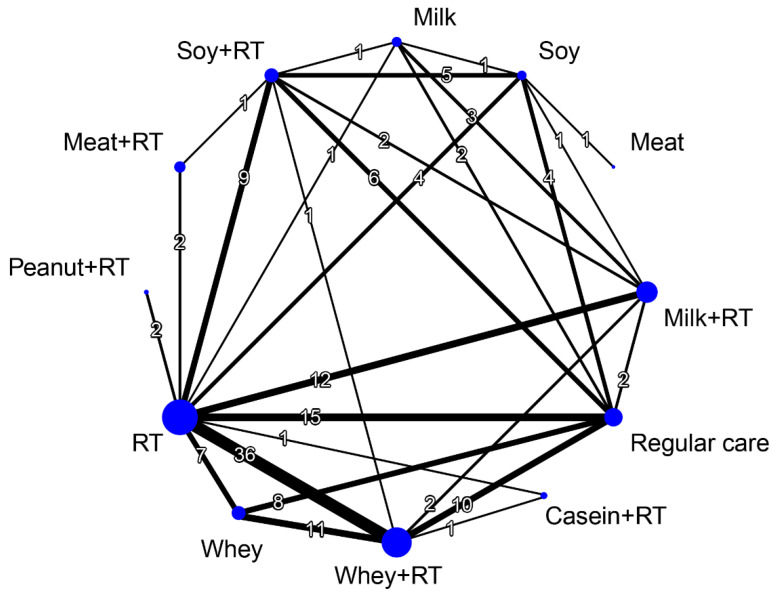
Network diagram illustrating the interconnections among different treatment arms for muscle mass. Each node represents a treatment arm, and the size of the node is proportional to the number of participants assigned to the specific treatment. The thickness of each line is proportional to the number of direct comparisons between arms denoted on the line. RT, resistance training.

Figure 3 presents the network connections among all treatment arms for muscle mass outcome. A total of 11 treatment options were identified in this NMA, included 6 combined treatments namely WP plus RT (WP + RT), MP plus RT (MP + RT), casein plus RT (Casein + RT), meat plus RT (Meat + RT), SP plus RT (SP + RT), peanut plus RT (Peanut + RT), 5 monotherapies namely WP, MP, SP, meat, and RT.

#### 3.6.1. Conventional Pairwise Meta-Analysis

Muscle mass outcome were reported by 73 RCTs along with 12 treatment options and 151 pairwise comparisons in NMA (Figure 3). Direct comparisons of pairwise meta-analyses indicated that WP + RT (SMD = 1.36, 95% CI: 0.90–1.83), MP + RT (SMD = 1.31, 95% CI: 0.41–2.22), SP + RT (SMD = 1.06, 95% CI: 0.49–1.63) and RT (SMD = 0.79, 95% CI: 0.42–1.15) were more efficacious than RC for increasing muscle mass (Supplementary Table S3). In addition, the combined treatments WP + RT (SMD = 0.95, 95% CI: 0.52–1.37) and SP + RT (SMD = 1.21, 95% CI: 0.58–1.85) achieved favor effects on muscle mass gain compared with its monotherapy of WP and SP, respectively. While comparing with RT alone, similar results were observed in WP + RT (SMD = 0.55, 95% CI: 0.17–0.93) and WP + RT (SMD = 0.56, 95% CI: 0.33–0.79).

#### 3.6.2. Network Meta-Analysis

**Figure 4:**
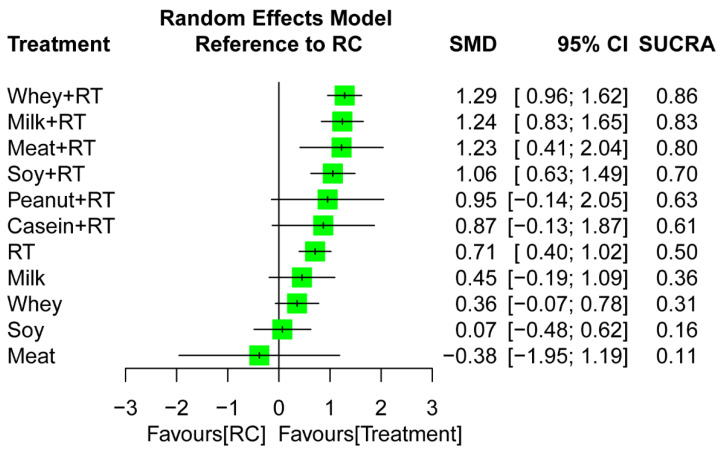
Relative efficacy of treatment regimens on muscle mass gain during an overall follow-up duration. Each point estimate (square) presents the network combined effect (SMD) on the outcome measure relative to RC, with the 95% CI (horizontal line). SMD, standardized mean difference; CI, confidence interval; SUCRA, surface under the cumulative ranking curve; RT, resistance training; RC, regular care.

Considering RC as reference, the combined treatments namely WP + RT (SMD = 1.29), MP + RT (SMD = 1.24), Meat + RT (SMD = 1.23), and SP + RT (SMD = 1.06) achieved favorable outcomes for muscle mass increase regardless of follow-up time (Figure 4). Among all treatment arms, WP + RT was ranked as the most effective (SUCRA = 0.86) for muscle mass gain—followed by MP + RT (SUCRA = 0.83), and Meat + RT (SUCRA = 0.80) (Figure 4). The NMA results were based on significant global heterogeneity across treatment arms (*τ*^2^ = 0.36, *I*^2^ = 73.4%, *p* < 0.0001). Q statistic results assessing total consistency between designs in NMA showed insignificant under the assumption of a full design-by-treatment random-effects model (*τ*^2^ = 0.35, *p* > 0.05). According to the node-splitting analyses results, there was no inconsistencies between direct and indirect effects in NMA for muscle mass gain (Figure S2).

#### 3.6.3. Subgroup Analysis Based on Follow-Up Time

The combined treatment WP + RT yielded the most effective treatment for muscle mass (SMD = 1.32, SUCRA = 0.83) over the short-term duration, whereas MP + RT was ranked the highest probabilities of successful treatment over the medium-term (SMD = 1.48, SUCRA = 0.93) and long-term (SMD = 1.14, SUCRA = 0.83) durations (Figure S3).

### 3.7. Treatment Efficacy for Muscle Strength

**Figure 5:**
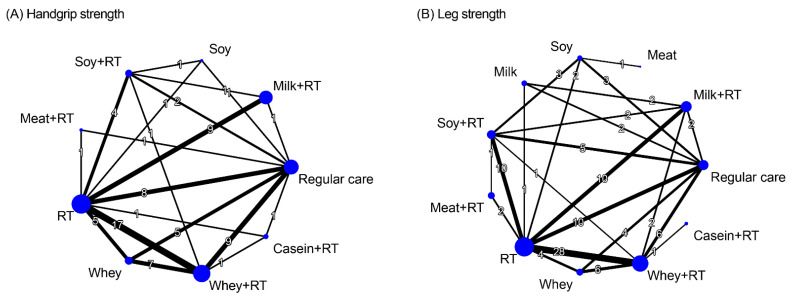
Network diagram illustrating the interconnections among different treatment arms for muscle strength. Each node represents a treatment arm, and the size of the node is proportional to the number of participants assigned to the specific treatment. The thickness of each line is proportional to the number of direct comparisons between arms denoted on the line. RT, resistance training.

Muscle strength outcomes were assessed in terms of handgrip strength and leg strength. A total of 37 RCTs reported treatment outcome of handgrip strength along with 9 treatment regiments and 78 pairwise comparisons in NMA (Figure 5A). The treatment effects on leg strength were investigated by 56 RCTs along with 11 treatment regiments and 104 pairwise comparisons in NMA (Figure 5B).

#### 3.7.1. Conventional Pairwise Meta-Analysis

Results of direct comparisons indicated that PS plus RT achieved favorable effects on handgrip strength gain compared to RC (Supplementary Table S4), particularly the protein types of whey (SMD = 1.53, 95% CI: 0.88–2.19), casein (SMD = 2.98, 95% CI: 0.99–4.97), and soy (SMD = 1.51, 95% CI: 0.11–2.90). In addition, WP + RT yielded greater changes in handgrip strength than WP alone did (SMD = 0.86, 95% CI: 0.11–1.61); similar results were presented for the comparison between SP + RT and SP alone (SMD = 2.46, 95% CI: 0.44–4.48; Table S4).

The pairwise meta-analysis results for leg strength showed that WP + RT (SMD = 1.30, 95% CI: 0.73–1.87) and SP + RT (SMD = 1.73, 95% CI: 1.14–2.32) achieved favorable effects on leg strength gain compared to RC (Supplementary Table S5). Additionally, the combined treatments WP + RT yielded greater changes in leg strength than the monotherapy WP (SMD = 1.30, 95% CI: 0.76–1.84) or RT (SMD = 0.45, 95% CI: 0.20–0.69) did; similar results were observed in SP + RT when it compared with SP alone (SMD = 1.92, 95% CI: 1.16–2.68; Table S5).

#### 3.7.2. Network Meta-Analysis

**Figure 6:**
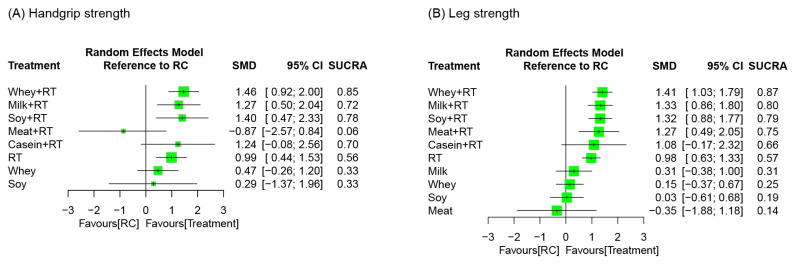
Relative efficacy of treatment regimens on muscle strength during an overall follow-up duration. Each point estimate (square) presents the network combined effect (SMD) on the outcome measure relative to RC, with the 95% CI (horizontal line). SMD, standardized mean difference; CI, confidence interval; SUCRA, surface under the cumulative ranking curve; RT, resistance training; RC, regular care.

There were significant effects in favor of PS plus RT for handgrip strength compared to RC, particularly the protein sources derived from whey (SMD = 1.46), soy (SMD = 1.41), and milk (SMD = 1.27), during an overall follow-up duration (Figure 6A). Among all treatment options, WP + RT yielded the highest probabilities of treatment effects (SUCRA = 0.84)—followed by SP + RT (SUCRA = 0.78) and MP + RT (SUCRA = 0.72)—during the overall follow-up duration (Figure 6A). There was significant global heterogeneity across all treatment arms involved in NMA model (*τ*^2^ = 0.88, *I*^2^ = 91.7%, *p* < 0.0001). Q statistic results assessing total consistency between designs in NMA showed insignificant under the assumption of a full design-by-treatment random-effects model (*τ*^2^ = 0.88, *p* > 0.05). According to the node-splitting analyses results, there was no inconsistencies between direct and indirect effects in NMA for handgrip strength gain (Figure S4). 

With respect to treatment outcome of muscle strength in the lower extremity (Figure 6B), majority of the PS regimens augmented increases in leg strength during RT, including whey, milk, soy, and meat (SMD = 1.27–1.41). In addition, WP + RT yielded the most effective (SUCRA = 0.87) for muscle strength in the legs—followed by MP + RT (SUCRA = 0.80) and SP + RT (SUCRA = 0.79; Figure 6B). Significant global heterogeneity was observed (*τ*^2^ = 0.30, *I*^2^ = 75.6%, *p* < 0.0001). Q statistic results assessing total consistency between designs in NMA showed insignificant under the assumption of a full design-by-treatment random-effects model (*τ*^2^ = 0.88, *p* > 0.05). According to the node-splitting analyses results, there was no inconsistencies between direct and indirect effects in NMA for leg strength (Figure S5).

#### 3.7.3. Subgroup Analysis Based on Follow-Up Time

In handgrip strength, WP + RT yielded the highest medium-term (SMD = 1.74, SUCRA = 0.83) and long-term (SMD = 1.69, SUCRA = 0.99) treatment efficacy, respectively (Figure S6). Within the short-term time frame, SP + RT was ranked the most effective among whole treatment regimens in NMA (SMD = 1.42, SUCRA = 0.91).

For leg strength gain, the combined regimen SP + RT yielded the highest short-term efficacy (SMD = 1.52, SUCRA = 0.93), whereas MP + RT was ranked the most effective among all treatment regimens over the medium-term (SMD = 1.11, SUCRA = 0.95) and long-term (SMD = 1.21, SUCRA = 0.95) time frames, respectively (Figure S7).

### 3.8. Effectiveness of Treatment for Physical Mobility

**Figure 7:**
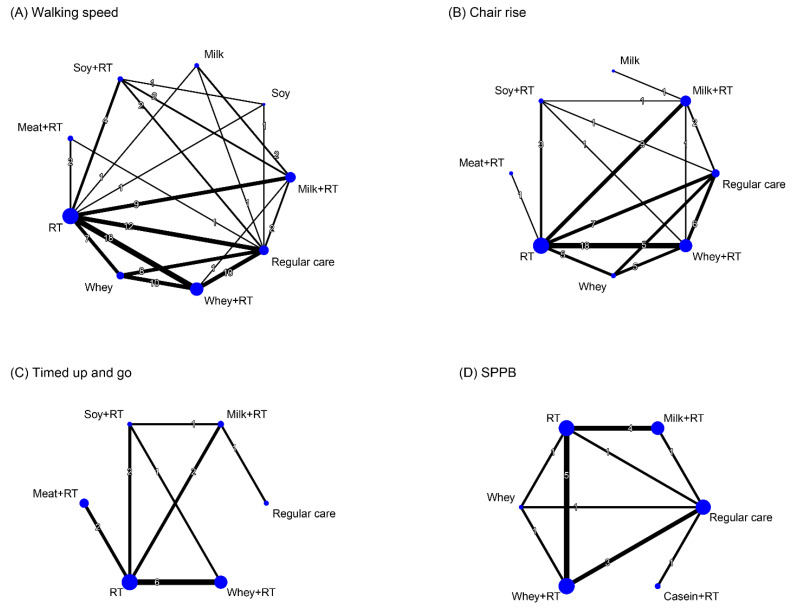
Network diagram illustrating the interconnections among different treatment arms for physical mobility. Each node represents a treatment arm, and the size of the node is proportional to the number of participants assigned to the specific treatment. The thickness of each line is proportional to the number of direct comparisons between arms denoted on the line. RT, resistance training; SPPB, Short Physical Performance Battery.

Treatment efficacy on physical mobility were assessed by walking speed (35 RCTs), chair rise (31 RCTs), TUG (8 RCTs), and global function in terms of SPPB score (13 RCTs). In walking speed, a total of 9 treatment options were identified along with 91 pairwise comparisons in NMA (Figure 7A). In addition, a total of 8 treatment options (62 pairwise comparisons) and 6 treatment options (12 pairwise comparisons) were identified in NMA for chair rise (Figure 7B) and TUG (Figure 7C), respectively. For SPPB, 6 treatment options were identified along with 18 pairwise comparisons in NMA (Figure 7D).

#### 3.8.1. Conventional Pairwise Meta-Analysis

Direct pairwise comparisons indicated that WP + RT achieved favorable effects on walking-speed recovery (SMD = 0.84, 95% CI: 0.43–1.25; Supplementary Table S6), chair rise (SMD = 0.85, 95% CI: 0.46–1.25; Supplementary Table S7), and SPPB score increase (SMD = 1.79, 95% CI: 1.09–2.48; Supplementary Table S8) compared to RC, respectively. The monotherapy WP (SMD = 0.76, 95% CI: 0.30–1.22; Table S6) achieved favorable effects on walking-speed recovery compared to RC, as well as RT did in walking speed (SMD = 0.59, 95% CI: 0.22–0.95; Table S6) and chair rise (SMD = 0.82, 95% CI: 0.53–1.11; Table S7). In TUG, direct pairwise comparison results showed no significant effects among all treatment options with reference to RC (Table S9).

#### 3.8.2. Network Meta-Analysis

**Figure 8:**
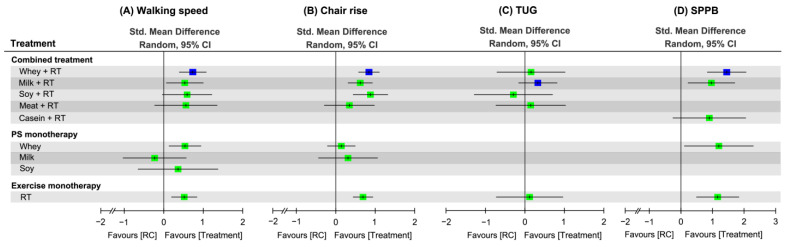
Relative efficacy among treatment regimens on changes in physical mobility scores during an overall study time. Each point estimate (square) presents the network combined effect on the specific outcome measure, with the 95% CI (horizontal line). The positive results indicate favorable efficacy of the treatment regimen compared to RC. The blue-colored point represents the highest probability of effects among all treatment regimens. Std, standardized; CI, confidence interval; RT, resistance training; SPPB, Short Physical Performance Battery; RC, regular care.

For walking speed recovery during an overall time (Figure 8A and Figure S8), the composed regimens WP + RT (SMD = 0.73) and MP + RT (SMD = 0.53) achieved favorable effects compared to RC. Among all treatment options, WP + RT was ranked the most effective treatment to increase walking speed (SUCRA = 0.84; Figure S8). Significant global heterogeneity was observed (*τ*^2^ = 0.28, *I*^2^ = 58.9%, *p* < 0.0001). The full design-by-treatment random-effects model determined that the overall inconsistency between designs in NMA was significant (*τ*^2^ = 0.29, *p* < 0.0001). According to the node-splitting analyses results, it showed no inconsistencies between the direct and indirect comparisons in NMA (Figure S9).

For chair-rise performance at an overall time frame (Figure 8B and Figure S10), the NMA results showed favorable effects responding to WP + RT (SMD = 0.84), as well as SP + RT (SMD = 0.44) and MP + RT (SMD = 0.31), compared to RC. Of all treatment regimens, WP + RT was ranked the optimal treatment to increase chair-rise ability (SUCRA = 0.88; Figure S10). Significant global heterogeneity of the NMA model was identified (*τ*^2^ = 0.12, *I*^2^ = 57.0%, *p* < 0.0001). Using a full design-by-treatment random-effects model, it determined an insignificant overall between-design inconsistency of the NMA (*τ*^2^ = 0.16, *p* > 0.05). The node-splitting analyses did not indicate any inconsistency between direct and indirect effects in NMA (Figure S11).

Regarding TUG task, the NMA results showed no favorable effects responding to any of treatment arms (Figure 8C and Figure S12). Among all treatment arms, MP + RT was ranked as the most effective treatment to save time in completing the TUG task (SUCRA = 0.79; Figure S12). No evidence of the global heterogeneity was identified (*τ*^2^ < 0.0001, *I*^2^ = 0%, *p* > 0.05). The overall between-design inconsistency of the NMA showed insignificant (*τ*^2^ = 0.005, *p* > 0.05). Based on the node-splitting analyses results, there was no inconsistencies between direct and indirect evidence (Figure S13).

With respect to treatment outcome for global function, in terms of SPPB score, the NMA results showed that WP (SMD = 1.45), as well as MP (SMD = 0.97), in combination with RT obtained favorable effects on SPPB score relative to RC, regardless of study time frame (Figure 8D and Supplementary Figure S14). Of all treatment regimens, WP + RT was ranked the optimal treatment to increase SPPB score (SUCRA = 0.87; Figure S14). There was significant global heterogeneity of the NMA model for SPPB (*τ*^2^ = 0.27, *I*^2^ = 75.2%, *p* < 0.0001). There was significant overall between-design inconsistency (*τ*^2^ = 0, *p* < 0.0001). The node-splitting analyses did not indicate any inconsistency between direct and indirect comparisons (Figure S15).

#### 3.8.3. Subgroup Analysis Based on Follow-Up Time

During the short-term time, WP + RT yielded the optimal treatment option to increase performances in walking speed (SMD = 0.74, SUCRA = 0.83; Figure S8) and chair rise (SMD = 0.98, SUCRA = 0.85; Figure S10), as well as MP + RT did for saving timed-up-and-go time (SMD = 0.33, SUCRA = 0.76; Figure S12) and increasing total score of SPPB (SMD = 0.45, SUCRA = 0.71; Figure S14).

Within the medium-term follow-up time frame, WP + RT was ranked the most effective treatment option for walking-speed recovery (SMD = 0.25, SUCRA = 0.78; Figure S8) and to increase total score of SPPB (SMD = 2.19, SUCRA = 0.88; Figure S14), as well as SP + RT and MP + RT did for increasing chair-rise ability (SMD = 0.87, SUCRA = 0.88; Figure S10) and saving TUG time (SMD = 0.33, SUCRA = 0.76; Figure S12), respectively.

Regarding the long-term treatment outcome, WP + RT was ranked the most effective treatment option for walking-speed recovery (SMD = 0.58, SUCRA = 0.98; Figure S8). None of the included RCTs conducted long-term follow up for chair rise, TUG, and SPPB outcome.

### 3.9. Network Meta-Regression Anlyses

Effects of substantial moderators for each main outcome are presented in the Supplementary Table S10. Health status had effects on relative treatment efficacy among treatment arms. Participants who suffered acute (hospitalized) or chronic (obese, sarcopenic, frail, and mobility limited) conditions may achieved greater effects on muscle-mass changes (β = 0.74; 95% CrI: 0.22, 1.25) compared with those who were relatively healthy. In addition, there was potential gender effect on relative treatment efficacy for muscle strength and physical function. A greater proportion of women in the study sample predicted minor effects on handgrip strength (β = −1.72; 95% CrI: −2.68, −0.77) and SPPB (β = −1.61; 95% CrI: −2.47, −0.79). Other potential moderators of relative treatment efficacy were identified, including amounts of PS (β = 0.76; 95% CrI: 0.06, 1.47) and BMI (β = −1.85; 95% CrI: −2.89, −0.81) for leg strength and SPPB, respectively. In addition, a longer intervention period (β = 1.41; 95% CrI: 0.78, 2.05), as well as follow-up time (β = 1.41; 95% CrI: 0.77, 2.03), was associated with greater increases in SPPB score. Furthermore, area of study population had significant influences in strength gains of handgrip (β = −1.04; 95% CrI: −2.00, −0.07) and leg extensors (β = −0.86; 95% CrI: −1.70, −0.05). No moderator was identified to have an influence on the treatment efficacy regarding mobility tasks of walking speed, chair rise, and TUG.

### 3.10. Compliance and Side Effects

**Figure 9:**
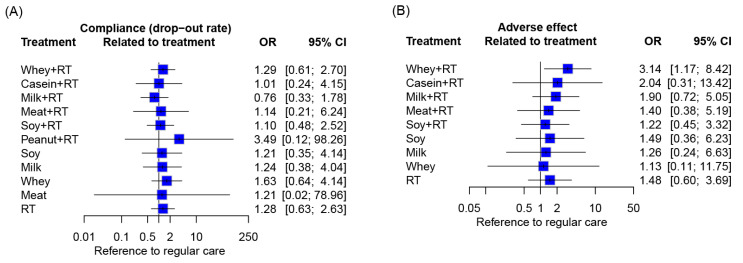
Participant compliance and side effects of treatment regimens. Data concerning treatment-related (**A**) withdrawals and (**B**) adverse events were pooled using inverse variance weighting methods. Each point estimate (square) presents the network combined effect (OR) of the indicated treatment arm relative to RC, with the 95% CI (horizontal line). OR, odds ratio; CI, confidence interval; RT, resistance training.

As a whole, an all-cause attrition rate of 0–78.6% was reported among the included RCTs, of which 0% to 45.2% were dropout due to treatment noncompliance (Table S11). Eleven RCTs reported withdraw of participants due to noncompliance of PS by an attrition rate of 2.0–32.1% [89,91,103,113,119,126,136,143,153,159,169]. Noncompliance of RT were reported by 17 RCTs with an attrition rate of 4.5–31.3% [91,101,103,111,114,121,124,125,126,133,138,140,151,159,164,169,170]. There was no difference in compliance across all the treatment regimens with reference to RC (Figure 9A).

There was no serious adverse event occurred after PS plus RT or its monotherapy. In total, 12 [89,97,98,108,111,117,125,126,136,140,153,159] and 15 [97,98,102,111,112,116,117,121,124,126,130,140,159,164,166] out of the 78 included RCTs reported mild adverse events in response to PS and RT, respectively. Majority of the reported adverse events were RT-induced joint pain and muscle soreness (Table S11) and gastrointestinal symptoms such as diarrhea, bloating, and constipation after PS. Compared with RC, there was no significant adverse effects among all treatment regimens excepting WP + RT (Figure 9B).

### 3.11. Publication Bias

Publication biases among the included RCTs for all outcome domains are presented in the Supplementary Figure S16. The funnel plot indicated no substantial asymmetry of publication bias across the included RCTs. Begg–Mazumdar test results for muscle mass (*p* < 0.01; Figure S16A) and leg muscle strength (*p* < 0.01; Figure S16C indicated obvious reporting bias among the RCTs included in the NMA. No significant bias was observed in other main outcomes.

### 3.12. Certainty of the Evidence 

Table 2 summarizes the GRADE certainty rating for all main outcomes and the details of judgments in each domain of GRADE framework are presented in Supplementary Tables S12–S18. In overall, certainty of the evidence ranged from low to high among combined treatments for muscle mass, strength, and mobility outcomes, particularly WP + RT, SP + RT, MP + RT, and Meat + RT (Table 2). The most common judgements downgrading the certainty of evidence were associated with major concerns about study limitation, imprecision, and publication bias.

## 4. Discussion

The main purpose of this study was to identify the comparative efficacy of different protein sources combined with RT for muscle mass, strength, and physical mobility outcomes in middle-aged and older adults. The main results showed that (1) the combined treatment of protein supplement and RT (particularly WP + RT, SP + RT, MP + RT, and Meat + RT) exerted favorable effects on muscle mass and strength gain relative to RC, whereas WP + RT and MP + RT achieved promising effects on physical mobilities including walking speed, chair rise, and SPPB; (2) based on the cumulative ranking results, whey protein supplements combined with RT yielded the most effective treatment to increase muscle mass, strength, and physical mobility, respectively; (3) a number of potential moderators of relative treatment efficacy were identified, including BMI, gender, healthy status, study area, supplement dosage, and treatment time. In addition, the composite PS plus RT exhibited high compliance despite of higher occurrence rate of nonserious adverse events among PS regimens, particularly WP + RT.

A number of systematic reviews and NMAs have compared the effects among different protein sources of supplementation during exercise training in young and older people [44,62,63,64,65,66,67], and results have generally indicated no significant difference in lean mass or strength gains between WP versus SP [62,63,66], WP versus MP [44], WP versus beef [64], and SP versus other mixed protein sources [63] during an exercise intervention. However, more other protein sources, such as casein and peanut, have yet to be comprehensively compared with conventional protein supplements. In the present study which targeted PS during an RT intervention in middle-aged and older populations, six protein sources (i.e., whey, soy, milk, meat, casein, and peanut) were identified and fully compared in an NMA. Our analyzed results are generally in agreement with those of the previous systematic reviews. We further rank the probabilities of successful treatment among multiple regimens of PS, RT, and its combinations, and the results showed that WP + RT yielded the most effective treatment to increase muscle mass and strength, and to enhance mobility performance. Additionally, the present results indicate that combined treatments incorporating PS and RT can yield extra benefits compared with its monotherapies in adults who aged ≥ 45 years.

In the present study, the ranked results indicated that WP yielded the most effective supplement to augment RT-induced muscle mass gain, followed by MP, meat, SP, and casein (Figure 4). Our findings were supported by previous studies. First, PS monotherapy by WP supplements achieves greater changes in postprandial muscle protein synthesis rate compared with other protein sources, especially in older individuals [67,173], and such favorable effects become more significant for WP + RT [67]. Reasonably, it is expected that the superior effects of WP supplementation on postprandial muscle protein synthesis may lead to favorable long-term changes in RT-induced skeletal muscle adaptation. Secondly, the postprandial muscle protein synthetic response to the ingestion of different protein sources have been compared under RT intervention [61,174,175,176]. The previous results revealed that WP supplements stimulate higher postprandial muscle protein synthesis rates than SP [175] and Casein [61,175] do. In addition, the ingestion of SP after RT stimulates muscle protein synthesis to a greater extent when compared with casein [175], as well as the MP ingestion does while comparing with beef protein [174] and SP supplements [176], respectively. Accounting these previous results together, it suggests that WP exhibits similar effects with MP and is superior to MP, meat, SP, and casein with regard to stimulate muscle protein synthesis, which further supports the ranked results among multiple protein sources identified in this NMA regarding treatment efficacy on muscle mass gain.

Based on the NMA results in the current study, the protein source WP was ranked as the optimal regimen for muscle mass gain during RT intervention and such superiority in treatment efficacy was simultaneously observed in strength and physical mobility. Our findings were in agreement with previous meta-analyses which have concluded that PS synergistically increases muscle mass along with strength gain and physical mobility restoration in older people undergoing exercise training enhancement [177,178,179,180]. Given the facts that muscle mass changes in response to PS significantly contribute to additional increases in strength and walking capability during exercise training [181] and that increased leg strength is associated with faster habitual walking speed following PS plus RT [88], especially in those at high risk of sarcopenia and frailty, the superior effects of WP + RT on muscle mass may parallelly give rise to the superiority of effects on strength and mobility while comparing with other protein sources in this NMA.

In this NMA, a number of substantial moderators were identified to have significant effects on relative efficacy among treatment regimens. First, greater treatment effects on muscle mass were likely to be observed in the participants with acute conditions (hospitalized) or chronic diseases (obese, sarcopenic, frail, and mobility limited) compared with their relatively healthy peers (Table S10). Such findings are in line with the previous systematic review studies indicating that individuals with sarcopenia or frailty risks tend to obtain greater lean mass gains after PS plus exercise training than their healthy peers do [182,183]. Comparing with healthy older, the individuals who suffered frailty, sarcopenia, or other comorbidities have experienced minor habitual intake of protein and lower physical activity level [184,185,186,187], which impacts myofibrillar protein synthetic rates following PS and RT [52,53,187]. However, the ingestion of additional protein during RT may contribute relatively more responses of myofibrillar protein synthesis in those with aging-related conditions than in normal control [188]. Under such scenario, older people with acute or chronic conditions are likely to benefit more in lean mass gains from PS plus RT compared with their healthy peers who achieve minor changes in muscle mass following PS plus RT [189]. Secondly, increases in proportion of women of the study sample may result in decreased changes in handgrip strength and SPPB following PS plus RT; such findings indicated substantial gender effects on muscular adaptations in response to PS plus RT, particularly for muscle strength and physical mobility. Our results are supported by the previous studies demonstrating sex-specific difference in treatment efficacy of PS plus exercise for muscle strength [119,152,190] and mobility (CR) [119] among older adults. Thirdly, the current NMR results showed positive associations between amount of protein supplement and leg strength gain, which indicated that PS induced augmentations of training effects on muscle strength might be dose dependent. Numeral previous studies have been observed that post-exercise increases in myofibrillar fractional synthesis rates responding to PS stimuli are significantly enhanced by an increased amount of protein intake in trained young or older adults [191,192,193]. Accordingly, such acute dose-dependent effects on muscle synthesis may lead to long-term neuromuscular adaptations following RT, particularly in terms of strength gain. In addition, our findings were agreed by the previous study concluding that ingestion of dietary protein as high as 1.6 g/kg/day significantly enhances leg extension strength compared with the minor intake (0.8 g/kg/day) in in elderly men [194]. Finally, we observed that a higher BMI was corresponded with minor changes in SPPB score whereas greater changes in SPPB score may be observed over a longer intervention or follow-up length up to 6 months. According to the present results, greater function recovery may occur at 6-month follow up, compared with that within a follow-up time of 3 months or shorter in older individuals who are not overweight or obese. Therefore, the restoration of physical mobility in response to PS plus RT appears to be time-dependent, especially for those who have normal body weight.

Participant compliance with PS was equally comparable among protocols of different protein sources in this NMA. The results indicated that older individuals tolerated well for all protein products derived from different sources. However, a relatively higher risk of occurrence rate of adverse events (OR = 3.04) in favor of WP was noted, comparing with that of other protein sources (OR = 1.13–2.04; Figure 9B). After consuming whey supplements, 45 adverse events in 45 participants were reported by 6 RCTs [89,97,98,108,126,140], most of which were related to gastrointestinal problems such as gastro esophageal reflux, nausea, bloating or diarrhea. Among the 45 participants who suffered WP-induced complications, 30 (66.7%) experienced mild to moderate severity of gastrointestinal distress and continued the supplementation by modifying dose, whereas the others (33.3%) happened severe gastrointestinal problems which needed to cease consuming whey supplements. Based on the results, we assume that most of the adverse events responding to WP are considered as nonserious and can be well treated despite of its higher occurrence rate.

Several limitations of the present NMA are needed to be noted. First, this NMA is limited by the varied study methodological quality with only 12 RCTs (15.4%) were ranked as low risk of bias, which restricts the strength of the results, and may lead to overestimation of relative effects among treatment regimens. Secondly, due to wide variation in PS (protein source, dosage, intake time) and RT protocols (training mode, duration, and volume) among included RCTs, it was difficult to make a definite protocol of specific regimen for treatment efficacy, even though WP + RT was ranked as the highest probability of treatment efficacy among all treatment arms. In addition, the optimal dosage of supplementation and training duration were not determined in the current NMA in spite of the fact that an increase of PS dose is associated with greater leg strength gain and a longer intervention is accompanied with greater effects on SPPB. Further researches are warranted to identify the optimal intake amount of protein supplements and training period, especially the WP + RT, for elder populations. Thirdly, significant publication biases of treatment effects were detected in muscle mass and leg strength, which downgrades the certainty of evidence. Therefore, the conclusions in these outcomes should be cautiously interpreted. Finally, some of the included RCTs conducted placebo supplementation in combination with RT or not. The placebo effects were not excluded for accounting the treatment effects of RT alone or RC groups. Therefore, the comparative effects among all treatment arms may be underestimated, as using the RC groups as the common reference.

## 5. Conclusions

The present NMA determined the comparative efficacy among different sources of PS for community-dwelling, hospitalized or institutionalized older individuals undergoing RT. Overall, the combined treatments of PS and RT were superior to its monotherapy. In addition, WP + RT was determined to be the optimal treatment regimen for muscle mass and strength gains as well as restoration of physical mobility with large effects and low to moderate certainty of evidence, despite of its relatively high risk of nonserious side effects. The treatment efficacy appears to be mediated by healthy status, sex, and body weight, and is likely to be dose and time dependent particularly for restorations of strength and physical mobility. Our findings add the knowledge on RS and RT intervention strategies, especially for older individuals with high sarcopenia and frailty risks. The results of this NMA may help the clinicians to prescribe the optimal PS protocol during RT intervention for ensuring optimal treatment outcomes.

## Figures and Tables

**Table 1:** Participant characteristics and study summary.

Study Arm	Combined Treatment			Resistance Training (Combined with or without Placebo Supplement)			Protein Supplementation Alone			Control (Placebo Supplement or Regular Care)		
Item	Trials (Groups), n ^a^	Sample (n)	Mean (Range) ^b^	Trials (Groups), n ^a^	Sample (n)	Mean (Range) ^b^	Trials (Groups), n ^a^	Sample (n)	Mean (Range) ^b^	Trials (Groups), n ^a^	Sample (n)	Mean (Range) ^b^
Age, year	78 (89)	2458	69.8 (47.0–87.2)	62 (64)	1664	69.8 (47.4–86.2)	19 (19)	415	68.6 (50.0–85.7)	27 (27)	735	73.8 (54.1–89.2)
Body mass index, kg/m^2^	74 (83)	2287	26.1 (18.8–32.7)	60 (62)	1624	26.9 (18.8–33.3)	18 (18)	400	25.5 (18.6–31.0)	25 (25)	592	24.9 (18.9–30.0)
Sex, n, %												
Sex-specific trial												
Men	22 (26)	573	100	18 (20)	332	100	4 (4)	91	100	8 (8)	288	100
Women	16 (17)	375	100	14 (14)	275	100	5 (5)	98	100	3 (3)	46	100
Sex mixed trial	39 (45)	1494		29 (29)	1052		10 (10)	226		16 (16)	401	
Men		614	42.3 (13.0–87.5)		422	41.5 (9.8–73.7)		89	40.0 (16.7–73.3)		175	44.8 (12.5–80.0)
Women		880	57.7 (12.5–87.0)		630	58.5 (26.3–90.2)		137	60.0 (26.7–83.3)		226	55.2 (20.0–87.5)
Population (area)												
America	29 (36)	669		25 (27)	434		6 (6)	107		7 (7)	108	
Asia	18 (18)	589		12 (12)	333		7 (7)	147		11 (11)	387	
Europe	23 (26)	710		17 (17)	469		5 (5)	116		6 (6)	109	
Oceania	8 (9)	490		8 (8)	428		1 (1)	45		3 (3)	131	
Physical condition												
Untrained healthy	33 (38)	947		27 (29)	692		8 (8)	202		9 (9)	199	
Dynapenia	4 (4)	92		3 (3)	78		2 (2)	39		3 (3)	51	
Frailty	12 (13)	394		12 (12)	356		2 (2)	44		5 (5)	122	
Sarcopenia	18 (20)	516		11 (11)	202		6 (6)	105		10 (10)	353	
Sedentary or inactive	7 (9)	213		5 (5)	100		2 (2)	25		1 (1)	10	
Overweight or obesity	5 (6)	296		5 (5)	236		0			0		
Protein source of supplementation												
Whey	43 (47)	1311					11 (11)	243				
Milk	17 (18)	553					2 (2)	73				
Soy	14 (14)	324					6 (6)	99				
Casein	4 (4)	94					0					
Meat	5 (5)	194					0					
Peanut	1 (1)	20					0					
Intervention compliance (%)												
Protein supplement	40 (47)	1287	91.5 (64–100)				37 (37)	323	87.8 (73–100)			
Resistance training	46 (52)	1505	87.3 (44–100)	38 (38)	1117	86.2 (44–100)						
Placebo supplement				15 (15)	300	93.8 (71.5–100)				4 (4)	55	89.4 (75–99)
Muscle mass (baseline)												
Total lean mass, kg	41 (47)	1354	45.9 (31.8–59.2)	36 (37)	1050	46.2 (32.0–60.8)	7 (7)	158	46.2 (39.9–56.6)	7 (7)	117	49.0 (47.9–51.5)
Appendicular lean mass, kg	38 (41)	1160	18.8 (7.9–30.5)	36 (36)	955	18.8 (8.0–30.0)	9 (9)	161	18.8 (12.8–27.2)	11 (11)	260	19.2 (12.6–26.7)
Total lean index, kg/m^2^	44 (50)	1406	17.2 (13.6–22.9)	39 (40)	1143	17.4 (13.9–22.5)	8 (8)	168	17.2 (15.5–19.0)	8 (8)	128	17.6 (16.6–19.1)
Appendicular lean index, kg/m^2^	39 (41)	1150	7.0 (3.0–9.4)	35 (35)	935	7.1 (3.0–9.4)	11 (11)	208	7.1 (5.4–8.8)	11 (11)	257	7.1 (5.2–8.9)
Handgrip strength (baseline), kg												
Sex-specific trial												
Men	7 (8)	282	30.3 (15.3–41.1)	4 (4)	64	33.2 (19.7–42.2)	2 (2)	37	25.5 (18.8–30.0)	3 (3)	190	18.6 (15.2–21.1)
Women	4 (5)	93	20.2 (15.8–22.4)	4 (4)	71	20.3 (15.1–23.1)	1 (1)	25	23	0		
Sex mixed trial	23 (25)	819	26.5 (13.8–41.2)	19 (19)	619	26.8 (15.8–41.8)	6 (6)	119	22.3 (16.4–35.6)	11 (11)	300	23.1 (15.1–33.6)
Physical mobility (baseline)												
5-time chair rise, s	28 (31)	1032	12.2 (4.6–26.5)	25 (25)	850	13.6 (4.7–30.0)	4 (4)	83	12.6 (7.7–18.7)	7 (7)	216	11.9 (7.8–18.6)
Walking speed, m/s	36 (39)	1277	1.26 (0.44–2.33)	31 (31)	956	1.18 (0.51–2.05)	13 (13)	322	1.25 (0.45–2.14)	15 (15)	407	1.26 (0.47–2.05)
Global mobility ^c^	16 (16)	402	9.0 (6.2–11.4)	10 (10)	181	9.3 (7.1–11.2)	1 (1)	20	6.2	7 (7)	224	8.6 (7.8–11.0)

^a^ Number of trials that reported the indicated item. ^b^ All summations calculated on the basis of the values reported in the analyzed studies that could be estimated. ^c^ The score is derived from the Short Physical Performance Battery.

**Table 2:** GRADE certainty rating of treatment options in all main outcomes.

Treatment (Common Comparator: Regular Care)	GRADE Certainty of Evidence ^a^						
	Muscle Mass	Muscle Strength		Physical Mobility			
		Handgrip	Leg Strength	Walking Speed	Chair Rise	Timed Up and Go	SPPB
Combined therapy							
Casein + RT	⨁⊝⊝⊝ ^b,d,e^	⨁⊝⊝⊝ ^b,c,d^	⨁⊝⊝⊝ ^b,d,e^				⨁⨁⊝⊝ ^b,e^
Meat + RT	⨁⨁⊝⊝ ^b,e^	⨁⨁⊝⊝ ^b,d^	⨁⨁⊝⊝ ^b,e^	⨁⨁⊝⊝ ^b,d^	⨁⨁⊝⊝ ^b,d^	⨁⨁⊝⊝ ^b,d^	
Milk + RT	⨁⨁⊝⊝ ^b,e^	⨁⨁⨁⊝ ^b^	⨁⨁⊝⊝ ^b,e^	⨁⨁⨁⊝ ^b^	⨁⨁⨁⊝ ^b^	⨁⨁⊝⊝ ^b,d^	⨁⨁⨁⊝ ^b^
Peanut + RT	⨁⊝⊝⊝ ^b,d,e^						
Soy + RT	⨁⨁⊝⊝ ^b,e^	⨁⨁⨁⨁	⨁⊝⊝⊝ ^b,c,e^	⨁⨁⊝⊝ ^b,d^	⨁⨁⨁⨁	⨁⨁⊝⊝ ^b,d^	
Whey + RT	⨁⨁⊝⊝ ^b,e^	⨁⨁⨁⊝ ^b^	⨁⨁⊝⊝ ^b,e^	⨁⨁⨁⊝ ^b^	⨁⨁⨁⊝ ^b^	⨁⨁⊝⊝ ^b,d^	⨁⨁⊝⊝ ^b,c^
Monotherapy							
Meat	⨁⊝⊝⊝ ^b,d,e^		⨁⊝⊝⊝ ^b,d,e^				
Milk	⨁⊝⊝⊝ ^b,d,e^		⨁⊝⊝⊝ ^b,d,e^	⨁⨁⊝⊝ ^b,d^	⨁⨁⊝⊝ ^b,d^		
Soy	⨁⊝⊝⊝ ^b,d,e^	⨁⨁⨁⊝ ^d^	⨁⊝⊝⊝ ^b,c,d,e^	⨁⨁⊝⊝ ^b,d^			
Whey	⨁⊝⊝⊝ ^b,d,e^	⨁⨁⊝⊝ ^b,d^	⨁⊝⊝⊝ ^b,d,e^	⨁⨁⊝⊝ ^b,c^	⨁⨁⊝⊝ ^b,d^		⨁⨁⨁⊝ ^b^
Resistance training	⨁⨁⊝⊝ ^b,e^	⨁⨁⨁⊝ ^b^	⨁⨁⊝⊝ ^b,e^	⨁⨁⨁⊝ ^b^	⨁⨁⨁⊝ ^b^	⨁⨁⊝⊝ ^b,d^	⨁⨁⨁⊝ ^b^

^a^ Certainty of evidence is graded as follows: High: ⨁⨁⨁⨁; Moderate: ⨁⨁⨁⊝; Low: ⨁⨁⊝⊝; Very low: ⨁⊝⊝⊝. ^b^ Network contribution of high risk of bias ≥ 50%. ^c^ There is significant difference between direct and indirect estimates. ^d^ 95% confidence interval is wide and imprecise. ^e^ Test for publication bias is statistically significant. GRADE, Grading of Recommendations, Assessment, Development and Evaluations; SPPB, Short Physical Performance Battery.

## Data Availability

Refer to Supplementary Materials. Raw data available on request.

## Supplementary Materials

The following supporting information can be downloaded at: https://www.mdpi.com/article/10.3390/nu16070941/s1, Table S1. Database search formulas; Table S2. Summary of included study characteristics; Table S3. League table for pairwise and network meta-analysis of mean change in muscle mass from baseline; Table S4. League table for pairwise and network meta-analysis of mean change in handgrip strength from baseline; Table S5. League table for pairwise and network meta-analysis of mean change in leg strength from baseline; Table S6. League table for pairwise and network meta-analysis of mean change in walking speed from baseline; Table S7. League table for pairwise and network meta-analysis of mean change in chair rise from baseline; Table S8. League table for pairwise and network meta-analysis of mean change in global physical mobility (SPPB) from baseline; Table S9. League table for pairwise and network meta-analysis of mean change in timed up-and-go task from baseline; Table S10. Associations of moderators with treatment efficiency for main outcomes; Table S11. Summary of compliance & adverse events; Table S12. Judgments in each domain of GRADE ranking system for muscle mass outcome; Table S13. Judgments in each domain of GRADE framework for handgrip strength outcome; Table S14. Judgments in each domain of GRADE framework for leg strength outcome; Table S15. Judgments in each domain of GRADE framework for walking speed outcome; Table S16. Judgments in each domain of GRADE framework for chair rise outcome; Table S17. Judgments in each domain of GRADE framework for timed up-and-go outcome; Table S18. Judgments in each domain of GRADE framework for timed global mobility (SPPB) outcome; Figure S1. Risk of bias summary for each trial; Figure S2. Forest plot of node-splitting results for muscle mass; Figure S3. Relative effects among treatment regimens for muscle mass gain at each follow-up time frame; Figure S4. Forest plot of node-splitting results for handgrip strength; Figure S5. Forest plot of node-splitting results for leg strength; Figure S6. Relative effects among treatment regimens for handgrip strength at each follow-up time frame; Figure S7. Relative effects among treatment regimens for leg strength at each follow-up time frame; Figure S8. Relative effects among treatment regimens for walking speed at each follow-up time frame; Figure S9. Forest plot of node-splitting results for walking speed; Figure S10. Relative effects among treatment regimens for chair rise at each follow-up time frame; Figure S11. Forest plot of node-splitting results for chair rise; Figure S12. Relative effects among treatment regimens for timed up-and-go task at each follow-up time frame; Figure S13. Forest plot of node-splitting results for TUG; Figure S14. Relative effects among treatment regimens for Short Physical Performance Battery at each follow-up time frame; Figure S15. Forest plot of node-splitting results for SPPB; Figure S16. Funnel plots of the intervention effects for (A) muscle mass, (B) handgrip strength, (C) leg muscle strength, (D) walk speed, (E) chair rise ability, (F) timed up and go, and (G) SPPB.
