# Supplementary material for: Comparative Efficacy of Different Protein Supplements on Muscle Mass, Strength, and Physical Indices of Sarcopenia among Community-Dwelling, Hospitalized or Institutionalized Older Adults Undergoing Resistance Training: A Network Meta-Analysis of Randomized Controlled Trials
Source: Nutrients. 2024 Mar 25;16(7):941. doi: 10.3390/nu16070941 (PMC11013298; doi:10.3390/nu16070941)

Figure S1. Risk of bias summary for each trial

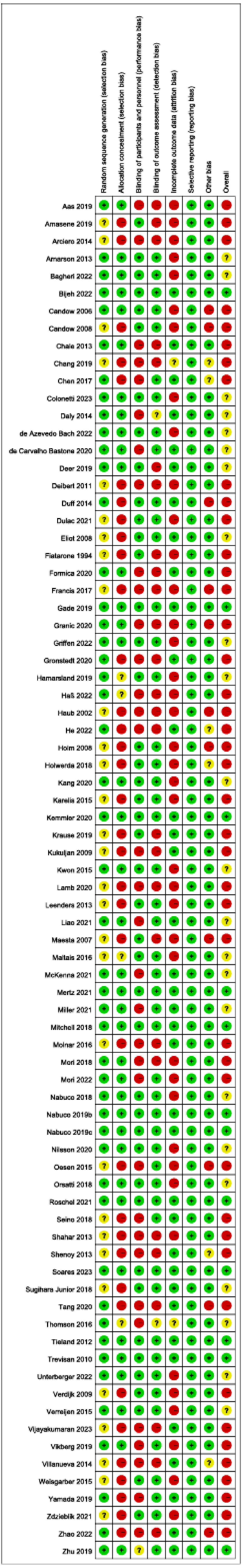

Figure S2. Forest plot of node-splitting results for muscle mass

Comparison	Number of Studies	Direct Evidence	I2	SMD	95%CI
**Casein+RT:RT**					
Direct estimate	1	0.46		0.39	[−1.03; 1.80]
Indirect estimate				−0.03	[−1.33; 1.27]
Network estimate				0.16	[−0.80; 1.12]
**Casein+RT:WP+RT**					
Direct estimate	1	0.56		−0.60	[−1.88; 0.68]
Indirect estimate				−0.19	[−1.62; 1.24]
Network estimate				−0.42	[−1.37; 0.54]
**Meat+RT:RT**					
Direct estimate	2	0.75	0%	0.40	[−0.48; 1.28]
Indirect estimate				0.88	[−0.64; 2.40]
Network estimate				0.52	[−0.24; 1.28]
**Meat+RT:SP+RT**					
Direct estimate	1	0.30		0.51	[−0.96; 1.97]
Indirect estimate				0.03	[−0.94; 0.99]
Network estimate				0.17	[−0.64; 0.98]
**MP:MP+RT**					
Direct estimate	3	0.68	12%	−0.62	[−1.39; 0.14]
Indirect estimate				−1.15	[−2.28; −0.02]
Network estimate				−0.79	[−1.42; −0.16]
**MP:RC**					
Direct estimate	2	0.48	30%	0.31	[−0.62; 1.24]
Indirect estimate				0.58	[−0.30; 1.47]
Network estimate				0.45	[−0.19; 1.09]
**MP:RT**					
Direct estimate	1	0.25		−0.36	[−1.61; 0.90]
Indirect estimate				−0.22	[−0.95; 0.51]
Network estimate				−0.26	[−0.89; 0.38]
**MP:SP**					
Direct estimate	1	0.28		0.19	[−1.28; 1.65]
Indirect estimate				0.46	[−0.45; 1.36]
Network estimate				0.38	[−0.39; 1.15]
**MP:SP+RT**					
Direct estimate	1	0.24		−0.56	[−1.99; 0.87]
Indirect estimate				−0.62	[−1.41; 0.17]
Network estimate				−0.60	[−1.30; 0.09]
**MP+RT:RC**					
Direct estimate	2	0.20	0%	1.31	[0.41; 2.22]
Indirect estimate				1.22	[0.77; 1.68]
Network estimate				1.24	[0.83; 1.65]
**MP+RT:RT**					
Direct estimate	12	0.73	47%	0.55	[0.17; 0.93]
Indirect estimate				0.49	[−0.13; 1.12]
Network estimate				0.53	[0.21; 0.86]
**MP+RT:SP**					
Direct estimate	1	0.17		0.75	[−0.72; 2.22]
Indirect estimate				1.26	[0.60; 1.91]
Network estimate				1.17	[0.57; 1.77]
**MP+RT:SP+RT**					
Direct estimate	2	0.24	0%	−0.20	[−1.16; 0.76]
Indirect estimate				0.31	[−0.23; 0.84]
Network estimate				0.19	[−0.28; 0.65]
**MP+RT:WP+RT**					
Direct estimate	2	0.15	0%	0.36	[−0.61; 1.32]
Indirect estimate				−0.12	[−0.52; 0.28]
Network estimate				−0.05	[−0.42; 0.32]
**RT:RC**					
Direct estimate	15	0.71	79%	0.79	[0.42; 1.15]
Indirect estimate				0.51	[−0.06; 1.09]
Network estimate				0.71	[0.40; 1.02]
**SP:RC**					
Direct estimate	4	0.66	0%	0.19	[−0.49; 0.87]
Indirect estimate				−0.17	[−1.11; 0.78]
Network estimate				0.07	[−0.48; 0.62]
**SP+RT:RC**					
Direct estimate	6	0.57	92%	1.06	[0.49; 1.63]
Indirect estimate				1.05	[0.40; 1.70]
Network estimate				1.06	[0.63; 1.49]
**WP:RC**					
Direct estimate	8	0.72	58%	0.31	[−0.18; 0.81]
Indirect estimate				0.46	[−0.33; 1.26]
Network estimate				0.36	[−0.07; 0.78]
**WP+RT:RC**					
Direct estimate	10	0.50	90%	1.36	[0.90; 1.83]
Indirect estimate				1.21	[0.74; 1.68]
Network estimate				1.29	[0.96; 1.62]
**RT:SP**					
Direct estimate	4	0.62	81%	0.77	[0.08; 1.45]
Indirect estimate				0.43	[−0.46; 1.31]
Network estimate				0.64	[0.10; 1.18]
**RT:SP+RT**					
Direct estimate	9	0.73	85%	−0.40	[−0.85; 0.05]
Indirect estimate				−0.21	[−0.96; 0.54]
Network estimate				−0.35	[−0.74; 0.04]
**RT:WP**					
Direct estimate	7	0.53	83%	0.09	[−0.45; 0.63]
Indirect estimate				0.64	[0.07; 1.21]
Network estimate				0.35	[−0.04; 0.74]
**RT:WP+RT**					
Direct estimate	36	0.88	62%	−0.56	[−0.79; −0.33]
Indirect estimate				−0.75	[−1.36; −0.13]
Network estimate				−0.58	[−0.80; −0.36]
**SP:SP+RT**					
Direct estimate	5	0.81	92%	−1.21	[−1.85; −0.58]
Indirect estimate				−0.04	[−1.35; 1.27]
Network estimate				−0.99	[−1.56; −0.42]
**SP+RT:WP+RT**					
Direct estimate	1	0.10		−0.12	[−1.45; 1.20]
Indirect estimate				−0.24	[−0.69; 0.21]
Network estimate				−0.23	[−0.66; 0.19]
**WP:WP+RT**					
Direct estimate	11	0.82	87%	−0.95	[−1.37; −0.52]
Indirect estimate				−0.86	[−1.76; 0.05]
Network estimate				−0.93	[−1.32; −0.55]

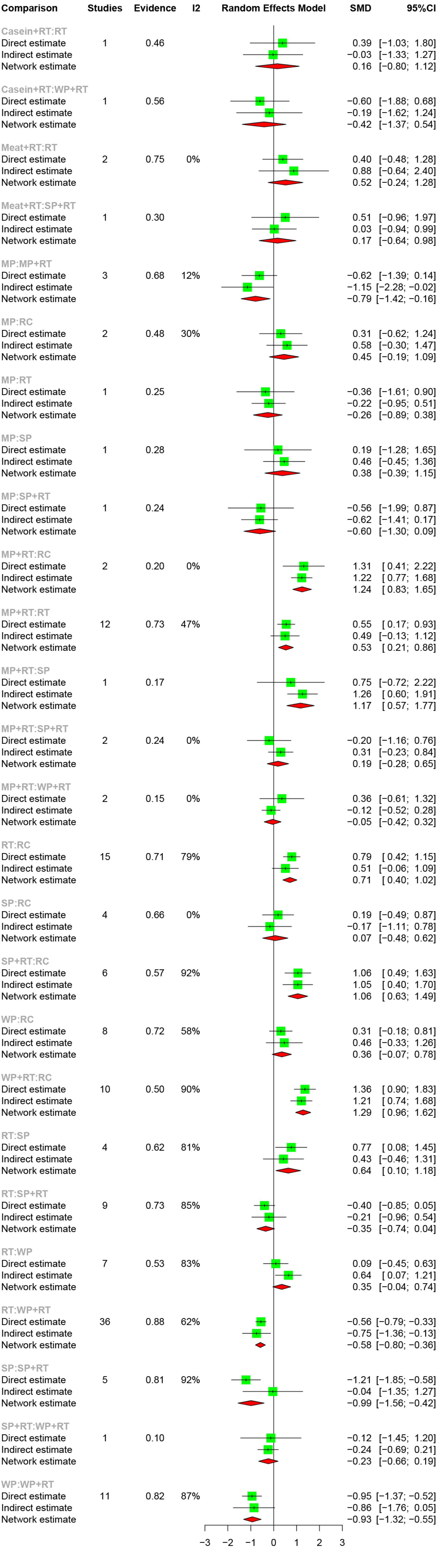

Supplementary Figure S3. Relative effects among treatment regimens for muscle mass gain at each follow-up time frame

Follow up ≤3 months

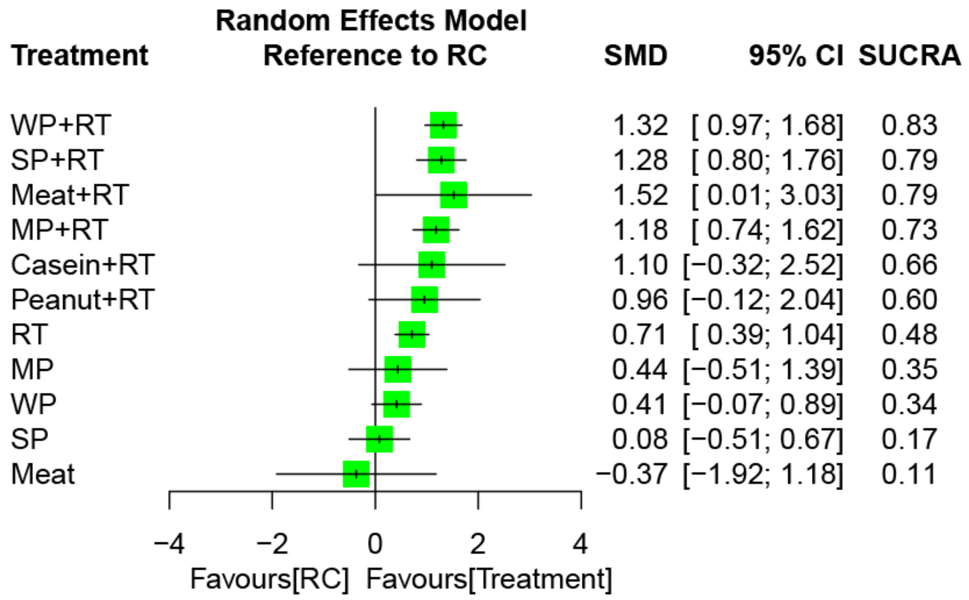

Follow up >3 months, ≤6 months

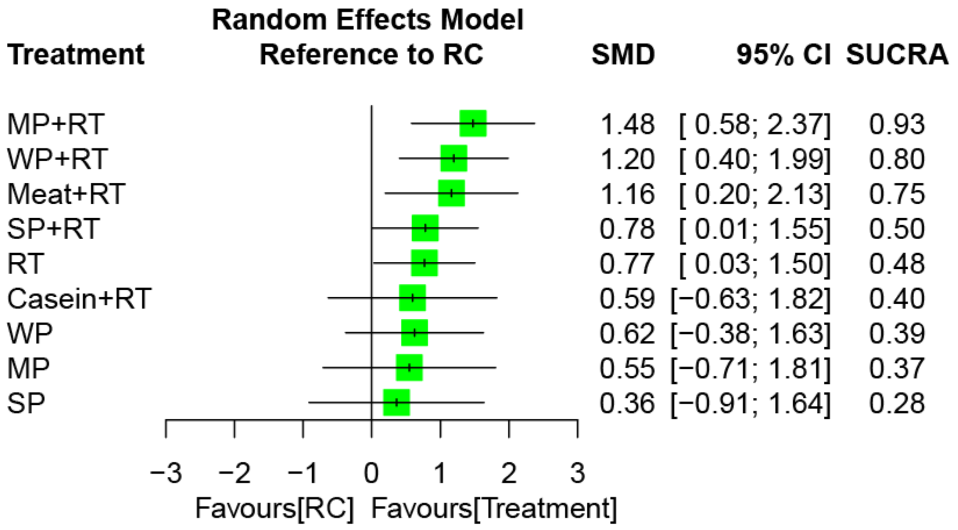

Follow up >6 months

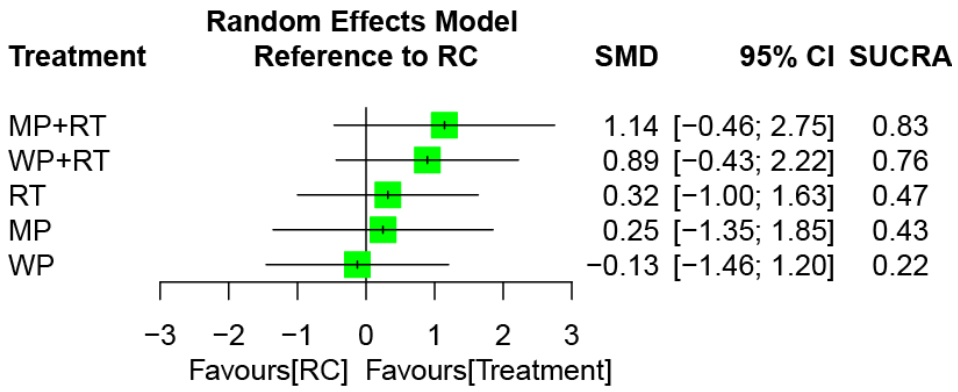

Figure S4. Forest plot of node-splitting results for handgrip strength

Comparison	Number of Studies	Direct Evidence	I2	SMD	95%CI
Casein+RT:RC					
Direct estimate	1	0.44		2.98	[0.99; 4.97]
Indirect estimate				−0.13	[−1.90; 1.64]
Network estimate				1.24	[−0.08; 2.56]
Casein+RT:RT					
Direct estimate	1	0.46		−0.58	[−2.52; 1.37]
Indirect estimate				0.97	[−0.84; 2.78]
Network estimate				0.25	[−1.07; 1.58]
Casein+RT:WP+RT					
Direct estimate	1	0.45		−1.93	[−3.90; 0.04]
Indirect estimate				1.20	[−0.59; 2.99]
Network estimate				−0.22	[−1.55; 1.11]
Meat+RT:RC					
Direct estimate	1	0.78		−0.88	[−2.82; 1.05]
Indirect estimate				−0.80	[−4.45; 2.85]
Network estimate				−0.87	[−2.57; 0.84]
Meat+RT:RT					
Direct estimate	1	0.77		−1.83	[−3.79; 0.12]
Indirect estimate				−1.91	[−5.45; 1.62]
Network estimate				−1.85	[−3.56; −0.14]
MP+RT:RC					
Direct estimate	1	0.16		0.22	[−1.68; 2.13]
Indirect estimate				1.48	[0.64; 2.32]
Network estimate				1.27	[0.50; 2.04]
MP+RT:RT					
Direct estimate	9	0.88	96%	0.39	[−0.26; 1.04]
Indirect estimate				−0.50	[−2.28; 1.28]
Network estimate				0.29	[−0.32; 0.89]
MP+RT:SP+RT					
Direct estimate	1	0.27		−0.18	[−2.09; 1.73]
Indirect estimate				−0.10	[−1.27; 1.07]
Network estimate				−0.12	[−1.12; 0.88]
RT:RC					
Direct estimate	8	0.61	64%	0.56	[−0.13; 1.26]
Indirect estimate				1.65	[0.78; 2.52]
Network estimate				0.99	[0.44; 1.53]
SP:RC					
Direct estimate	1	0.71		0.90	[−1.08; 2.88]
Indirect estimate				−1.17	[−4.24; 1.91]
Network estimate				0.29	[−1.37; 1.96]
SP+RT:RC					
Direct estimate	2	0.44	98%	1.51	[0.11; 2.90]
Indirect estimate				1.31	[0.06; 2.56]
Network estimate				1.40	[0.47; 2.33]
WP:RC					
Direct estimate	5	0.68	0%	0.12	[−0.77; 1.01]
Indirect estimate				1.19	[−0.09; 2.48]
Network estimate				0.47	[−0.26; 1.20]
WP+RT:RC					
Direct estimate	9	0.68	95%	1.53	[0.88; 2.19]
Indirect estimate				1.30	[0.34; 2.26]
Network estimate				1.46	[0.92; 2.00]
RT:SP					
Direct estimate	1	0.70		0.03	[−1.94; 2.00]
Indirect estimate				2.24	[−0.78; 5.27]
Network estimate				0.69	[−0.96; 2.34]
RT:SP+RT					
Direct estimate	4	0.79	91%	−0.45	[−1.42; 0.52]
Indirect estimate				−0.26	[−2.16; 1.64]
Network estimate				−0.41	[−1.27; 0.45]
RT:WP					
Direct estimate	5	0.62	62%	0.38	[−0.51; 1.27]
Indirect estimate				0.74	[−0.40; 1.89]
Network estimate				0.52	[−0.18; 1.22]
RT:WP+RT					
Direct estimate	17	0.87	56%	−0.31	[−0.79; 0.16]
Indirect estimate				−1.60	[−2.85; −0.35]
Network estimate				−0.47	[−0.92; −0.03]
SP:SP+RT					
Direct estimate	1	0.72		−2.46	[−4.48; −0.44]
Indirect estimate				2.40	[−0.85; 5.64]
Network estimate				−1.10	[−2.82; 0.61]
SP+RT:WP+RT					
Direct estimate	1	0.23		−0.43	[−2.36; 1.50]
Indirect estimate				0.04	[−1.00; 1.09]
Network estimate				−0.06	[−0.98; 0.85]
WP:WP+RT					
Direct estimate	7	0.82	81%	−0.86	[−1.61; −0.11]
Indirect estimate				−1.57	[−3.16; 0.01]
Network estimate				−0.99	[−1.67; −0.31]

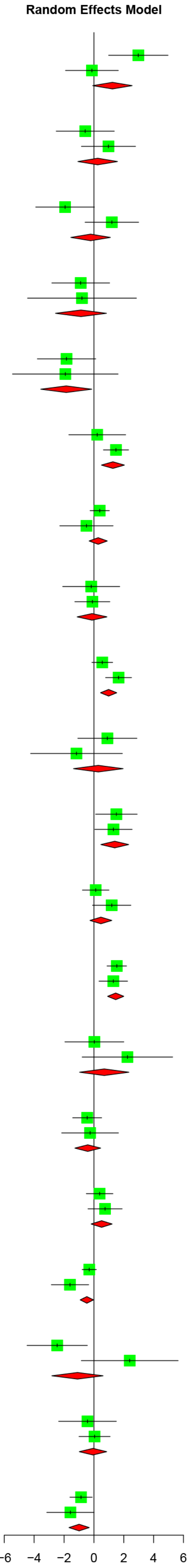

Figure S5. Forest plot of node-splitting results for leg strength

Comparison	Number of Studies	Direct Evidence	I2	SMD	95%CI
**Meat+RT vs RT**					
Direct estimate	2	0.76	0%	0.13	[−0.68; 0.94]
Indirect estimate				0.81	[−0.63; 2.25]
Network estimate				0.29	[−0.41; 1.00]
**Meat+RT vs SP+RT**					
Direct estimate	1	0.30		0.42	[−0.96; 1.81]
Indirect estimate				−0.25	[−1.15; 0.64]
Network estimate				−0.05	[−0.81; 0.70]
**MP vs MP+RT**					
Direct estimate	2	0.67	0%	−0.70	[−1.54; 0.13]
Indirect estimate				−1.66	[−2.86; −0.46]
Network estimate				−1.02	[−1.70; −0.33]
**MP vs RC**					
Direct estimate	2	0.64	0%	−0.05	[−0.91; 0.82]
Indirect estimate				0.94	[−0.22; 2.09]
Network estimate				0.31	[−0.38; 1.00]
**MP vs RT**					
Direct estimate	1	0.35		−0.47	[−1.63; 0.69]
Indirect estimate				−0.78	[−1.63; 0.07]
Network estimate				−0.67	[−1.36; 0.02]
**MP+RT vs RC**					
Direct estimate	1	0.16		0.69	[−0.47; 1.85]
Indirect estimate				1.45	[0.94; 1.97]
Network estimate				1.33	[0.86; 1.80]
**MP+RT vs RT**					
Direct estimate	9	0.76	72%	0.34	[−0.08; 0.76]
Indirect estimate				0.38	[−0.36; 1.12]
Network estimate				0.35	[−0.02; 0.71]
**MP+RT vs SP+RT**					
Direct estimate	1	0.17		0.32	[−0.87; 1.52]
Indirect estimate				−0.07	[−0.61; 0.48]
Network estimate				0.00	[−0.50; 0.50]
**MP+RT vs WP+RT**					
Direct estimate	2	0.20	80%	0.12	[−0.79; 1.02]
Indirect estimate				−0.13	[−0.59; 0.32]
Network estimate				−0.08	[−0.49; 0.32]
**RT vs RC**					
Direct estimate	10	0.70	75%	0.94	[0.53; 1.36]
Indirect estimate				1.06	[0.41; 1.70]
Network estimate				0.98	[0.63; 1.33]
**SP vs RC**					
Direct estimate	3	0.78	81%	0.41	[−0.32; 1.14]
Indirect estimate				−1.31	[−2.68; 0.07]
Network estimate				0.03	[−0.61; 0.68]
**SP+RT vs RC**					
Direct estimate	5	0.56	93%	1.73	[1.14; 2.32]
Indirect estimate				0.80	[0.13; 1.47]
Network estimate				1.32	[0.88; 1.77]
**WP vs RC**					
Direct estimate	4	0.64	74%	0.20	[−0.46; 0.85]
Indirect estimate				0.07	[−0.80; 0.94]
Network estimate				0.15	[−0.37; 0.67]
**WP+RT vs RC**					
Direct estimate	6	0.45	88%	1.30	[0.73; 1.87]
Indirect estimate				1.50	[0.99; 2.01]
Network estimate				1.41	[1.03; 1.79]
**RT vs SP**					
Direct estimate	2	0.53	0%	0.58	[−0.31; 1.47]
Indirect estimate				1.35	[0.41; 2.28]
Network estimate				0.94	[0.30; 1.59]
**RT vs SP+RT**					
Direct estimate	9	0.79	67%	−0.14	[−0.57; 0.29]
Indirect estimate				−1.09	[−1.91; −0.27]
Network estimate				−0.35	[−0.72; 0.03]
**RT vs WP**					
Direct estimate	4	0.52	85%	0.64	[−0.03; 1.32]
Indirect estimate				1.02	[0.32; 1.72]
Network estimate				0.83	[0.34; 1.31]
**RT vs WP+RT**					
Direct estimate	28	0.89	64%	−0.45	[−0.69; −0.20]
Indirect estimate				−0.31	[−1.01; 0.38]
Network estimate				−0.43	[−0.66; −0.20]
**SP vs SP+RT**					
Direct estimate	3	0.75	93%	−1.92	[−2.68; −1.16]
Indirect estimate				0.57	[−0.73; 1.88]
Network estimate				−1.29	[−1.95; −0.63]
**SP+RT vs WP+RT**					
Direct estimate	1	0.12		−0.74	[−1.98; 0.50]
Indirect estimate				0.00	[−0.45; 0.45]
Network estimate				−0.09	[−0.51; 0.34]
**WP vs WP+RT**					
Direct estimate	6	0.80	87%	−1.30	[−1.84; −0.76]
Indirect estimate				−1.09	[−2.15; −0.04]
Network estimate				−1.26	[−1.74; −0.78]

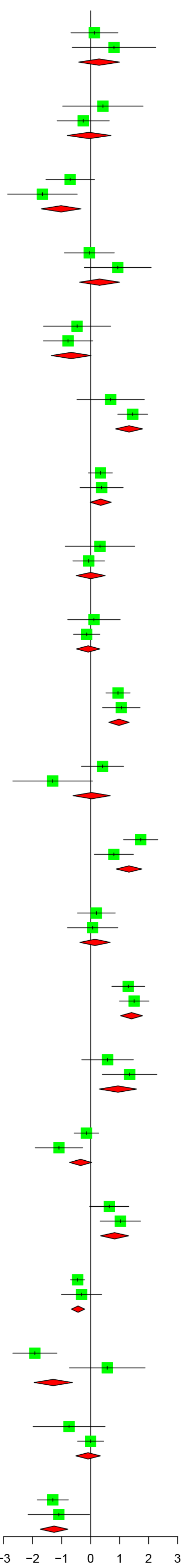

Supplementary Figure S6. Relative effects among treatment regimens for handgrip strength at each follow-up time frame

Follow up ≤3 months

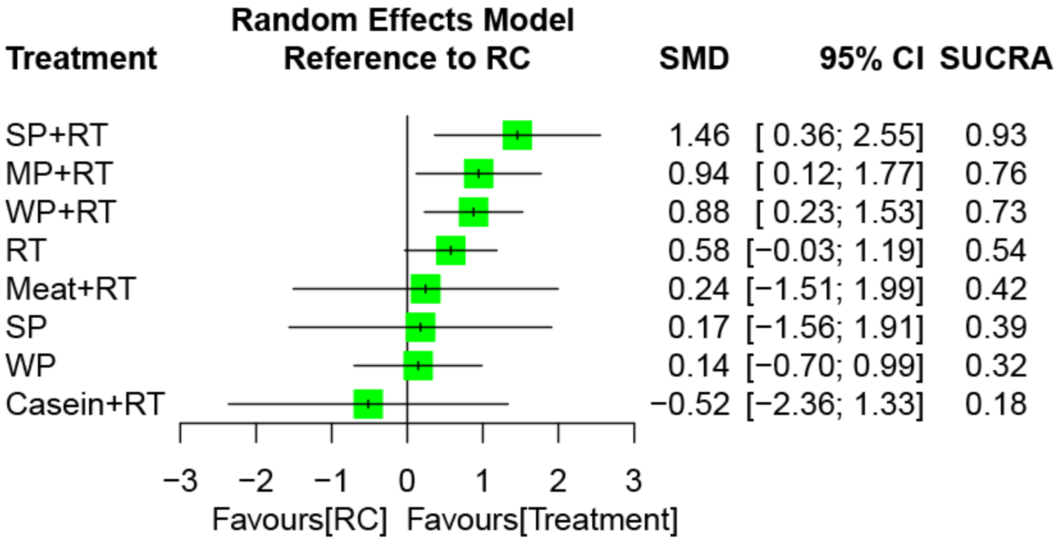

Follow up >3 months, ≤6 months

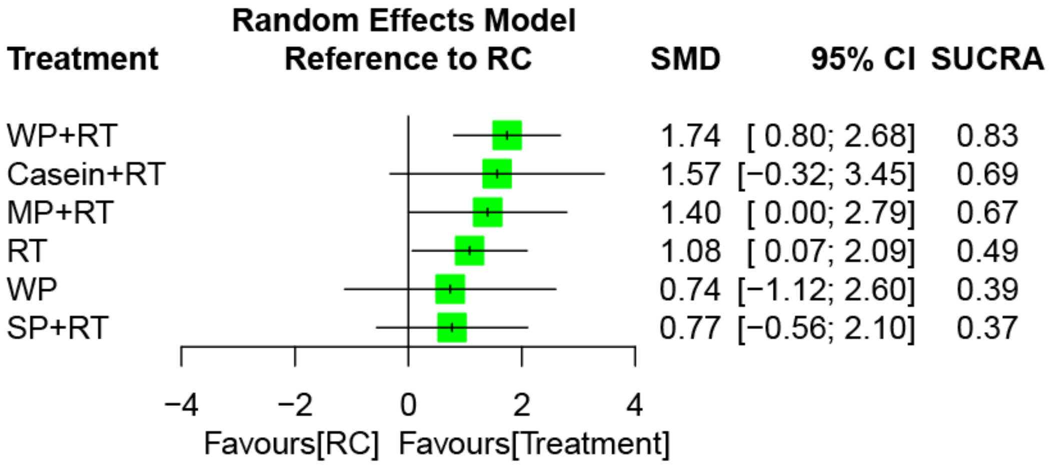

Follow up >6 months

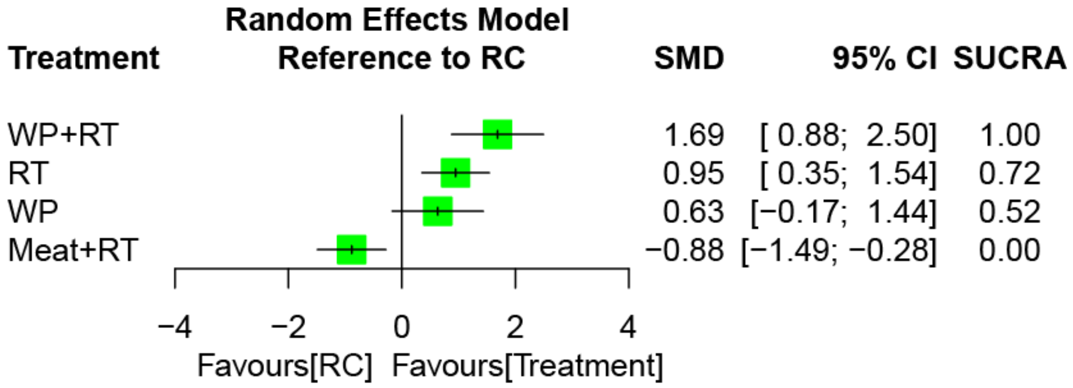

Supplementary Figure S7. Relative effects among treatment regimens for leg strength at each follow-up time frame

Follow up ≤3 months

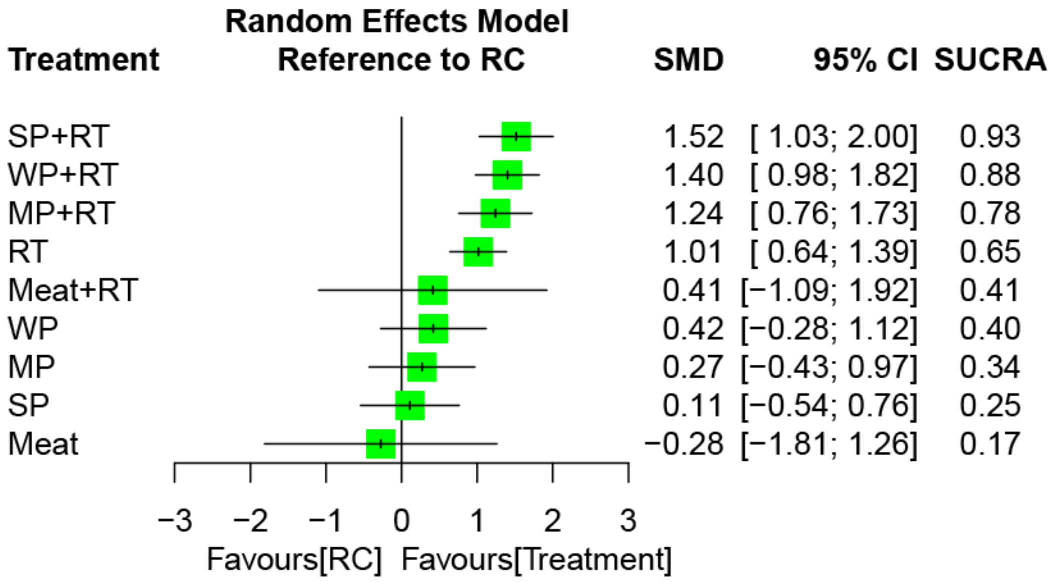

Follow up >3 months, ≤6 months

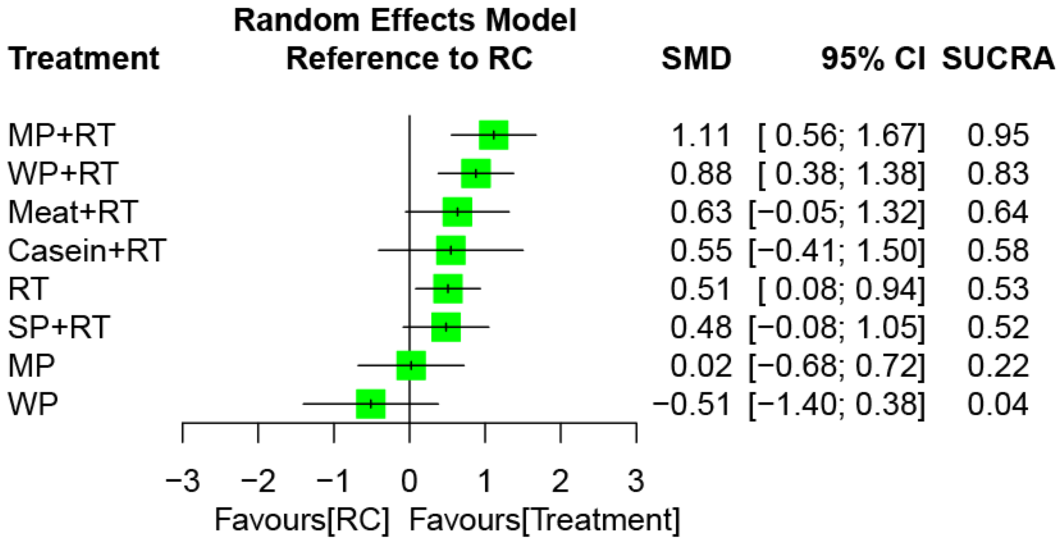

Follow up >6 months

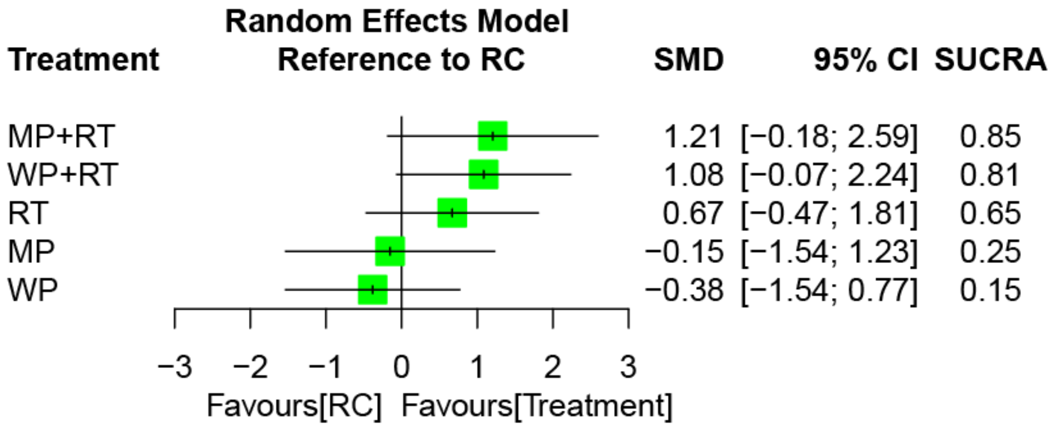

Supplementary Figure S8. Relative effects among treatment regimens for walking speed at each follow-up time frame

Follow up ≤3 months

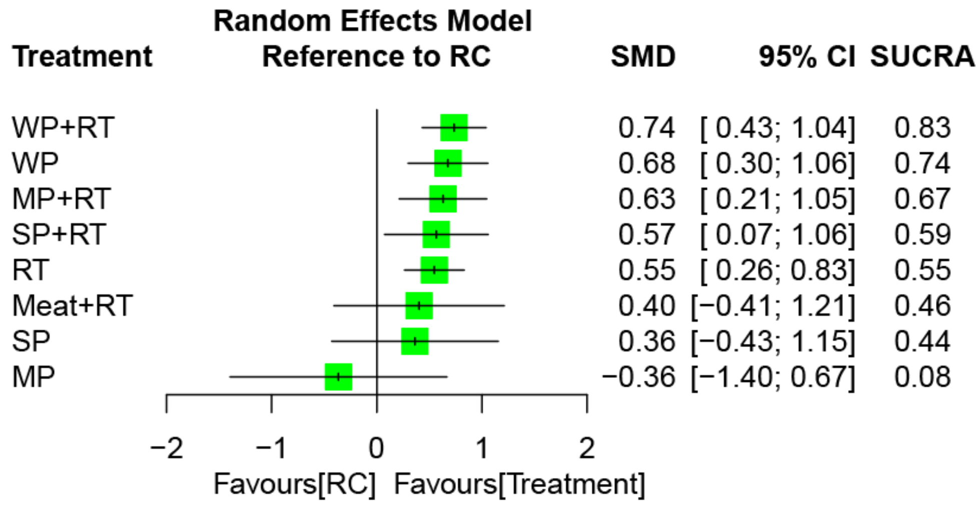

Follow up >3 months, ≤6 months

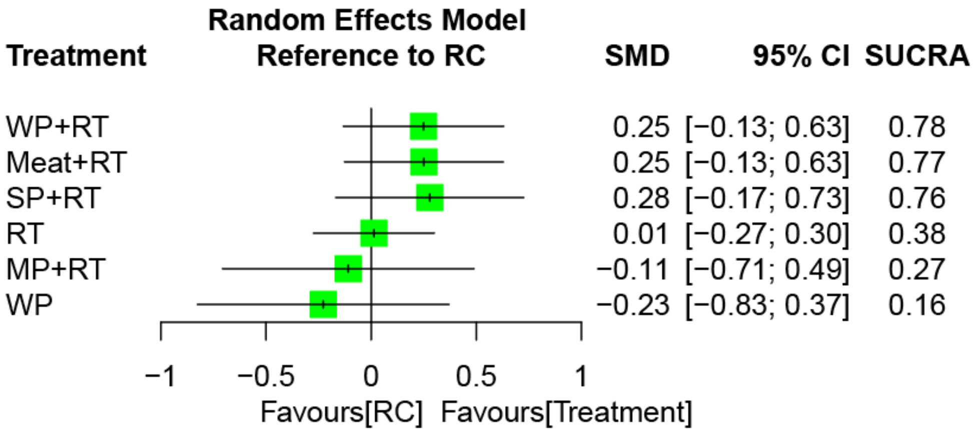

Follow up >6 months

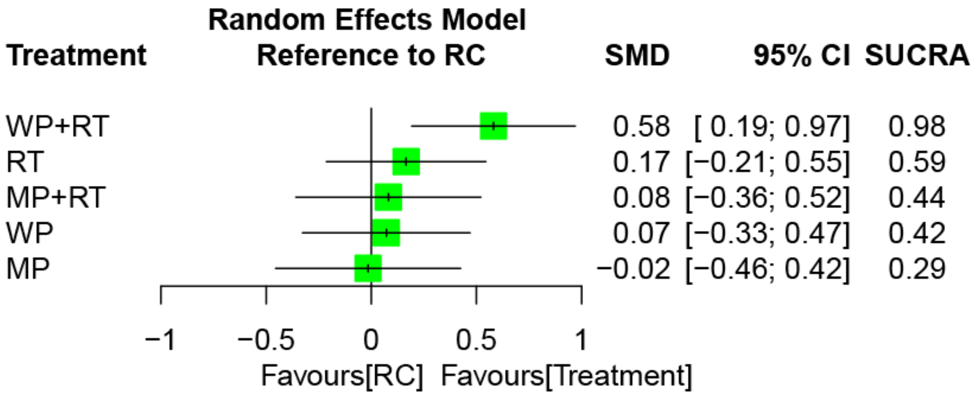

Overall follow up duration

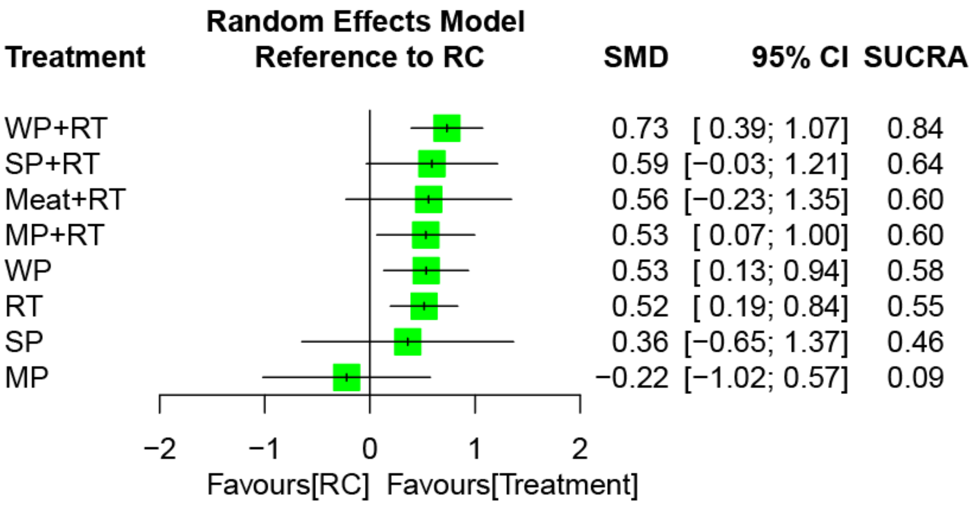

Figure S9. Forest plot of node-splitting results for walking speed

Comparison	Number of Studies	Direct Evidence	I2	SMD	95%CI
**Meat+RT:RC**					
Direct estimate	1	0.44		0.04	[−1.15; 1.22]
Indirect estimate				0.97	[−0.08; 2.02]
Network estimate				0.56	[−0.23; 1.35]
**Meat+RT:RT**					
Direct estimate	2	0.88	0%	0.20	[−0.60; 1.01]
Indirect estimate				−1.11	[−3.27; 1.06]
Network estimate				0.04	[−0.71; 0.80]
**MP:MP+RT**					
Direct estimate	2	0.83	52%	−0.72	[−1.54; 0.11]
Indirect estimate				−0.93	[−2.76; 0.89]
Network estimate				−0.75	[−1.50; −0.00]
**MP:RC**					
Direct estimate	1	0.50		−0.28	[−1.41; 0.85]
Indirect estimate				−0.16	[−1.28; 0.96]
Network estimate				−0.22	[−1.02; 0.57]
**MP:RT**					
Direct estimate	1	0.46		−0.53	[−1.66; 0.60]
Indirect estimate				−0.91	[−1.96; 0.14]
Network estimate				−0.74	[−1.51; 0.03]
**MP+RT:RC**					
Direct estimate	1	0.17		0.19	[−0.94; 1.32]
Indirect estimate				0.60	[0.09; 1.11]
Network estimate				0.53	[0.07; 1.00]
**MP+RT:RT**					
Direct estimate	8	0.83	10%	−0.04	[−0.46; 0.37]
Indirect estimate				0.32	[−0.61; 1.24]
Network estimate				0.02	[−0.36; 0.39]
**MP+RT:SP+RT**					
Direct estimate	1	0.33		0.27	[−0.89; 1.44]
Indirect estimate				−0.22	[−1.03; 0.59]
Network estimate				−0.06	[−0.73; 0.61]
**MP+RT:WP+RT**					
Direct estimate	1	0.13		0.29	[−0.93; 1.51]
Indirect estimate				−0.27	[−0.75; 0.20]
Network estimate				−0.20	[−0.64; 0.24]
**RT:RC**					
Direct estimate	12	0.77	78%	0.59	[0.22; 0.95]
Indirect estimate				0.28	[−0.39; 0.95]
Network estimate				0.52	[0.19; 0.84]
**SP:RC**					
Direct estimate	1	0.72		0.47	[−0.72; 1.66]
Indirect estimate				0.08	[−1.82; 1.97]
Network estimate				0.36	[−0.65; 1.37]
**SP+RT:RC**					
Direct estimate	2	0.57	57%	0.55	[−0.28; 1.37]
Indirect estimate				0.65	[−0.30; 1.60]
Network estimate				0.59	[−0.03; 1.21]
**WP:RC**					
Direct estimate	8	0.77	82%	0.76	[0.30; 1.22]
Indirect estimate				−0.21	[−1.05; 0.62]
Network estimate				0.53	[0.13; 0.94]
**WP+RT:RC**					
Direct estimate	10	0.68	80%	0.84	[0.43; 1.25]
Indirect estimate				0.50	[−0.11; 1.10]
Network estimate				0.73	[0.39; 1.07]
**RT:SP**					
Direct estimate	1	0.71		0.13	[−1.06; 1.32]
Indirect estimate				0.22	[−1.64; 2.08]
Network estimate				0.16	[−0.85; 1.16]
**RT:SP+RT**					
Direct estimate	3	0.79	0%	−0.22	[−0.90; 0.46]
Indirect estimate				0.45	[−0.84; 1.75]
Network estimate				−0.07	[−0.67; 0.53]
**RT:WP**					
Direct estimate	7	0.61	26%	−0.18	[−0.67; 0.31]
Indirect estimate				0.23	[−0.39; 0.84]
Network estimate				−0.02	[−0.40; 0.36]
**RT:WP+RT**					
Direct estimate	18	0.86	51%	−0.20	[−0.49; 0.09]
Indirect estimate				−0.31	[−1.03; 0.42]
Network estimate				−0.22	[−0.49; 0.05]
**SP:SP+RT**					
Direct estimate	1	0.78		−0.37	[−1.56; 0.82]
Indirect estimate				0.25	[−1.97; 2.47]
Network estimate				−0.23	[−1.28; 0.82]
**WP:WP+RT**					
Direct estimate	10	0.86	55%	−0.31	[−0.70; 0.09]
Indirect estimate				0.46	[−0.53; 1.44]
Network estimate				−0.20	[−0.57; 0.17]

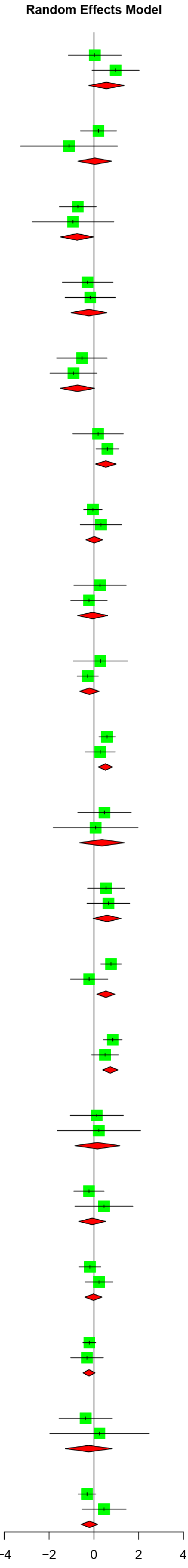

Supplementary Figure S10. Relative effects among treatment regimens for chair rise at each follow-up time frame

Follow up ≤3 months

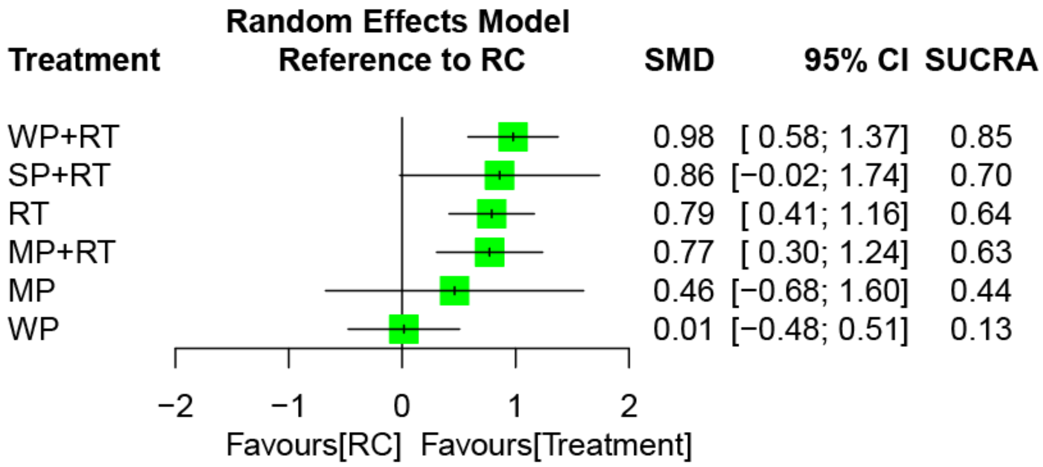

Follow up >3 months, ≤6 months

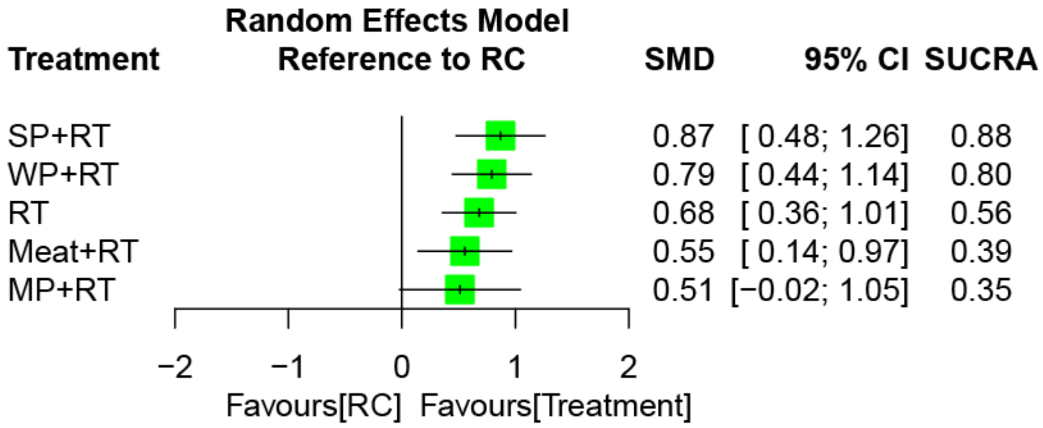

Overall follow up duration

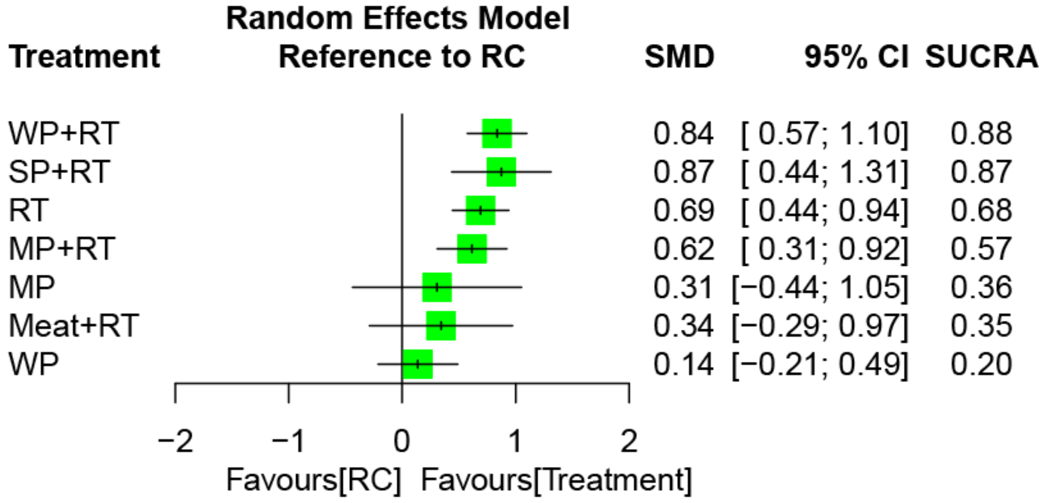

Figure S11. Forest plot of node-splitting results for chair rise

Comparison	Number of Studies	Direct Evidence	I2	SMD	95%CI
**MP+RT:RC**					
Direct estimate	1	0.24		0.40	[−0.23; 1.03]
Indirect estimate				0.68	[0.33; 1.04]
Network estimate				0.62	[0.31; 0.92]
**MP+RT:RT**					
Direct estimate	8	0.83	13%	−0.04	[−0.28; 0.20]
Indirect estimate				−0.24	[−0.78; 0.29]
Network estimate				−0.08	[−0.30; 0.15]
**MP+RT:WP+RT**					
Direct estimate	1	0.10		−0.25	[−1.07; 0.57]
Indirect estimate				−0.22	[−0.50; 0.06]
Network estimate				−0.22	[−0.48; 0.04]
**RT:RC**					
Direct estimate	7	0.72	83%	0.82	[0.53; 1.11]
Indirect estimate				0.35	[−0.12; 0.83]
Network estimate				0.69	[0.44; 0.94]
**SP+RT:RC**					
Direct estimate	1	0.50		0.92	[0.31; 1.54]
Indirect estimate				0.82	[0.20; 1.45]
Network estimate				0.87	[0.44; 1.31]
**WP:RC**					
Direct estimate	5	0.84	64%	0.02	[−0.36; 0.41]
Indirect estimate				0.75	[−0.13; 1.63]
Network estimate				0.14	[−0.21; 0.49]
**WP+RT:RC**					
Direct estimate	6	0.61	78%	0.98	[0.64; 1.32]
Indirect estimate				0.61	[0.20; 1.03]
Network estimate				0.84	[0.57; 1.10]
**RT:SP+RT**					
Direct estimate	2	0.80	0%	−0.17	[−0.63; 0.29]
Indirect estimate				−0.23	[−1.14; 0.68]
Network estimate				−0.18	[−0.59; 0.23]
**RT:WP**					
Direct estimate	5	0.74	93%	0.55	[0.16; 0.95]
Indirect estimate				0.56	[−0.10; 1.21]
Network estimate				0.55	[0.21; 0.89]
**RT:WP+RT**					
Direct estimate	18	0.94	42%	−0.12	[−0.29; 0.05]
Indirect estimate				−0.48	[−1.14; 0.18]
Network estimate				−0.14	[−0.31; 0.02]
**SP+RT:WP+RT**					
Direct estimate	1	0.35		0.00	[−0.71; 0.71]
Indirect estimate				0.06	[−0.46; 0.58]
Network estimate				0.04	[−0.38; 0.46]
**WP:WP+RT**					
Direct estimate	5	0.76	89%	−0.85	[−1.25; −0.46]
Indirect estimate				−0.21	[−0.91; 0.48]
Network estimate				−0.70	[−1.04; −0.36]

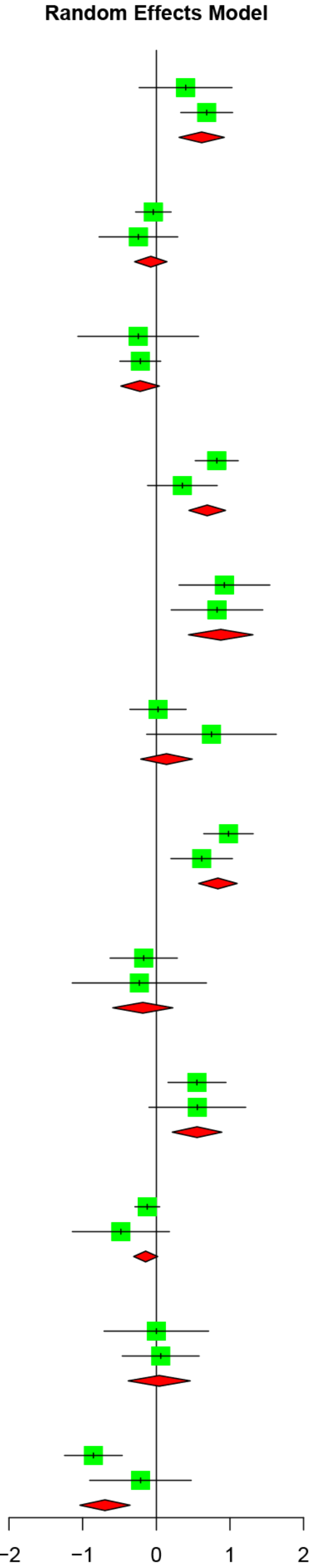

Supplementary Figure S12. Relative effects among treatment regimens for timed up-and-go task at each follow-up time frame

Follow up ≤3 months

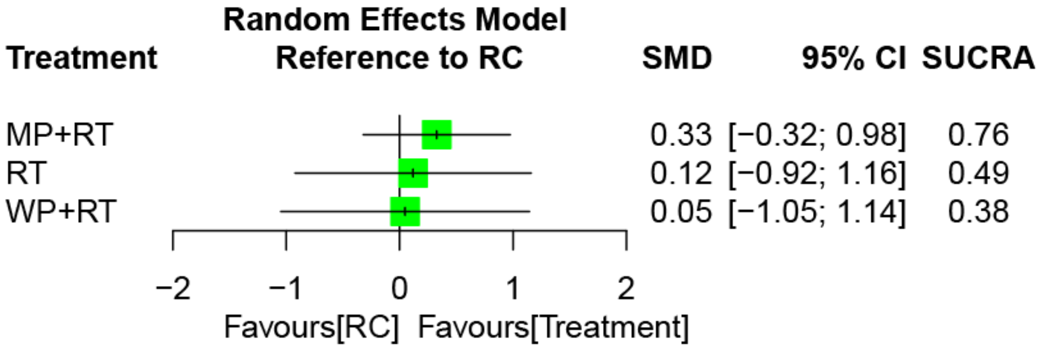

Follow up >3 months, ≤6 months

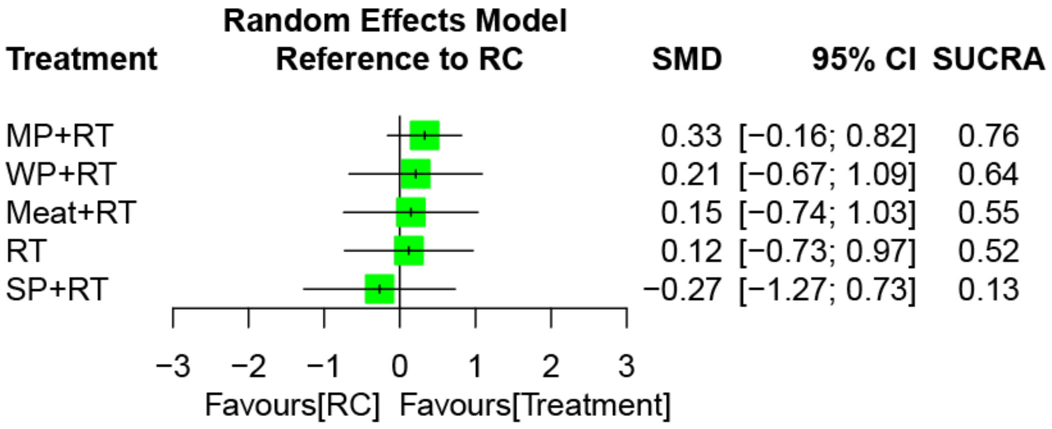

Overall follow up duration

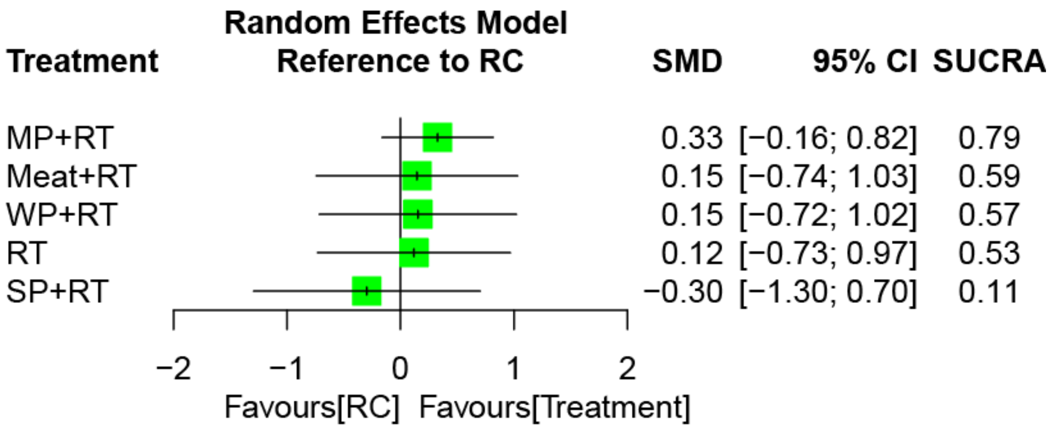

Figure S13. Forest plot of node-splitting results for timed up and go

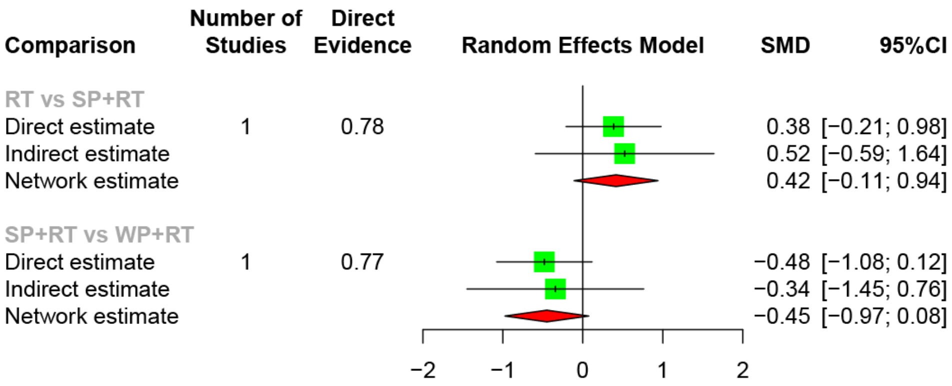

Supplementary Figure S14. Relative effects among treatment regimens for Short Physical Performance Battery at each follow-up time frame

Follow up ≤3 months

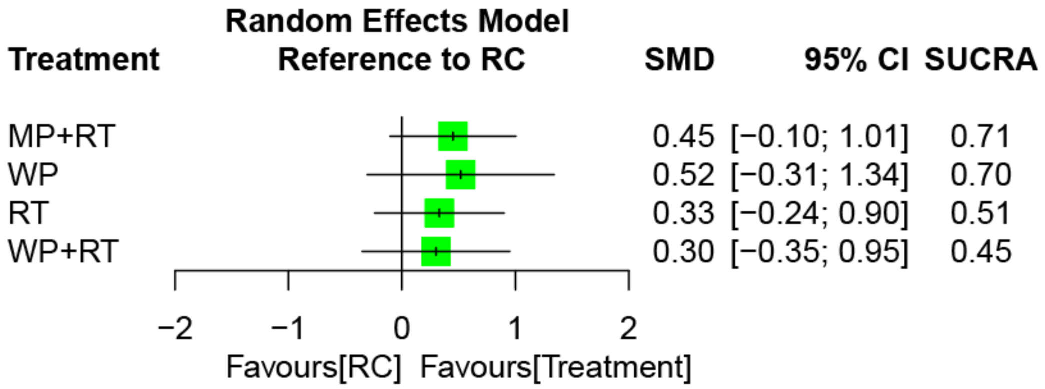

Follow up >3 months, ≤6 months

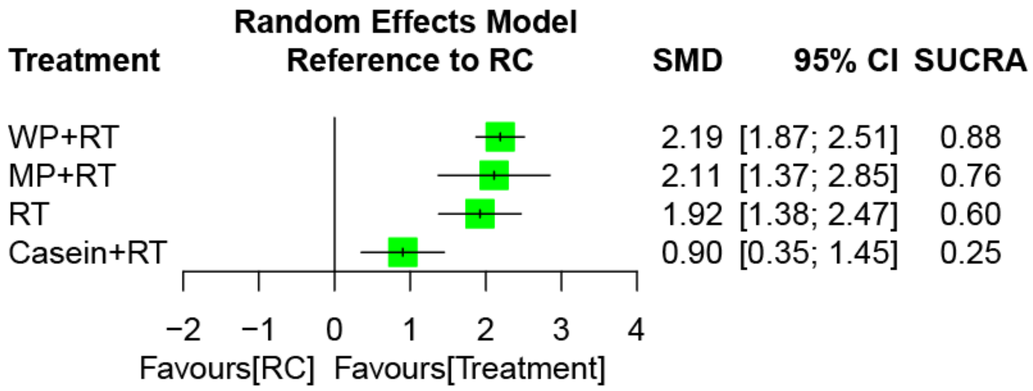

Overall follow up duration

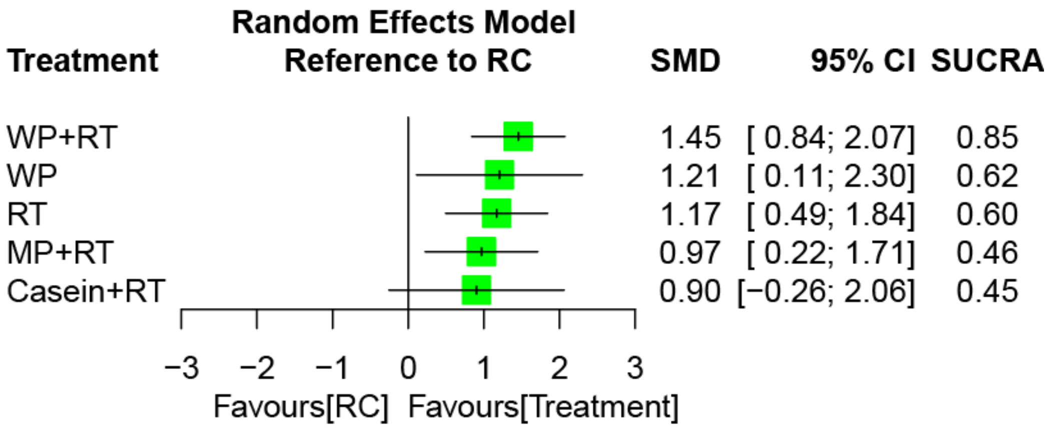

Figure S15. Forest plot of node-splitting results for SPPB

Comparison	Number of Studies	Direct Evidence	I2	SMD	95%CI
MP+RT:RC					
Direct estimate	1	0.43		0.33	[−0.83; 1.49]
Indirect estimate				1.45	[0.44; 2.47]
Network estimate				0.97	[0.20; 1.73]
MP+RT:RT					
Direct estimate	4	0.85	0%	−0.03	[−0.63; 0.57]
Indirect estimate				−1.15	[−2.57; 0.27]
Network estimate				−0.20	[−0.76; 0.35]
RT:RC					
Direct estimate	1	0.29		0.30	[−0.97; 1.58]
Indirect estimate				1.53	[0.70; 2.35]
Network estimate				1.17	[0.48; 1.86]
WP:RC					
Direct estimate	1	0.75		0.63	[−0.67; 1.92]
Indirect estimate				2.95	[0.70; 5.20]
Network estimate				1.21	[0.08; 2.33]
WP+RT:RC					
Direct estimate	3	0.79	87%	1.78	[1.07; 2.50]
Indirect estimate				0.22	[−1.15; 1.60]
Network estimate				1.45	[0.82; 2.09]
RT:WP					
Direct estimate	1	0.75		−0.32	[−1.59; 0.94]
Indirect estimate				0.84	[−1.37; 3.04]
Network estimate				−0.04	[−1.13; 1.06]
RT:WP+RT					
Direct estimate	5	0.88	0%	−0.03	[−0.59; 0.54]
Indirect estimate				−2.16	[−3.68; −0.64]
Network estimate				−0.28	[−0.81; 0.24]
WP:WP+RT					
Direct estimate	1	0.73		−0.01	[−1.28; 1.26]
Indirect estimate				−0.91	[−3.01; 1.19]
Network estimate				−0.25	[−1.33; 0.84]

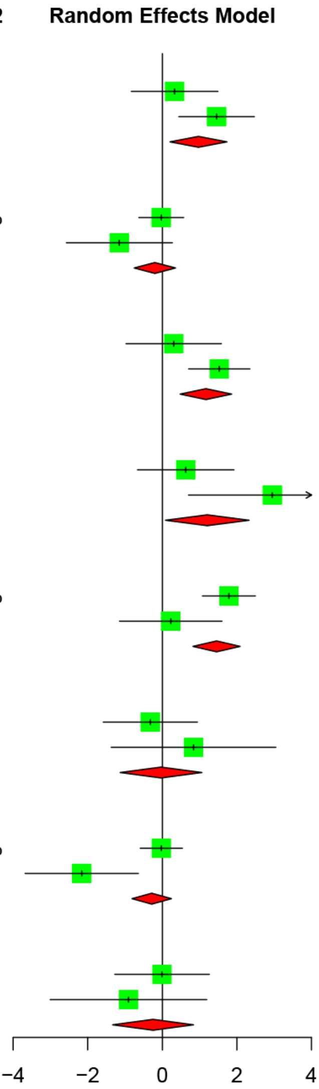

Supplementary figure S16

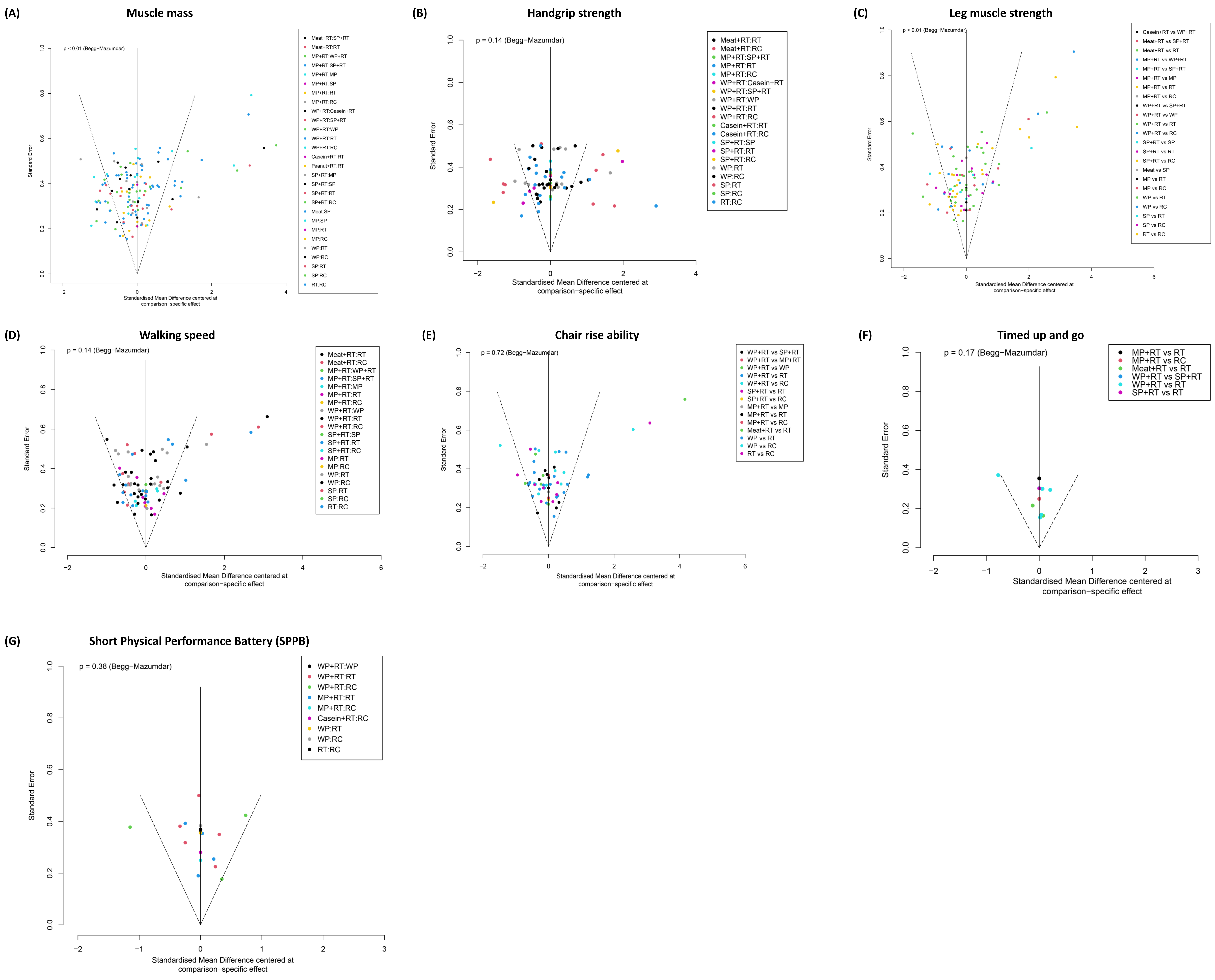

Supplement: Supplementary file 1 [file nutrients-16-00941-s001.zip › Supplementary figures S1-16.pdf]
