# Supplementary material for: Comparative Efficacy of Different Protein Supplements on Muscle Mass, Strength, and Physical Indices of Sarcopenia among Community-Dwelling, Hospitalized or Institutionalized Older Adults Undergoing Resistance Training: A Network Meta-Analysis of Randomized Controlled Trials
Source: Nutrients. 2024 Mar 25;16(7):941. doi: 10.3390/nu16070941 (PMC11013298; doi:10.3390/nu16070941)
Supplement: Supplementary file 1 [file nutrients-16-00941-s001.zip › Supplementary tables S1-18.pdf]

Table S1. Database search formulas

Data base	Search terms for query
**Pubmed**	
#1	(elder adults) OR order adults
#2	((sarcopenia) OR frailty) OR dynapenia)
#3	(((untrained) OR sedentary) OR hospitalized) OR Institutionalized
#4	#1 OR #2 OR #3
#5	(((progressive resistance training) OR resistance exercise) OR strength training) OR strengthening exercise
#6	(((protein supplement) OR protein supplementation) OR dietary protein) OR high-protein diet
#7	(Randomized controlled trial) OR Randomization
#8	((((#4) AND #5) AND #6) AND #7
**Physiotherapy Evidence Database (PEDro)**	
	Method: clinical trial
	Abstract & Title:
#1	order adults
#2	sarcopenia
#3	frailty
#4	resistance training
#5	strengthening exercise
#6	Strength training
#7	protein supplementation
#8	high-protein diet
#9	dietary protein
**China knowledge resource integrated database**	
#1	order adults
#2	resistance exercise training
#3	protein supplementation
#4	randomized controlled trial
#5	#1 AND #2 AND #3 AND #4

(continued)

Table S1. (continued)

Data base	Search terms for query
**Excerpta Medica dataBASE (EMBASE)**	
#1	order adults
#2	sarcopenia
#3	frailty
#4	dynapenia
#5	sedentary/untrained
#6	strengthening exercise
#7	resistance training
#8	strength training
#9	protein supplementation
#10	dietary protein
#11	#1 OR #2 OR #3 OR #4 OR #5
#12	#6 OR #7 OR #8
#13	#9 OR #10
#14	#14 AND #15 AND #16 AND ([systematic review]/lim OR [meta analysis]/lim OR [randomized controlled trial]/lim) AND [humans]/lim
**Cochrane Library Database**	
#1	order adults
#2	sarcopenia
#3	frailty
#4	sedentary/untrained
#5	resistance training
#6	strength exercise training
#7	strengthening exercise
#8	protein supplementation
#9	dietary protein
#10	randomized controlled trial
#11	#1 OR #2 OR #3 OR #4
#12	#5 OR #6 OR #7
#13	#8 AND #9
#14	#11 AND #12 AND #13
Google Scholar	
#1	allintitle: elderly OR older adults
#2	allintitle: untrained OR sarcopenia OR frailty
#3	allintitle: resistance training OR strengthening exercise
#4	allintitle: "protein supplementation" OR "dietary protein"
#5	allintitle: randomized controlled trial

Table S2. Summary of included study characteristics.

Study (year) (reference number)[a]	Country (area)	Population	Study arm	Mean age (years)	Mean BMI (kg/m²)	*N*	Sex		PS intervention			RT intervention				Outcome measures									Measured time point (week)
																Muscle mass/volume			Strength		Mobility				
							Female	Male	Frequency (intake day/week)	Intake amount (g/d)	Compliance (%)	% 1-RM	Frequency (session/ week)	Duration (week)	Compliance (%)	Wole body lean mass	Appendicular lean mass	CSA/ Muscle thickness	Handgrip strength	Leg strength	Walking speed	Chair rise	TUG	SPPB	
Aas 2019 [1]	Norway	Mobility-limited	MP+RT	86.6	26.1	11	7	4	7	34	NR	80–90	3	10	95.8		V	V		V	V	V		V	0, 10
	(Europe)	older adults	RC	82.6	26.3	11	8	3																	
Amasene 2019 [2];	Span	Post-hospitalized	WP+RT	82.9	27.4	21	12	9	2	20	NR	70	2	12	NR	V	V	V	V		V	V		V	0, 12
2021 [3]	(Europe)	sarcopenic older adults	PLA+RT	81.2	31.1	20	10	10																	
Arciero 2014 [4]	USA	Inactive middle-	WP+RT	47	29	22	17	5	4	60	75	RPE 7-9/10	4	16	85	V									0, 16
	(America)	aged adults	WP	50	28	18	11	7				(10-12 RM)													
Arnarson 2013 [5]	Iceland	Community-	WP+RT	73.3	28.1	83	94[b]	67[b]	3	20	NR	75-80	3	12	NR	V	V			V	V		V		0, 12
	(Europe)	dwelling elderly	PLA+RT	74.6	29.4	78																			
Bagheri 2022 [6]	Iran	Untrained older	WP+RT	67.3	21.2	14	0	14	7	18	>90	60–80	3	8	>90	V				V					0, 8
	(Asia)	adults	PLA+RT	68.4	21.9	14	0	14																	
Bijeh 2022 [7]	Iran	Untrained older	SP+RT	65.6[c]	NR	15	0	15	7	6.75	100	60	3	12	100	V			V	V					0, 12
	(Asia)	adults	RT			15	0	15							100										
			SP			15	0	15			100														
			RC			15	0	15																	
Candow 2006 [8]	Canada	Untrained older	WP+RT	64.9	28.3	19	0	19	3	25	NR	70	3	12	NR	V		V		V					0, 12
	(America)	adults	PLA+RT	64.6	29.1	10	0	10		(0.3 g/kg/d)															
Candow 2008 [9]	Canada	Untrained older	WP+Cre+RT	67.3	26.3	10	0	10	3	25	94	70	3	10	NR	V				V					0, 10
	(America)	adults	Cre+RT	65.5	28.1	13	0	13		(0.3 g/kg/d)	93														
			PLA+RT	64.1	26.1	12	0	12			95														
Chale 2013 [10]	USA	Mobility-limited	WP+RT	78	27	42	25	17	7	40	72.1	80	3	24	79.9	V		V		V	V	V		V	0, 24
	(America)	older adults	PLA+RT	77.3	26.9	38	22	16			82.3				80.2										
Chang 2019 [11]	China	Sarcopenic elderly	Casein+RT	73.5	19.8	28	6	22	7	20	NR	70	3	24	NR				V					V	0, 24
	(Asia)	people	RC	76.5	19.1	28	6	22																	
Chen 2017 [12]	China	Sarcopenic elderly	WP+RT	73.5	19.8	20	4	16	7	25	NR	70	3	24	NR				V					V	0, 24
	(Asia)	people	RC	76.5	19.1	20	4	16																	
Colonetti	Brazil	Institutionalized	WP+RT	73	30.4	8	1	7	3	30	NR	70	3	24	NR	V	V								0, 6, 24
2023 [13]	(America)	older adults	PLA+RT	75.4	29.9	10	3	7																	
			RC	74.1	26.8	8	5	3																	
Daly 2014 [14]	Australia	Institutionalized	Meat+RT	72.1	27.7	53	53	0	7	45	81	RPE 14-16/20	2	16	75	V		V		V		V	V		0, 16
	(Oceania)	older adults	CHO+RT	73.6	27.6	47	47	0		(1.3 g/kg/d)	100				72										

To be continued.

Table S2. Continued.

Study (year) (reference number)[a]	Country (area)	Population	Study arm	Mean age (years)	Mean BMI (kg/m²)	*N*	Sex		PS intervention			RT intervention				Outcome measures									Measured time point (week)
																Muscle mass/volume			Strength		Mobility				
							Female	Male	Frequency (intake day/week)	Intake amount (g/d)	Compliance (%)	% 1-RM	Frequency (session/ week)	Duration (week)	Compliance (%)	Wole body lean mass	Appendicular lean mass	CSA/ Muscle thickness	Handgrip strength	Leg strength	Walking speed	Chair rise	TUG	SPPB	
de Azevedo Bach	Brazil	Sedentary older	WP+RT	66.9	26.3	16	NR	NR	7	30	97.2	75-85 (6-8 RM)	2	12	100	V	V			V		V	V		0, 12
2022 [15]	(America)	adults	PLA+RT	65.8	25.4	15	NR	NR			97.1				99										
de Carvalho	Brazil	Dynapenic older	WP+RT	76.9	25.7	20	16	4	7	21	NR	80	3	12	98.5	V			V		V	V	V		0, 12
Bastone	(America)	adults	RT	77.6	26	20	12	8							97.2										
2020 [16]			WP	76.5	25.9	20	14	6																	
			RC	72.5	26.2	20	15	5																	
Deer 2019 [17]	USA	Hospitalized	WP+RT	80	26.1	20	14	6	7	40	75	NR (moderate	3	4	77	V	V				V	V		V	0, 4
	(America)	older adults	PLA+RT	77.6	27.4	21	14	7				intensity)													
			WP	80	28.9	20	14	6																	
			PLA	75.7	29	20	14	6																	
Deibert 2011 [18]	Germany	Untrained middle-	SP+RT	55.9	28.4	13	0	13	7	26.7	NR	NR (10 RM)	2	12	90	V				V					0, 12
	(Europe)	aged adults	RT	55.5	27.7	13	0	13			NR				90										
			RC	55.8	27.2	9	0	9																	
Duff 2014 [19]	Canada	Untrained older	MP+RT	61.8	26.9	19	12	7	7	38	97	NR (8-12 RM)	3	8	86	V		V		V					0, 8
	(America)	adults	WP+RT	57.5	25.9	21	13	8			88				84										
Dulac 2021 [20]	Canada	Sedentary older	WP+RT	68.3	26.7	21	0	21	7	30	NR	80	3	12	NR	V	V	V	V	V	V	V	V		0, 12
	(America)	adults	Casein+RT	69	26	20	0	20				(RPE 8-10/10)													
			PLA+RT	70.7	25.4	19	0	19																	
Eliot 2008 [21];	USA	Untrained middle-	WP+Cre+RT	57.2	28.7	11	0	11	3	35	100	80	3	14	100	V	V			V					0, 14
Bemben 2010 [22]	(America)	aged and older	WP+RT	58.2	28.6	11	0	11																	
		adults	Cre+RT	56.1	29.1	10	0	10																	
			PLA+RT	56.1	31.3	10	0	10																	
Fiatarone 1994 [23]	USA	Institutionalized	SP+RT	87.2	24.5	25	16	9	7	40	99	80	3	10	97	V		V		V	V				0, 10
	(America)	elderly	PLA+RT	86.2	24.9	25	16	9																	
			SP	85.7	25.4	24	17	7																	
			PLA	89.2	25.8	26	14	12																	
Formica 2020 [24]	Australia	Community-dwelling	Meat+RT	71.2	27.8	77	48	29	3	45	NR	RPE 5-8/10	3	24	77.9	V	V	V		V	V	V	V		0, 24
	(Oceania)	elderly	RT	70.3	27.9	77	48	29		(1.3 g/kg/d)					78.6										
Francis 2017 [25]	UK	Untrained free-	MP+RT	60.4	24.7	29	29	0	7	24	86	NR (8-15 RM)	3	12	NR	V	V			V	V	V			0, 12
	(Europe)	living older adults	MP	61.8	26.1	28	28	0		(0.33 g/kg/d)	82														

To be continued.

Table S2. Continued.

Study (year) (reference number)[a]	Country (area)	Population	Study arm	Mean age (years)	Mean BMI (kg/m²)	*N*	Sex		PS intervention			RT intervention				Outcome measures									Measured time point (week)
																Muscle mass/volume			Strength		Mobility				
							Female	Male	Frequency (intake day/week)	Intake amount (g/d)	Compliance (%)	% 1-RM	Frequency (session/ week)	Duration (week)	Compliance (%)	Wole body lean mass	Appendicular lean mass	CSA/ Muscle thickness	Handgrip strength	Leg strength	Walking speed	Chair rise	TUG	SPPB	
Gade 2019 [26]	Denmark	Hospitalized	MP+RT	85.3	25.1	73	51	22	7	27.5	62-87	NR (8-12 RM)	7	12	51	V	V		V		V	V			0, 1, 12
	(Europe)	older adults	PLA+RT	84.2	25.8	75	49	26			55-88														
Granic 2020 [27]	UK	Sarcopenic	Whole-MP+RT	72	24.9	10	4	6	2	17	97.1	>70	2	6	97.2	V	V		V		V	V		V	0, 6
	(Europe)	older adults	Skimmed-MP+RT	72.2	25.5	10	4	6			98.3				98.3										
			PLA+RT	70.8	27	10	4	6			95				95.8										
Griffen 2022 [28]	UK	Community-	WP+RT	68	26.6	9	0	9	7	50	96.1	80	2	12	98.2	V	V			V	V				0, 12
	(Europe)	dwelling older	PLA+RT	67	25.1	9	0	9			96.1				98.2										
		adults	WP	66	25	9	0	9			96.8														
			PLA	67	25.1	9	0	9			94.1														
Gronstedt 2020 [29]	Sweden (Europe)	Institutionalized elderly	MP+RT	85.8	25.3	52	34	18	7	36	64	NR	7	12	44	V					V	V			0, 12
			RT	85.9	25.3	50	29	21							44										
Hamarsland	Norway	Untrained	MP+RT	74.3	NR	15	6	9	7	40	99	NR (6-12 RM)	3	11	98.5	V	V	V		V		V			0, 11
2019 [30]	(Europe)	older adults	WP+RT	72.9	NR	15	6	9			99				100										
Haß 2022 [31]	Germany	Community-	WP+Omega-3+RT	70.4	27.8	21	11	10	7	27	96	NR	3	8	NR	V			V	V	V	V			0, 8
	(Europe)	dwelling older	WP+RT	71.5	28.2	20	11	9			98														
		adults	RT	69.9	26.9	20	10	10																	
Haub 2002 [32];	USA	Community-dwelling	Meat+RT	63	28.1	10	0	10	7	50	NR	80	2	12	94-100	V		V		V					0, 12
Haub 2005 [33]	(America)	older adults	SP+RT	67	28.3	11	0	11		(0.33 g/kg/d)															
He 2022 [34]	China	Sarcopenic older	WP+RT	82.4	19.4	75	0	75	7	25	NR	NR	7	4	NR				V						0, 4
	(Asia)	adults	RC	82.4	19.4	75	0	75																	
Holm 2008 [35]	Denmark	Postmenopausal	WP+RT	55	24	13	13	0	2-3	10	NR	NR (8 RM)	2-3	24	81-91.7	V		V		V					0, 12, 24
	(Europe)	women	PLA+RT	55	27	16	16	0							81-91.7										
Holwerda	Netherlands	Community-dwelling	WP+RT	69	25.5	21	0	21	7	21	99.6	80	3	12	95	V	V			V	V	V		V	0, 12
2018 [36]	(Europe)	older adults	PLA+RT	71	25.1	20	0	20																	
Kang 2020 [37]	Korea	Untrained older	MP+RT	61.2	23.7	60	41	19	7	40	>80	light	7	12	NR	V	V	V	V	V				V	0, 12
	(Asia)	adults	PLA+RT	58.4	23.6	60	46	14			>80														
Karelis 2015 [38]	Canada	Sedentary non-frail	WP+RT	69.9	24.9	34	26	8	7	20	97	80	3	19	90	V	V			V					0, 19
	(America)	elderly	Casein+RT	71	25.4	33	26	7			96				91										
Kemmler	Germany	Elderly with	WP+RT	77.8	25	21	0	21	7	80	NR (high)	80	2-3	52	95	V			V	V	V				0, 52
2020a [39]; 2020b [40]	(Europe)	osteosarcopenia	WP	79.2	24.5	22	0	22		(0.33 g/kg/d)															

To be continued.

Table S2. Continued.

Study (year) (reference number)[a]	Country (area)	Population	Study arm	Mean age (years)	Mean BMI (kg/m²)	*N*	Sex		PS intervention			RT intervention				Outcome measures									Measured time point (week)
																Muscle mass/volume			Strength		Mobility				
							Female	Male	Frequency (intake day/week)	Intake amount (g/d)	Compliance (%)	% 1-RM	Frequency (session/ week)	Duration (week)	Compliance (%)	Wole body lean mass	Appendicular lean mass	CSA/ Muscle thickness	Handgrip strength	Leg strength	Walking speed	Chair rise	TUG	SPPB	
Krause 2019 [41]	Ireland (Europe)	Sedentary, medically-stable older adults	WP+RT	63.9	25.4	11	7	4	7	24 (0.33 g/kg/d)	NR	NR (8-15 RM)	3	12	NR	V			V		V	V			0, 12
			PLA+RT	63.9	25	10	5	5																	
			WP	62.3	27.2	7	3	4																	
			PLA	63.8	26.3	10	5	5																	
Kukuljan 2009a [42]; 2009b [43]	Australia (Oceania)	Untrained middle-aged and older adults	MP+RT	61.7	27.4	45	0	45	7	13	92	80-85	3	52	69	V		V		V	V				0, 52
			RT	60.7	28.1	46	0	46							65										
			MP	61.7	27.7	45	0	45			90														
			RC	59.9	26.7	44	0	44																	
Kwon 2015 [44]	Janan (Asia)	Pre-frail older adults	Meat+RT	76.5	NR	26	0	26	7	20-22	NR	NR	1	12	NR				V		V				0, 12
			RT	77.0	NR	25	0	25																	
			RC	76.9	NR	28	0	28																	
Lamb 2020 [45]	USA (America)	Untrained older adults	Peanut+RT	60.0	27.8	20	8	12	7	35	NR	RPE 7-9/10 (10-12 RM)	2	10	NR	V		V		V					0, 6
			RT	58.0	29.7	19	9	10																	
Leenders 2013 [46]	Netherlands (Europe)	Untrained elderly	MP+RT	71.5	25.8	27	12	15	7	15	NR	75-80	3	24	90	V	V	V	V	V		V			0, 12, 24
			PLA+RT	69.5	25.9	26	12	14																	
Liao 2021 [47]	Taiwan (Asia)	Older adults with knee osteoarthritis	WP+RT	68.6	28.1	36	36	0	2	14	100	RPE 13-15/20	2	12	84.3	V			V		V				0, 12
			RT	69.8	27.4	36	36	0							82.6										
Maesta 2007 [48]	Brazil (America)	Postmenopausal women	SP+RT	57.6	27.8	14	14	0	NR	25	NR	60-80	3	16	NR	V									0, 16
			PLA+RT	60.7	27.7	11	11	0		12.2															
			SP	61.3	27.2	10	10	0																	
			PLA	57.9	26.6	11	11	0																	
Maltais 2016 [49]	Canada (America)	Sarcopenic elderly men	MP+RT	68	25.8	10	0	10	3	13.5	>90	80	3	16	>90	V	V			V	V	V	V		0, 16
			SP+RT	64	27	10	0	10		12	>90				>90										
			PLA+RT	64	25.9	10	0	10			>90				>90										
McKenna 2021 [50]	USA (America)	Untrained middle-aged and older adults	Meat+RT	49	27.6	28	14	14	7	60	97	75	3	10	89	V	V		V	V	V				0, 10
			RT	50	27.5	22	13	9			92				87										
Mertz 2021 [51]	Denmark (Europe)	Community-dwelling older adults	WP+RT	70.4	25.8	72	36	36	7	40	88	NR (6 RM)	3	52	72-89	V		V		V	V				0, 52
			WP	70.3	25.2	50	22	28			88														
			PLA	69.6	26	36	18	18			95														
Miller 2021 [52]	Australia (Oceania)	Overweight or obese older adults with DM	WP+RT	61.1	29.6	98	36	62	7	40	78.6	RPE 12-15/20	3	24	67.5	V	V	V				V	V		0, 24
			RT	62	31.1	100	36	64							57.9										

To be continued.

Table S2. Continued.

Study (year) (reference number)[a]	Country (area)	Population	Study arm	Mean age (years)	Mean BMI (kg/m²)	*N*	Sex		PS intervention			RT intervention				Outcome measures									Measured time point (week)
																Muscle mass/volume			Strength		Mobility				
							Female	Male	Frequency (intake day/week)	Intake amount (g/d)	Compliance (%)	% 1-RM	Frequency (session/ week)	Duration (week)	Compliance (%)	Wole body lean mass	Appendicular lean mass	CSA/ Muscle thickness	Handgrip strength	Leg strength	Walking speed	Chair rise	TUG	SPPB	
Mitchell 2018 [53]	New Zealand (Oceania)	Middle-aged and older adults	MP+RT	51.5	27.5	15	0	15	3	20	NR	80	3	2	NR			V		V					0, 2
			PLA+RT	48.5	28.3	15	0	15																	
Molnar 2016 [54]	Hungary (Europe)	Institutionalized elderly	WP+RT	66.6	22.6	17	10	7	7	40	NR	NR	2	12	NR	V				V				V	0, 12
			RT	66.4	28.1	17	12	5																	
Mori 2018 [55]	Janan (Asia)	Untrained community-dwelling elderly	WP+RT	70.6	22.1	25	25	0	2	11	90.1	50-70	2	24	90.1	V	V		V	V	V				0, 24
			RT	70.6	22.9	25	25	0							86.6										
			WP	70.6	22.3	25	25	0	7	11	86.5														
Mori 2022 [56]	Janan (Asia)	Older adults with sarcopenia	WP+RT	77.7	20.3	23	20	3	2	11	73.2	50-70	2	24	73.2				V	V	V				0, 24, 36, 48
			RT	77.6	20.3	23	19	4							72.4										
			WP	77.8	20.1	24	20	4	7	11	73														
Nabuco 2018 [57]; 2019a [58]	Brazil (America)	Pre-conditioned older adults	WP+RT	66.9	25.9	47	47	0	3	27	NR	NR (8-12 RM)	3	12	NR	V	V			V	V	V			0, 12
			PLA+RT	66.5	23.8	23	23	0																	
Nabuco 2019b [59]	Brazil (America)	Older adults with sarcopenic obesity	WP+RT	68	26.4	13	13	0	3	35	NR	NR (12 RM)	3	12	NR	V	V								0, 12
			PLA+RT	70.1	27.4	13	13	0																	
Nabuco 2019c [60]	Brazil (America)	Community-dwelling older adults	WP+RT	69.2	27.4	15	15	0	3	35	NR	60-70 (12 RM)	3	12	NR	V				V					0, 12
			PLA+RT	68.4	26.6	15	15	0																	
Nilsson 2020 [61]	Canada (America)	Sedentary elderly	MP+RT	77.4	29.3	16	0	16	7	24	89.3	NR (10-15 RM)	3	12	84.1	V	V		V	V	V	V	V	V	0, 12
			PLA+RT	74.4	27.4	16	0	16			95.4				89.1										
Oesen 2015 [62]	Australia (Oceania)	Institutionalized elder	WP+RT	81.8	29.8	36	31	5	7	40	NR	RPE <7/10	2	24	71				V	V	V	V			0, 12, 24
			RT	83	28.9	41	37	4							71										
			RC	83.4	28.9	40	35	5																	
Orsatti 2018 [63]	Brazil (America)	Postmenopausal women	SP+RT	56.8	27.5	16	16	0	7	25	NR	60-80 (8-12 RM)	3	16	100	V				V					0, 16
			PLA+RT	58.8	27.3	16	16	0							100										
Roschel 2021 [64]	Brazil (America)	Frail elderly	WP+RT	72	26.7	22	22	0	7	30	NR	70	2	16	NR	V	V	V	V	V		V	V		0, 16
			SP+RT	72	28.6	22	22	0																	
			PLA+RT	73	28.8	22	22	0																	
Seino 2018 [65]	Janan (Asia)	Sedentary older adults	MP+RT	73.4	22.9	40	34	6	7	10.5	>90	RPE 5-7/10 (20 RM)	2	12	95.3	V	V		V	V	V	V	V		0, 12
			RT	73.7	22.9	40	33	7							94.6										
Shahar 2013 [66]	Malaysia (Asia)	Elderly with sarcopenia	SP+RT	65.2	26.5	15	18[b]	47[b]	7	20 (men); 40 (women)	86.7	NR	2	12	86.7	V	V								0, 6, 12
			RT	69.7	23.7	19																			
			SP	65.9	24.3	15																			
			RC	67.3	26.4	16																			

To be continued.

Table S2. Continued.

Study (year) (reference number)[a]	Country (area)	Population	Study arm	Mean age (years)	Mean BMI (kg/m²)	*N*	Sex		PS intervention			RT intervention				Outcome measures									Measured time point (week)
																Muscle mass/volume			Strength		Mobility				
							Female	Male	Frequency (intake day/week)	Intake amount (g/d)	Compliance (%)	% 1-RM	Frequency (session/ week)	Duration (week)	Compliance (%)	Wole body lean mass	Appendicular lean mass	CSA/ Muscle thickness	Handgrip strength	Leg strength	Walking speed	Chair rise	TUG	SPPB	
Shenoy 2013 [67]	India (Asia)	Postmenopausal women	SP+RT	54.1	28.9	20	20	0	7	40	NR	80	4	12	NR					V					0, 12
			SP	54.6	31	20	20	0																	
			RC	54.1	30	20	20	0																	
Soares 2023 [68]	Brazil (America)	Older adults with type 2 DM	WP+RT	68.1	29.3	14	0	14	2	20	NR	70 (RPE 7-8/10)	2	12	NR	V			V	V					0, 12
			PLA+RT	68.9	26.8	14	0	14																	
Sugihara Junior 2018 [69]	Brazil (America)	Pre-conditioned older adults	WP+RT	67.4	25.6	15	15	0	3	35	NR	NR (8-12 RM)	3	12	>85	V	V			V					0, 12
			PLA+RT	67.8	25.4	16	16	0																	
Tang 2020 [70]	China (Asia)	Older adults with sarcopenia	WP+RT	76.6	19.3	100	0	100	7	25	NR	NR (20-30 RM)	6	24	NR				V					V	0, 24
			RC	77	19.5	100	0	100																	
Thomson 2016 [71]	Australia (Oceania)	Overweight older adults	MP+RT	61.3	27.7	54	29	25	7	27	97	NR (8-12 RM)	3	12	91.4	V			V	V	V				0, 12
			SP+RT	61.7	27.5	64	35	29		27	98.1				93.2										
			PLA+RT	61.5	27.6	61	34	27			98.4				92.9										
Tieland 2012 [72]; Dirks 2017 [73]	Netherlands (Europe)	Frail elderly people	MP+RT	78	28.7	31	20	11	7	30	≥98%	70	2	24	≥98%	V	V	V	V	V	V	V		V	0, 12, 24
			PLA+RT	79	28.2	31	21	10																	
Trevisan 2010 [74]	Brazil (America)	Postmenopausal women, untrained (low muscle mass, overweight)	SP+RT	58	28	15	15	0	7	25	NR	60-80	3	12	NR	V									0, 12
			PLA+RT	57	28	15	15	0																	
			SP	60	29	15	15	0																	
			PLA	60	27	15	15	0																	
Unterberger 2022 [75]	Australia (Oceania)	Untrained older adults	SP+RT	73.2	26.2	48	28	20	2	32	NR	RPE 6-7/10 (8-12 RM)	2	10	NR	V	V		V	V	V	V	V		0, 10
			PLA+RT	72.4	26.3	41	21	20																	
			RC	73	26	47	24	23																	
Verdijk 2009 [76]	Netherlands (Europe)	Free-living elderly people	Casein+RT	72	26.5	13	0	13	3	20	NR	75-80	3	12	NR	V		V							0, 12
			PLA+RT	72	27.4	13	0	13																	
Verreijen 2015 [77]	Netherlands (Europe)	Obese older adults	WP+RT	63.7	32.7	30	16	14	7	40	91	NR (12 RM)	3	13	72	V	V		V		V	V			0, 13
			PLA+RT	63	33.3	30	16	14			97				88										
Vijayakumaran 2023 [78]	Malaysia (Asia)	Sarcopenic older adults	WP+RT	66.6	26.4	8	8	0	7	15	90	75-80	3	12	90		V	V	V			V		V	0, 12
			RT	65.5	24.5	8	8	0																	
Vikberg 2019 [79]	Sweden (Europe)	Old individuals with pre-sarcopenia	MP+RT	70.9	22.7	36	20	16	7	20-30	84	RPE 6-7/10 (8-10 RM)	7	10	91	V	V		V		V	V	V	V	0, 10
			RC	70	23.3	34	18	16																	
Villanueva 2014 [80]	USA (America)	Untrained older adults	WP+RT	68.7	25.3	7	0	7	7	35	100	70	3	12	94	V				V	V				0, 6, 12
			RT	68.7	27.1	7	0	7																	
			RC	67.1	26.8	8	0	8																	

To be continued.

Table S2. Continued.

Study (year) (reference number)[a]	Country (area)	Population	Study arm	Mean age (years)	Mean BMI (kg/m^2)	*N*	Sex		PS intervention			RT intervention				Outcome measures									Measured time point (week)
																Muscle mass/volume			Strength		Mobility				
							Female	Male	Frequency (intake day/week)	Intake amount (g/d)	Compliance (%)	% 1-RM	Frequency (session/ week)	Duration (week)	Compliance (%)	Wole body lean mass	Appendicular lean mass	CSA/ Muscle thickness	Handgrip strength	Leg strength	Walking speed	Chair rise	TUG	SPPB	
Weisgarber 2015 [81]	Canada (America)	Postmenopausal women	WP+RT	57[c]	28.3[c]	12	12	0	4	40	100	30	4	10	NR		V	V		V					0, 10
			PLA+RT			12	12	0																	
Yamada 2019 [82]	Janan (Asia)	Older adults with sarcopenia or dynapenia	WP+RT	84.9	21.3	28	20	8	7	10	97.6	NR (20 RM)	2	12	88		V		V	V	V	V			0, 12
			RT	84.7	22.6	28	18	10							81										
			WP	83.2	22.6	28	20	8			100														
			RC	83.9	21.2	28	15	13																	
Zdzieblik 2021 [83]	Austria (Europe)	Untrained overweight middle-aged adults	WP+RT	49.6	30.2	36	0	36	7	15	NR	85	3	12	NR	V	V			V					0, 12
			PLA+RT	47.4	29.9	31	0	31																	
Zhao 2022 [84]	China (Asia)	Sarcopenic older adults	WP+RT	61.9	19.9	20	12	8	7	24	NR	NR (8 RM)	7	12	NR	V			V		V				0, 4, 8, 12
			WP	64.9	18.6	20	12	8																	
			RC	62.9	19.8	20	12	8																	
Zhu 2019 [85]	China (Asia)	Sarcopenic older adults	SP+RT	74.8	18.8	36	29	7	3	17.2	86.3	40	3	12	92.8		V		V	V	V	V			0, 12, 24
			RT	74.5	18.8	40	29	11							95.3										
			RC	72.2	18.9	37	29	8																	

[a]The number denotes the citation of the indicated reference at the end of table.
[b]Data is represented as total number of study sample.
[c]Data is represented as mean value of study sample.
1-RM, one-repetition maximum; BMI, body mass index; Cre, creatine; CSA, cross-section area; DM, diabetes mellitus; MP, milk protein; NR, not report; PLA, placebo; RPE, rate of perceived exertion; PS, protein supplementation; RC, regular care; RT, resistance training; SP, soy protein; SPPB, Short Physical Performance Battery; TUG, timed up and go; UK, United Kingdom; USA, United States of America; WP, whey protein.

References of supplementary Table S2

1. Aas SN, Seynnes O, Benestad HB, Raastad T: Strength training and protein supplementation improve muscle mass, strength, and function in mobility-limited older adults: a randomized controlled trial. *Aging Clin Exp Res* **2019**.
2. Amasene M, Besga A, Echeverria I, Urquiza M, Ruiz JR, Rodriguez-Larrad A, Aldamiz M, Anaut P, Irazusta J, Labayen I: Effects of Leucine-Enriched Whey Protein Supplementation on Physical Function in Post-Hospitalized Older Adults Participating in 12-Weeks of Resistance Training Program: A Randomized Controlled Trial. *Nutrients* **2019**, *11*.
3. Amasene M, Cadenas-Sanchez C, Echeverria I, Sanz B, Alonso C, Tobalina I, Irazusta J, Labayen I, Besga A: Effects of Resistance Training Intervention along with Leucine-Enriched Whey Protein Supplementation on Sarcopenia and Frailty in Post-Hospitalized Older Adults: Preliminary Findings of a Randomized Controlled Trial. *J Clin Med* **2021**, *11*.
4. Arciero PJ, Baur D, Connelly S, Ormsbee MJ: Timed-daily ingestion of whey protein and exercise training reduces visceral adipose tissue mass and improves insulin resistance: the PRISE study. *Journal of applied physiology (Bethesda, Md : 1985)* **2014**, *117*:1-10.
5. Arnarson A, Gudny Geirsdottir O, Ramel A, Briem K, Jonsson PV, Thorsdottir I: Effects of whey proteins and carbohydrates on the efficacy of resistance training in elderly people: Double blind, randomised controlled trial. *Eur J Clin Nutr* **2013**, *67*:821-826.
6. Bagheri R, Hooshmand Moghadam B, Candow DG, Elliott BT, Wong A, Ashtary-Larky D, Forbes SC, Rashidlamir A: Effects of Icelandic yogurt consumption and resistance training in healthy untrained older males. *Br J Nutr* **2022**, *127*:1334-1342.

7. Bijeh N, Mohammadnia-Ahmadi M, Hooshamnd-Moghadam B, Eskandari M, Golestani F: Effects of Soy Milk in Conjunction With Resistance Training on Physical Performance and Skeletal Muscle Regulatory Markers in Older Men. *Biol Res Nurs* **2022**, *24***:**294-307.
8. Candow DG, Chilibeck PD, Facci M, Abeysekara S, Zello GA: Protein supplementation before and after resistance training in older men. *Eur J Appl Physiol* **2006**, *97***:**548-556.
9. Candow DG, Little JP, Chilibeck PD, Abeysekara S, Zello GA, Kazachkov M, Cornish SM, Yu PH: Low-dose creatine combined with protein during resistance training in older men. *Med Sci Sports Exerc* **2008**, *40***:**1645-1652.
10. Chale A, Cloutier GJ, Hau C, Phillips EM, Dallal GE, Fielding RA: Efficacy of whey protein supplementation on resistance exercise-induced changes in lean mass, muscle strength, and physical function in mobility-limited older adults. *The journals of gerontology Series A, Biological sciences and medical sciences* **2013**, *68***:**682-690.
11. Chang YM: Effects of resistance training combined with nutritional intervention on alleviating sarcopenia in the elderly. *Electron J Practical Clin Nurs Sci* **2019**, *4***:**48-50.
12. Chen S, Sheng YL, Qi T, Sheng XM, Jin XW: Effects of fortified nutrition combining with resistance exercise on physical function and activity of daily living for elderly patients with sarcopenia. *J Nurs Sci* **2017**, *32***:**8-10.
13. Colonetti T, Grande AJ, da Rocha FR, Ronconi Dondossola E, Tuon L, Gomes Batista Teles H, Minotto Bom B, Colonetti L, da Rosa MI: Whey protein and vitamin D supplementation in institutionalized older adults: A randomized trial. *Nutr Health* **2023**, *29***:**129-138.
14. Daly RM, O'Connell SL, Mundell NL, Grimes CA, Dunstan DW, Nowson CA: Protein-enriched diet, with the use of lean red meat, combined with progressive resistance training enhances lean tissue mass and muscle strength and reduces circulating IL-6 concentrations in elderly women: a cluster randomized controlled trial. *Am J Clin Nutr* **2014**, *99***:**899-910.
15. de Azevedo Bach S, Radaelli R, Beck Schemes M, Neske R, Garbelotto C, Roschel H, Silveira Pinto R, Dornelles Schneider C: Can supplemental protein to low-protein containing meals superimpose on resistance-training muscle adaptations in older adults? A randomized clinical trial. *Exp Gerontol* **2022**, *162***:**111760.
16. de Carvalho Bastone A, Nobre LN, de Souza Moreira B, Rosa IF, Ferreira GB, Santos DDL, Monteiro N, Alves MD, Gandra RA, de Lira EM: Independent and combined effect of home-based progressive resistance training and nutritional supplementation on muscle strength, muscle mass and physical function in dynapenic older adults with low protein intake: A randomized controlled trial. *Arch Gerontol Geriatr* **2020**, *89***:**104098.
17. Deer RR, Dickinson JM, Baillargeon J, Fisher SR, Raji M, Volpi E: A Phase I Randomized Clinical Trial of Evidence-Based, Pragmatic Interventions to Improve Functional Recovery After Hospitalization in Geriatric Patients. *J Gerontol A Biol Sci Med Sci* **2019**, *74***:**1628-1636.
18. Deibert P, Solleder F, König D, Vitolins MZ, Dickhuth HH, Gollhofer A, Berg A: Soy protein based supplementation supports metabolic effects of resistance training in previously untrained middle aged males. *Aging Male* **2011**, *14***:**273-279.
19. Duff WR, Chilibeck PD, Rooke JJ, Kaviani M, Krentz JR, Haines DM: The effect of bovine colostrum supplementation in older adults during resistance training. *Int J Sport Nutr Exerc Metab* **2014**, *24***:**276-285.
20. Dulac MC, Pion CH, Lemieux FC, Pinheiro Carvalho L, El Hajj Boutros G, Bélanger M, Gaudreau P, Chevalier S, Morais JA, Noirez P, et al: Effects of slow- v. fast-digested protein supplementation combined with mixed power training on muscle function and functional capacities in older men. *Br J Nutr* **2021**, *125***:**1017-1033.
21. Eliot KA, Knehans AW, Bemben DA, Witten MS, Carter J, Bemben MG: The effects of creatine and whey protein supplementation on body composition in men aged 48 to 72 years during resistance training. *Journal of Nutrition, Health and Aging* **2008**, *12***:**208-212.
22. Bemben MG, Witten MS, Carter JM, Eliot KA, Knehans AW, Bemben DA: The effects of supplementation with creatine and protein on muscle strength following a traditional resistance training program in middle-aged and older men. *J Nutr Health Aging* **2010**, *14***:**155-159.
23. Fiatarone MA, O'Neill EF, Ryan ND, Clements KM, Solares GR, Nelson ME, Roberts SB, Kehayias JJ, Lipsitz LA, Evans WJ: Exercise Training and Nutritional Supplementation for Physical Frailty in Very Elderly People. *New England J Med* **1994**, *330***:**1769-1775.
24. Formica MB, Gianoudis J, Nowson CA, O'Connell SL, Milte C, Ellis KA, Daly RM: Effect of lean red meat combined with a multicomponent exercise program on muscle and cognitive function in older adults: a 6-month randomized controlled trial. *Am J Clin Nutr* **2020**, *112***:**113-128.
25. Francis P, Mc Cormack W, Toomey C, Norton C, Saunders J, Kerin E, Lyons M, Jakeman P: Twelve weeks' progressive resistance training combined with protein supplementation beyond habitual intakes increases upper leg lean tissue mass, muscle strength and extended gait speed in healthy older women. *Biogerontology* **2017**, *18***:**881-891.
26. Gade J, Beck AM, Andersen HE, Christensen B, Rønholt F, Klausen TW, Vinther A, Astrup A: Protein supplementation combined with low-intensity resistance training in geriatric medical patients during and after hospitalisation: a randomised, double-blind, multicentre trial. *Br J Nutr* **2019**, *122***:**1006-1020.
27. Granic A, Hurst C, Dismore L, Stevenson E, Sayer AA, Aspray T: Feasibility and acceptability of a milk and resistance exercise intervention to improve muscle function in community-dwelling older adults (MIlkMAN): Pilot study. *PLoS One* **2020**, *15***:**e0235952.
28. Griffen C, Duncan M, Hattersley J, Weickert MO, Dallaway A, Renshaw D: Effects of resistance exercise and whey protein supplementation on skeletal muscle strength, mass, physical function, and hormonal and inflammatory biomarkers in healthy active older men: a randomised, double-blind, placebo-controlled trial. *Exp Gerontol* **2022**, *158***:**111651.

29. Gronstedt H, Vikstrom S, Cederholm T, Franzen E, Luiking YC, Seiger A, Wimo A, Faxen-Irving G, Bostrom AM: Effect of Sit-to-Stand Exercises Combined With Protein-Rich Oral Supplementation in Older Persons: The Older Person's Exercise and Nutrition Study. *J Am Med Dir Assoc* **2020**, *21*:1229-1237.
30. Hamarsland H, Johansen MK, Seeberg F, Brochmann M, Garthe I, Benestad HB, Raastad T: Native Whey Induces Similar Adaptation to Strength Training as Milk, despite Higher Levels of Leucine, in Elderly Individuals. *Nutrients* **2019**, *11*.
31. Haß U, Kochlik B, Herpich C, Rudloff S, Norman K: Effects of an Omega-3 Supplemented, High-Protein Diet in Combination with Vibration and Resistance Exercise on Muscle Power and Inflammation in Old Adults: A Pilot Randomized Controlled Trial. *Nutrients* **2022**, *14*.
32. Haub MD, Wells AM, Tarnopolsky MA, Campbell WW: Effect of protein source on resistive-training-induced changes in body composition and muscle size in older men. *Am J Clin Nutr* **2002**, *76*:511-517.
33. Haub MD, Wells AM, Campbell WW: Beef and soy-based food supplements differentially affect serum lipoprotein-lipid profiles because of changes in carbohydrate intake and novel nutrient intake ratios in older men who resistive-train. *Metabolism* **2005**, *54*:769-774.
34. He LN, Li DM: Observation on the Clinical Effect of Dietary Nutrition Intervention Combined with Anti-resistance Training in Elderly Male Patients with Sarcopenia. *Smart Healthcare* **2022**, *8*:44-47.
35. Holm L, Olesen JL, Matsumoto K, Doi T, Mizuno M, Alsted TJ, Mackey AL, Schwarz P, Kjaer M: Protein-containing nutrient supplementation following strength training enhances the effect on muscle mass, strength, and bone formation in postmenopausal women. *J Appl Physiol (1985)* **2008**, *105*:274-281.
36. Holwerda AM, Overkamp M, Paulussen KJM, Smeets JSJ, van Kranenburg J, Backx EMP, Gijsen AP, Goessens JPB, Verdijk LB, van Loon LJC: Protein Supplementation after Exercise and before Sleep Does Not Further Augment Muscle Mass and Strength Gains during Resistance Exercise Training in Active Older Men. *J Nutr* **2018**, *148*:1723-1732.
37. Kang Y, Kim N, Choi YJ, Lee Y, Yun J, Park SJ, Park HS, Chung YS, Park YK: Leucine-Enriched Protein Supplementation Increases Lean Body Mass in Healthy Korean Adults Aged 50 Years and Older: A Randomized, Double-Blind, Placebo-Controlled Trial. *Nutrients* **2020**, *12*.
38. Karelis AD, Messier V, Suppere C, Briand P, Rabasa-Lhoret R: Effect of cysteine-rich whey protein (immunocal(R)) supplementation in combination with resistance training on muscle strength and lean body mass in non-frail elderly subjects: a randomized, double-blind controlled study. *J Nutr Health Aging* **2015**, *19*:531-536.
39. Kemmler W, Kohl M, Fröhlich M, Jakob F, Engelke K, von Stengel S, Schoene D: Effects of High-Intensity Resistance Training on Osteopenia and Sarcopenia Parameters in Older Men with Osteosarcopenia-One-Year Results of the Randomized Controlled Franconian Osteopenia and Sarcopenia Trial (FrOST). *J Bone Miner Res* **2020**, *35*:1634-1644.
40. Kemmler W, Kohl M, Jakob F, Engelke K, von Stengel S: Effects of High Intensity Dynamic Resistance Exercise and Whey Protein Supplements on Osteosarcopenia in Older Men with Low Bone and Muscle Mass. Final Results of the Randomized Controlled FrOST Study. *Nutrients* **2020**, *12*.
41. Krause M, Crognale D, Cogan KE, Contarelli S, Egan B, Newsholme P, De Vito G: The effects of a combined bodyweight-based and elastic bands resistance training, with or without protein supplementation, on muscle mass, signaling and heat shock response in healthy older people. *Exp Gerontol* **2019**, *115*:104-113.
42. Kukuljan S, Nowson CA, Bass SL, Sanders K, Nicholson GC, Seibel MJ, Salmon J, Daly RM: Effects of a multi-component exercise program and calcium-vitamin-D3-fortified milk on bone mineral density in older men: a randomised controlled trial. *Osteoporos Int* **2009**, *20*:1241-1251.
43. Kukuljan S, Nowson CA, Sanders K, Daly RM: Effects of resistance exercise and fortified milk on skeletal muscle mass, muscle size, and functional performance in middle-aged and older men: an 18-mo randomized controlled trial. *J Appl Physiol (1985)* **2009**, *107*:1864-1873.
44. Kwon J, Yoshida Y, Yoshida H, Kim H, Suzuki T, Lee Y: Effects of a combined physical training and nutrition intervention on physical performance and health-related quality of life in prefrail older women living in the community: a randomized controlled trial. *J Am Med Dir Assoc* **2015**, *16*:263.e261-268.
45. Lamb DA, Moore JH, Smith MA, Vann CG, Osburn SC, Ruple BA, Fox CD, Smith KS, Altonji OM, Power ZM, et al: The effects of resistance training with or without peanut protein supplementation on skeletal muscle and strength adaptations in older individuals. *J Int Soc Sports Nutr* **2020**, *17*:66.
46. Leenders M, Verdijk LB, Van der Hoeven L, Van Kranenburg J, Nilwik R, Wodzig WK, Senden JM, Keizer HA, Van Loon LJ: Protein supplementation during resistance-type exercise training in the elderly. *Med Sci Sports Exerc* **2013**, *45*:542-552.
47. Liao CD, Liao YH, Liou TH, Hsieh CY, Kuo YC, Chen HC: Effects of Protein-Rich Nutritional Composition Supplementation on Sarcopenia Indices and Physical Activity during Resistance Exercise Training in Older Women with Knee Osteoarthritis. *Nutrients* **2021**, *13*:2487.
48. Maesta N, Nahas EA, Nahas-Neto J, Orsatti FL, Fernandes CE, Traiman P, Burini RC: Effects of soy protein and resistance exercise on body composition and blood lipids in postmenopausal women. *Maturitas* **2007**, *56*:350-358.

49. Maltais ML, Ladouceur JP, Dionne IJ: The Effect of Resistance Training and Different Sources of Postexercise Protein Supplementation on Muscle Mass and Physical Capacity in Sarcopenic Elderly Men. *J Strength Cond Res* **2016**, *30***:**1680-1687.
50. McKenna CF, Salvador AF, Hughes RL, Scaroni SE, Alamilla RA, Askow AT, Paluska SA, Dilger AC, Holscher HD, De Lisio M: Higher protein intake during resistance training does not potentiate strength, but modulates gut microbiota, in middle-aged adults: a randomized control trial. *Am J Physiology-Endocrinology and Metabolism* **2021**, *320***:**E900-E913.
51. Mertz KH, Reitelseder S, Bechshoeft R, Bulow J, Højfeldt G, Jensen M, Schacht SR, Lind MV, Rasmussen MA, Mikkelsen UR, et al: The effect of daily protein supplementation, with or without resistance training for 1 year, on muscle size, strength, and function in healthy older adults: A randomized controlled trial. *Am J Clin Nutr* **2021**, *113***:**790-800.
52. Miller EG, Nowson CA, Dunstan DW, Kerr DA, Menzies D, Daly RM: Effects of whey protein plus vitamin D supplementation combined with progressive resistance training on glycaemic control, body composition, muscle function and cardiometabolic risk factors in middle-aged and older overweight/obese adults with type 2 diabetes: A 24-week randomized controlled trial. *Diabetes Obes Metab* **2021**, *23***:**938-949.
53. Mitchell CJ, D'Souza RF, Mitchell SM, Figueiredo VC, Miller BF, Hamilton KL, Peelor FF, 3rd, Coronet M, Pileggi CA, Durainayagam B, et al: Impact of dairy protein during limb immobilization and recovery on muscle size and protein synthesis; a randomized controlled trial. *J Appl Physiol (1985)* **2018**, *124***:**717-728.
54. Molnar A, Jonasne Sztruhar I, Csontos AA, Ferencz C, Varbiro S, Szekacs B: Special nutrition intervention is required for muscle protective efficacy of physical exercise in elderly people at highest risk of sarcopenia. *Physiol Int* **2016**, *103***:**368-376.
55. Mori H, Tokuda Y: Effect of whey protein supplementation after resistance exercise on the muscle mass and physical function of healthy older women: A randomized controlled trial. *Geriatr Gerontol Int* **2018**, *18***:**1398-1404.
56. Mori H, Tokuda Y: De-Training Effects Following Leucine-Enriched Whey Protein Supplementation and Resistance Training in Older Adults with Sarcopenia: A Randomized Controlled Trial with 24 Weeks of Follow-Up. *J Nutr Health Aging* **2022**, *26***:**994-1002.
57. Nabuco HCG, Tomeleri CM, Sugihara Junior P, Fernandes RR, Cavalcante EF, Antunes M, Ribeiro AS, Teixeira DC, Silva AM, Sardinha LB, Cyrino ES: Effects of Whey Protein Supplementation Pre- or Post-Resistance Training on Muscle Mass, Muscular Strength, and Functional Capacity in Pre-Conditioned Older Women: A Randomized Clinical Trial. *Nutrients* **2018**, *10*.
58. Nabuco HCG, Tomeleri CM, Sugihara Junior P, Fernandes RR, Cavalcante EF, Venturini D, Barbosa DS, Silva AM, Sardinha LB, Cyrino ES: Effects of pre- or post-exercise whey protein supplementation on body fat and metabolic and inflammatory profile in pre-conditioned older women: A randomized, double-blind, placebo-controlled trial. *Nutr Metab Cardiovasc Dis* **2019**, *29***:**290-300.
59. Nabuco HCG, Tomeleri CM, Fernandes RR, Sugihara Junior P, Cavalcante EF, Cunha PM, Antunes M, Nunes JP, Venturini D, Barbosa DS, et al: Effect of whey protein supplementation combined with resistance training on body composition, muscular strength, functional capacity, and plasma-metabolism biomarkers in older women with sarcopenic obesity: A randomized, double-blind, placebo-controlled trial. *Clin Nutr ESPEN* **2019**, *32***:**88-95.
60. Nabuco HCG, Tomeleri CM, Fernandes RR, Sugihara Junior P, Cavalcante EF, Venturini D, Barbosa DS, Silva AM, Sardinha LB, Cyrino ES: Effects of Protein Intake Beyond Habitual Intakes Associated With Resistance Training on Metabolic Syndrome-Related Parameters, Isokinetic Strength, and Body Composition in Older Women. *J Aging Phys Act* **2019**, *27***:**545–552.
61. Nilsson MI, Mikhail A, Lan L, Di Carlo A, Hamilton B, Barnard K, Hettinga BP, Hatcher E, Tarnopolsky MG, Nederveen JP, et al: A Five-Ingredient Nutritional Supplement and Home-Based Resistance Exercise Improve Lean Mass and Strength in Free-Living Elderly. *Nutrients* **2020**, *12*.
62. Oesen S, Halper B, Hofmann M, Jandrasits W, Franzke B, Strasser EM, Graf A, Tschan H, Bachl N, Quittan M, et al: Effects of elastic band resistance training and nutritional supplementation on physical performance of institutionalised elderly - A randomized controlled trial. *Exp Gerontol* **2015**, *72***:**99-108.
63. Orsatti FL, Maestá N, de Oliveira EP, Nahas Neto J, Burini RC, Nunes PRP, Souza AP, Martins FM, Nahas EP: Adding Soy Protein to Milk Enhances the Effect of Resistance Training on Muscle Strength in Postmenopausal Women. *J Diet Suppl* **2018**, *15***:**140-152.
64. Roschel H, Hayashi AP, Fernandes AL, Jambassi-Filho JC, Hevia-Larraín V, de Capitani M, Santana DA, Gonçalves LS, de Sá-Pinto AL, Lima FR, et al: Supplement-based nutritional strategies to tackle frailty: A multifactorial, double-blind, randomized placebo-controlled trial. *Clin Nutr* **2021**, *40***:**4849-4858.
65. Seino S, Sumi K, Narita M, Yokoyama Y, Ashida K, Kitamura A, Shinkai S: Effects of Low-Dose Dairy Protein Plus Micronutrient Supplementation during Resistance Exercise on Muscle Mass and Physical Performance in Older Adults: A Randomized, Controlled Trial. *J Nutr Health Aging* **2018**, *22***:**59-67.
66. Shahar S, Kamaruddin NS, Badrasawi M, Sakian NI, Abd Manaf Z, Yassin Z, Joseph L: Effectiveness of exercise and protein supplementation intervention on body composition, functional fitness, and oxidative stress among elderly Malays with sarcopenia. *Clin Interv Aging* **2013**, *8***:**1365-1375.

67. Shenoy S, Bedi R, Sandhu JS: Effect of soy isolate protein and resistance exercises on muscle performance and bone health of osteopenic/osteoporotic post-menopausal women. *J Women Aging* **2013**, *25***:**183-198.
68. Soares ALS, Machado-Lima A, Brech GC, Greve JMD, Dos Santos JR, Inojossa TR, Rogero MM, Salles JEN, Santarem-Sobrinho JM, Davis CL, Alonso AC: The Influence of Whey Protein on Muscle Strength, Glycemic Control and Functional Tasks in Older Adults with Type 2 Diabetes Mellitus in a Resistance Exercise Program: Randomized and Triple Blind Clinical Trial. *Int J Environ Res Public Health* **2023**, *20*.
69. Sugihara Junior P, Ribeiro AS, Nabuco HCG, Fernandes RR, Tomeleri CM, Cunha PM, Venturini D, Barbosa DS, Schoenfeld BJ, Cyrino ES: Effects of Whey Protein Supplementation Associated With Resistance Training on Muscular Strength, Hypertrophy, and Muscle Quality in Preconditioned Older Women. *Int J Sport Nutr Exerc Metab* **2018**, *28***:**528-535.
70. Tang W, Yan X, Fan P, Xu D, Li Y: Analysis of the Clincal Effect ofDietary Nutrition Intervention Combined with Resistance Training on the Cognitive Function of Elderly Male Patients with Sarcopenia. *Progr in Mod Biomed* **2020**, *20***:**2698-2701.
71. Thomson RL, Brinkworth GD, Noakes M, Buckley JD: Muscle strength gains during resistance exercise training are attenuated with soy compared with dairy or usual protein intake in older adults: A randomized controlled trial. *Clin Nutr* **2016**, *35***:**27-33.
72. Tieland M, Dirks ML, van der Zwaluw N, Verdijk LB, van de Rest O, de Groot LC, van Loon LJ: Protein supplementation increases muscle mass gain during prolonged resistance-type exercise training in frail elderly people: a randomized, double-blind, placebo-controlled trial. *J Am Med Dir Assoc* **2012**, *13***:**713-719.
73. Dirks ML, Tieland M, Verdijk LB, Losen M, Nilwik R, Mensink M, de Groot LCPGM, van Loon LJC: Protein Supplementation Augments Muscle Fiber Hypertrophy but Does Not Modulate Satellite Cell Content During Prolonged Resistance-Type Exercise Training in Frail Elderly. *J Am Med Dir Assoc* **2017**, *18***:**608-615.
74. Trevisan MC, Souza JM, Marucci Mde F: Influence of soy protein intake and weight training on the resting energy expenditure of postmenopausal women. *Rev Assoc Med Bras (1992)* **2010**, *56***:**572-578.
75. Unterberger S, Aschauer R, Zöhrer PA, Draxler A, Franzke B, Strasser EM, Wagner KH, Wessner B: Effects of an increased habitual dietary protein intake followed by resistance training on fitness, muscle quality and body composition of seniors: A randomised controlled trial. *Clin Nutr* **2022**, *41***:**1034-1045.
76. Verdijk LB, Jonkers RAM, Gleeson BG, Beelen M, Meijer K, Savelberg HHCM, Wodzig KWHW, Dendale P, Van Loon LJC: Protein supplementation before and after exercise does not further augment skeletal muscle hypertrophy after resistance training in elderly men. *American Journal of Clinical Nutrition* **2009**, *89***:**608-616.
77. Verreijen AM, Verlaan S, Engberink MF, Swinkels S, de Vogel-van den Bosch J, Weijs PJ: A high whey protein-, leucine-, and vitamin D-enriched supplement preserves muscle mass during intentional weight loss in obese older adults: A double-blind randomized controlled trial. *Am J Clin Nutr* **2015**, *101***:**279-286.
78. Vijayakumaran RK, Daly RM, Tan VPS: " We Want More"-Perspectives of Sarcopenic Older Women on the Feasibility of High-Intensity Progressive Resistance Exercises and a Whey-Protein Nutrition Intervention. *Front Nutr* **2023**, *10***:**1176523.
79. Vikberg S, Sörlén N, Brandén L, Johansson J, Nordström A, Hult A, Nordström P: Effects of Resistance Training on Functional Strength and Muscle Mass in 70-Year-Old Individuals With Pre-sarcopenia: A Randomized Controlled Trial. *J Am Med Dir Assoc* **2019**, *20***:**28-34.
80. Villanueva MG, He J, Schroeder ET: Periodized resistance training with and without supplementation improve body composition and performance in older men. *Eur J Appl Physiol* **2014**, *114***:**891-905.
81. Weisgarber KD, Candow DG, Farthing JP: Whey protein and high-volume resistance training in postmenopausal women. *J Nutr Health Aging* **2015**, *19***:**511-517.
82. Yamada M, Kimura Y, Ishiyama D, Nishio N, Otobe Y, Tanaka T, Ohji S, Koyama S, Sato A, Suzuki M, et al: Synergistic effect of bodyweight resistance exercise and protein supplementation on skeletal muscle in sarcopenic or dynapenic older adults. *Geriatr Gerontol Int* **2019**.
83. Zdzieblik D, Jendricke P, Oesser S, Gollhofer A, König D: The Influence of Specific Bioactive Collagen Peptides on Body Composition and Muscle Strength in Middle-Aged, Untrained Men: A Randomized Controlled Trial. *Int J Environ Res Public Health* **2021**, *18*.
84. Zhao T, Gao HL, Guo NZ, Chea ZM, Wu XY, He DL, Wei LQ, Shen XC, Lv WY: Effect of health education and whey protein supplementation combined with resistance exercise on the intervention of middle — aged and elderly people with sarcopenia. *J Clin Exp Med* **2022**, *12***:**90-94.
85. Zhu LY, Chan R, Kwok T, Cheng KC, Ha A, Woo J: Effects of exercise and nutrition supplementation in community-dwelling older Chinese people with sarcopenia: a randomized controlled trial. *Age Ageing* **2019**, *48***:**220-228.

Supplementary table S3. League table for pairwise and network meta-analysis of mean change in muscle mass from baseline

	Direct evidence of pairwise meta-analyses (row compared with column)											
	V1	V2	V3	V4	V5	V6	V7	V8	V9	V10	V11	V12
V1	Meat+RT					0.51 (-0.96, 1.97)					0.40 (-0.48, 1.28)	
V2	-0.02 (-0.84, 0.81)	MP+RT	0.36 (-0.61, 1.32)			-0.20 (-1.16, 0.76)		0.62 (-0.14, 1.39)		0.75 (-0.72, 2.22)	0.55 (0.17, 0.93)	1.31 (0.41, 2.22)
V3	-0.06 (-0.85, 0.73)	-0.05 (-0.42, 0.32)	WP+RT	0.60 (-0.68, 1.88)		0.12 (-1.20, 1.45)			0.95 (0.52, 1.37)		0.56 (0.33, 0.79)	1.36 (0.90, 1.83)
V4	0.36 (-0.87, 1.58)	0.37 (-0.63, 1.38)	0.42 (-0.54, 1.37)	Casein+RT							0.39 (-1.03, 1.80)	
V5	0.27 (-1.02, 1.57)	0.29 (-0.81, 1.39)	0.33 (-0.74, 1.41)	-0.08 (-1.51, 1.34)	Peanut+RT						0.25 (-0.81, 1.30)	
V6	0.17 (-0.64, 0.98)	0.19 (-0.28, 0.65)	0.23 (-0.19, 0.66)	-0.19 (-1.22, 0.84)	-0.10 (-1.22, 1.02)	SP+RT		0.56 (-0.87, 1.99)		1.21 (0.58, 1.85)	0.40 (-0.05, 0.85)	1.06 (0.49, 1.63)
V7	1.61 (-0.12, 3.34)	1.62 (0.04, 3.21)	1.67 (0.10, 3.24)	1.25 (-0.58, 3.08)	1.34 (-0.55, 3.22)	1.44 (-0.14, 3.01)	Meat			-0.45 (-1.92, 1.02)		
V8	0.77 (-0.21, 1.76)	0.79 (0.16, 1.42)	0.84 (0.18, 1.49)	0.42 (-0.73, 1.56)	0.50 (-0.73, 1.73)	0.60 (-0.09, 1.30)	-0.83 (-2.49, 0.82)	MP		0.19 (-1.28, 1.65)	-0.36 (-1.61, 0.90)	0.31 (-0.62, 1.24)
V9	0.87 (0.02, 1.72)	0.89 (0.39, 1.38)	0.93 (0.55, 1.32)	0.51 (-0.51, 1.53)	0.60 (-0.52, 1.72)	0.70 (0.17, 1.23)	-0.74 (-2.34, 0.87)	0.10 (-0.62, 0.82)	WP		-0.09 (-0.63, 0.45)	0.31 (-0.18, 0.81)
V10	1.16 (0.24, 2.07)	1.17 (0.57, 1.77)	1.22 (0.65, 1.79)	0.80 (-0.30, 1.90)	0.88 (-0.30, 2.07)	0.99 (0.42, 1.56)	-0.45 (-1.92, 1.02)	0.38 (-0.39, 1.15)	0.29 (-0.36, 0.93)	SP	-0.77 (-1.45,-0.08)	0.19 (-0.49, 0.87)
V11	0.52 (-0.24, 1.28)	0.53 (0.21, 0.86)	0.58 (0.36, 0.80)	0.16 (-0.80, 1.12)	0.25 (-0.81, 1.30)	0.35 (-0.04, 0.74)	-1.09 (-2.65, 0.47)	-0.26 (-0.89, 0.38)	-0.35 (-0.74, 0.04)	-0.64 (-1.18,-0.10)	RT	0.79 (0.42, 1.15)
V12	1.23 (0.41, 2.04)	1.24 (0.83, 1.65)	1.29 (0.96, 1.62)	0.87 (-0.13, 1.87)	0.95 (-0.14, 2.05)	1.06 (0.63, 1.49)	-0.38 (-1.95, 1.19)	0.45 (-0.19, 1.09)	0.36 (-0.07, 0.78)	0.07 (-0.48, 0.62)	0.71 (0.40, 1.02)	RC
	Relative effects of NMA (column compared with row)											

Pairwise (upper right portion) and network (lower left portion) meta-analysis results are presented for mean change (from baseline) in global function. Effect estimation is presented in standardized mean difference with 95% confidence interval. Significant results ($p < 0.05$) are marked in red.

MP, milk protein; RC, regular care; RT, resistance training; SP, soy protein; WP, whey protein.

Supplementary table S4. League table for pairwise and network meta-analysis of mean change in handgrip strength from baseline

	Direct evidence of pairwise meta-analyses (row compared with column)								
	V1	V2	V3	V4	V5	V6	V7	V8	V9
V1	WP+RT	0.43 (-1.50, 2.36)	.	1.93 (-0.04, 3.90)	.	0.86 (0.11, 1.61)	.	0.31 (-0.16, 0.79)	1.53 (0.88, 2.19)
V2	0.06 (-0.85, 0.98)	SP+RT	0.18 (-1.73, 2.09)	.	.	.	2.46 (0.44, 4.48)	0.45 (-0.52, 1.42)	1.51 (0.11, 2.90)
V3	0.19 (-0.55, 0.92)	0.12 (-0.88, 1.12)	MP+RT	.	.	.	.	0.39 (-0.26, 1.04)	0.22 (-1.68, 2.13)
V4	0.22 (-1.11, 1.55)	0.16 (-1.39, 1.71)	0.03 (-1.41, 1.48)	Casein+RT	.	.	.	-0.58 (-2.52, 1.37)	2.98 (0.99, 4.97)
V5	2.32 (0.59, 4.06)	2.26 (0.37, 4.15)	2.14 (0.33, 3.94)	2.10 (-0.02, 4.23)	Meat+RT	.	.	-1.83 (-3.79, 0.12)	-0.88 (-2.82, 1.05)
V6	0.99 (0.31, 1.67)	0.93 (-0.14, 2.00)	0.80 (-0.11, 1.72)	0.77 (-0.67, 2.21)	-1.33 (-3.15, 0.48)	WP	.	-0.38 (-1.27, 0.51)	0.12 (-0.77, 1.01)
V7	1.16 (-0.52, 2.85)	1.10 (-0.61, 2.82)	0.98 (-0.77, 2.72)	0.94 (-1.14, 3.03)	-1.16 (-3.51, 1.19)	0.17 (-1.59, 1.94)	SP	-0.03 (-2.00, 1.94)	0.90 (-1.08, 2.88)
V8	0.47 (0.03, 0.92)	0.41 (-0.45, 1.27)	0.29 (-0.32, 0.89)	0.25 (-1.07, 1.58)	-1.85 (-3.56, -0.14)	-0.52 (-1.22, 0.18)	-0.69 (-2.34, 0.96)	RT	0.56 (-0.13, 1.26)
V9	1.46 (0.92, 2.00)	1.40 (0.47, 2.33)	1.27 (0.50, 2.04)	1.24 (-0.08, 2.56)	-0.87 (-2.57, 0.84)	0.47 (-0.26, 1.20)	0.29 (-1.37, 1.96)	0.99 (0.44, 1.53)	RC
	Relative effects of NMA (column compared with row)								

Pairwise (upper right portion) and network (lower left portion) meta-analysis results are presented for mean change (from baseline) in global function. Effect estimation is presented in standardized mean difference with 95% confidence interval. Significant results ($p < 0.05$) are marked in red.

MP, milk protein; RC, regular care; RT, resistance training; SP, soy protein; WP, whey protein.

Supplementary table S5. League table for pairwise and network meta-analysis of mean change in leg strength from baseline

	Direct evidence of pairwise meta-analyses (row compared with column)										
	V1	V2	V3	V4	V5	V6	V7	V8	V9	V10	V11
V1	WP+RT	-0.12 (-1.02; 0.79)	0.74 (-0.50; 1.98)	.	0.33 (-0.85; 1.52)	1.30 (0.76; 1.84)	.	.	.	0.45 (0.20; 0.69)	1.30 (0.73; 1.87)
V2	0.08 (-0.32; 0.49)	MP+RT	0.32 (-0.87; 1.52)	.	.	.	.	0.70 (-0.13; 1.54)	.	0.34 (-0.08; 0.76)	0.69 (-0.47; 1.85)
V3	0.09 (-0.34; 0.51)	0.00 (-0.50; 0.50)	SP+RT	-0.42 (-1.81; 0.96)	.	.	1.92 (1.16; 2.68)	.	.	0.14 (-0.29; 0.57)	1.73 (1.14; 2.32)
V4	0.14 (-0.60; 0.88)	0.06 (-0.73; 0.85)	0.05 (-0.70; 0.81)	Meat+RT	.	.	.	.	.	0.13 (-0.68; 0.94)	.
V5	0.33 (-0.85; 1.52)	0.25 (-1.01; 1.50)	0.24 (-1.02; 1.50)	0.19 (-1.21; 1.59)	Casein+RT	.	.	.	.	.	.
V6	1.26 (0.78; 1.74)	1.17 (0.58; 1.77)	1.17 (0.58; 1.77)	1.12 (0.27; 1.97)	0.93 (-0.35; 2.21)	WP	.	.	.	-0.64 (-1.32; 0.03)	0.20 (-0.46; 0.85)
V7	1.38 (0.70; 2.05)	1.29 (0.57; 2.02)	1.29 (0.63; 1.95)	1.24 (0.29; 2.18)	1.04 (-0.32; 2.41)	0.12 (-0.66; 0.90)	SP	.	0.39 (-1.00; 1.77)	-0.58 (-1.47; 0.31)	0.41 (-0.32; 1.14)
V8	1.10 (0.39; 1.81)	1.02 (0.33; 1.70)	1.02 (0.26; 1.77)	0.96 (-0.02; 1.94)	0.77 (-0.61; 2.15)	-0.16 (-0.97; 0.66)	-0.27 (-1.18; 0.64)	MP	.	-0.47 (-1.63; 0.69)	-0.05 (-0.91; 0.82)
V9	1.76 (0.22; 3.30)	1.68 (0.11; 3.24)	1.67 (0.14; 3.21)	1.62 (-0.05; 3.30)	1.43 (-0.51; 3.37)	0.50 (-1.09; 2.09)	0.39 (-1.00; 1.77)	0.66 (-1.00; 2.32)	Meat	.	.
V10	0.43 (0.20; 0.66)	0.35 (-0.02; 0.71)	0.35 (-0.03; 0.72)	0.29 (-0.41; 1.00)	0.10 (-1.11; 1.31)	-0.83 (-1.31; -0.34)	-0.94 (-1.59; -0.30)	-0.67 (-1.36; 0.02)	-1.33 (-2.86; 0.20)	RT	0.94 (0.53; 1.36)
V11	1.41 (1.03; 1.79)	1.33 (0.86; 1.80)	1.32 (0.88; 1.77)	1.27 (0.49; 2.05)	1.08 (-0.17; 2.32)	0.15 (-0.37; 0.67)	0.03 (-0.61; 0.68)	0.31 (-0.38; 1.00)	-0.35 (-1.88; 1.18)	0.98 (0.63; 1.33)	RC
	Relative effects of NMA (column compared with row)										

Pairwise (upper right portion) and network (lower left portion) meta-analysis results are presented for mean change (from baseline) in global function. Effect estimation is presented in standardized mean difference with 95% confidence interval. Significant results ($p < 0.05$) are marked in red.

MP, milk protein; RC, regular care; RT, resistance training; SP, soy protein; WP, whey protein.

Supplementary table S6. League table for pairwise and network meta-analysis of mean change in walking speed from baseline

	Direct evidence of pairwise meta-analyses (row compared with column)								
	V1	V2	V3	V4	V5	V6	V7	V8	V9
V1	WP+RT	.	.	-0.29 (-1.51; 0.93)	0.31 (-0.09; 0.70)	.	.	0.20 (-0.09; 0.49)	0.84 (0.43; 1.25)
V2	0.14 (-0.50; 0.78)	SP+RT	.	-0.27 (-1.44; 0.89)	.	0.37 (-0.82; 1.56)	.	0.22 (-0.46; 0.90)	0.55 (-0.28; 1.37)
V3	0.17 (-0.62; 0.97)	0.03 (-0.92; 0.99)	Meat+RT	.	.	.	.	0.20 (-0.60; 1.01)	0.04 (-1.15; 1.22)
V4	0.20 (-0.24; 0.64)	0.06 (-0.61; 0.73)	0.03 (-0.81; 0.87)	MP+RT	.	.	0.72 (-0.11; 1.54)	-0.04 (-0.46; 0.37)	0.19 (-0.94; 1.32)
V5	0.20 (-0.17; 0.57)	0.06 (-0.63; 0.74)	0.02 (-0.81; 0.86)	-0.00 (-0.52; 0.52)	WP	.	.	0.18 (-0.31; 0.67)	0.76 (0.30; 1.22)
V6	0.37 (-0.65; 1.40)	0.23 (-0.82; 1.28)	0.20 (-1.05; 1.44)	0.17 (-0.89; 1.23)	0.17 (-0.88; 1.23)	SP	.	-0.13 (-1.32; 1.06)	0.47 (-0.72; 1.66)
V7	0.95 (0.15; 1.75)	0.81 (-0.14; 1.76)	0.78 (-0.29; 1.85)	0.75 (0.00; 1.50)	0.76 (-0.09; 1.60)	0.58 (-0.67; 1.83)	MP	-0.53 (-1.66; 0.60)	-0.28 (-1.41; 0.85)
V8	0.22 (-0.05; 0.49)	0.07 (-0.53; 0.67)	0.04 (-0.71; 0.80)	0.02 (-0.36; 0.39)	0.02 (-0.36; 0.40)	-0.16 (-1.16; 0.85)	-0.74 (-1.51; 0.03)	RT	0.59 (0.22; 0.95)
V9	0.73 (0.39; 1.07)	0.59 (-0.03; 1.21)	0.56 (-0.23; 1.35)	0.53 (0.07; 1.00)	0.53 (0.13; 0.94)	0.36 (-0.65; 1.37)	-0.22 (-1.02; 0.57)	0.52 (0.19; 0.84)	RC
	Relative effects of NMA (column compared with row)								

Pairwise (upper right portion) and network (lower left portion) meta-analysis results are presented for mean change (from baseline) in global function. Effect estimation is presented in standardized mean difference with 95% confidence interval. Significant results ($p < 0.05$) are marked in red.

MP, milk protein; RC, regular care; RT, resistance training; SP, soy protein; WP, whey protein.

Supplementary table S7. League table for pairwise and network meta-analysis of mean change in chair rise from baseline

	Direct evidence of pairwise meta-analyses (row compared with column)							
	V1	V2	V3	V4	V5	V6	V7	V8
V1	WP+RT	-0.00 (-0.71; 0.71)	0.25 (-0.57; 1.07)	.	.	0.85 (0.46; 1.25)	0.12 (-0.05; 0.29)	0.98 (0.64; 1.32)
V2	-0.04 (-0.46; 0.38)	SP+RT	.	.	.	.	0.17 (-0.29; 0.63)	0.92 (0.31; 1.54)
V3	0.22 (-0.04; 0.48)	0.26 (-0.20; 0.72)	MP+RT	.	0.31 (-0.37; 0.99)	.	-0.04 (-0.28; 0.20)	0.40 (-0.23; 1.03)
V4	0.49 (-0.11; 1.10)	0.53 (-0.18; 1.24)	0.27 (-0.35; 0.89)	Meat+RT	.	.	-0.35 (-0.93; 0.23)	.
V5	0.53 (-0.20; 1.26)	0.57 (-0.25; 1.39)	0.31 (-0.37; 0.99)	0.04 (-0.88; 0.95)	MP	.	.	.
V6	0.70 (0.36; 1.04)	0.74 (0.22; 1.25)	0.48 (0.08; 0.87)	0.20 (-0.47; 0.88)	0.17 (-0.62; 0.95)	WP	-0.55 (-0.95; -0.16)	0.02 (-0.36; 0.41)
V7	0.14 (-0.02; 0.31)	0.18 (-0.23; 0.59)	-0.08 (-0.30; 0.15)	-0.35 (-0.93; 0.23)	-0.38 (-1.10; 0.33)	-0.55 (-0.89; -0.21)	RT	0.82 (0.53; 1.11)
V8	0.84 (0.57; 1.10)	0.87 (0.44; 1.31)	0.62 (0.31; 0.92)	0.34 (-0.29; 0.97)	0.31 (-0.44; 1.05)	0.14 (-0.21; 0.49)	0.69 (0.44; 0.94)	RC
	Relative effects of NMA (column compared with row)							

Pairwise (upper right portion) and network (lower left portion) meta-analysis results are presented for mean change (from baseline) in global function. Effect estimation is presented in standardized mean difference with 95% confidence interval. Significant results (p <0.05) are marked in red.

MP, milk protein; RC, regular care; RT, resistance training; SP, soy protein; WP, whey protein.

Supplementary table S8. League table for pairwise and network meta-analysis of mean change in global physical mobility (SPPB) from baseline

	Direct evidence of pairwise meta-analyses (row compared with column)					
	V1	V2	V3	V4	V5	V6
V1	WP+RT	.	.	0.01 (-1.23; 1.25)	0.03 (-0.52; 0.58)	1.79 (1.09; 2.48)
V2	0.49 (-0.21; 1.18)	MP+RT	.	.	-0.03 (-0.62; 0.56)	0.33 (-0.80; 1.46)
V3	0.55 (-0.76; 1.86)	0.07 (-1.31; 1.44)	Casein+RT	.	.	0.90 (-0.26; 2.06)
V4	0.25 (-0.81; 1.31)	-0.24 (-1.40; 0.93)	-0.30 (-1.90; 1.29)	WP	0.32 (-0.91; 1.56)	0.63 (-0.64; 1.89)
V5	0.29 (-0.23; 0.80)	-0.20 (-0.74; 0.34)	-0.27 (-1.61; 1.07)	0.04 (-1.03; 1.11)	RT	0.30 (-0.95; 1.55)
V6	1.45 (0.84; 2.07)	0.97 (0.22; 1.71)	0.90 (-0.26; 2.06)	1.21 (0.11; 2.30)	1.17 (0.49; 1.84)	RC
	Relative effects of NMA (column compared with row)					

Pairwise (upper right portion) and network (lower left portion) meta-analysis results are presented for mean change (from baseline) in global function. Effect estimation is presented in standardized mean difference with 95% confidence interval. Significant results ($p < 0.05$) are marked in red.
MP, milk protein; RC, regular care; RT, resistance training; SPPB, Short Physical Performance Battery; WP, whey protein.

Supplementary table S9. League table for pairwise and network meta-analysis of mean change in timed up-and-go task from baseline

	Direct evidence of pairwise meta-analyses (row compared with column)					
	V1	V2	V3	V4	V5	V6
V1	MP+RT	.	.	.	0.21 (-0.49; 0.90)	0.33 (-0.16; 0.82)
V2	0.17 (-0.54; 0.89)	WP+RT	.	0.48 (-0.12; 1.08)	0.03 (-0.15; 0.22)	.
V3	0.18 (-0.56; 0.92)	0.01 (-0.31; 0.32)	Meat+RT	.	0.03 (-0.23; 0.28)	.
V4	0.62 (-0.25; 1.49)	0.45 (-0.08; 0.97)	0.44 (-0.14; 1.03)	SP+RT	-0.38 (-0.98; 0.21)	.
V5	0.21 (-0.49; 0.90)	0.03 (-0.15; 0.22)	0.03 (-0.23; 0.28)	-0.42 (-0.94; 0.11)	RT	.
V6	0.33 (-0.16; 0.82)	0.15 (-0.72; 1.02)	0.15 (-0.74; 1.03)	-0.30 (-1.30; 0.70)	0.12 (-0.73; 0.97)	RC
	Relative effects of NMA (column compared with row)					

Pairwise (upper right portion) and network (lower left portion) meta-analysis results are presented for mean change (from baseline) in global function.
Effect estimation is presented in standardized mean difference with 95% confidence interval.
MP, milk protein; RC, regular care; RT, resistance training; SP, soy protein; WP, whey protein.

Table S10. Associations of moderators with treatment efficiency for main outcomes.

Moderator	Muscle mass						Muscle strength											
							Handgrip strength						Leg muscle strength					
	N	B	SE	Median	95% CrI		N	B	SE	Median	95% CrI		N	B	SE	Median	95% CrI	
Participant factor																		
Age	65	–0.268	0.0021	–0.27	–0.836,	0.319	32	0.152	0.0032	0.159	–0.760,	1.027	58	–0.623	0.0021	–0.622	–1.226,	–0.035
BMI	63	0.331	0.0018	0.331	–0.162,	0.839	30	–0.868	0.0039	–0.870	–1.944,	0.251	45	0.506	0.0024	0.503	–0.157,	1.185
Sex distribution[b]	64	–0.484	0.0026	–0.468	–1.204,	0.227	**32**	**–1.719**	**0.0035**	**–1.720**	**–2.682,**	**–0.768**	46	–0.151	0.0030	–0.143	–1.018,	0.671
Area of population[c]	65	–0.472	0.0026	–0.468	–1.177,	0.242	**31**	**–1.036**	**0.0035**	**–1.036**	**–1.999,**	**–0.072**	**57**	**–0.860**	**0.0029**	**–0.855**	**–1.698,**	**–0.050**
Health status[d]	**65**	**0.743**	**0.0018**	**0.744**	**0.224,**	**1.247**	32	0.16	0.0042	0.156	–1.012,	1.33	58	–0.709	0.0026	–0.709	–1.416,	0.001
Study design factor																		
ROB[e]	65	0.209	0.0025	0.209	–0.492,	0.899	32	0.209	0.0053	0.227	–1.280,	1.666	58	0.689	0.0027	0.684	–0.062,	1.432
Follow-up duration	65	–0.07	0.0014	–0.072	–0.454,	0.315	32	0.386	0.0057	0.386	–1.233,	2.009	58	–0.430	0.0016	–0.432	–0.873,	0.041
Intervention factor																		
PS dose	65	0.239	0.0025	0.242	–0.475,	0.929	32	0.239	0.0025	0.242	–0.475,	0.929	**58**	**0.755**	**0.0025**	**0.752**	**0.060,**	**1.474**
% 1-RM	61	0.141	0.0019	0.141	–0.417,	0.696	29	0.048	0.0034	0.044	–0.873,	0.998	47	0.425	0.0023	0.419	–0.211,	1.079
Treatment duration	65	–0.025	0.0014	–0.023	–0.419,	0.338	32	0.801	0.0053	0.819	–0.757,	2.204	58	–0.378	0.0015	–0.379	–0.807,	0.052

To be continued.

Table S10. Continued.

Moderator	Physical mobility																							
	Walking speed						Chair rise						Timed up and go						SPPB					
	N	B	SE	Median	95% CrI		N	B	SE	Median	95% CrI		N	B	SE	Median	95% CrI		N	B	SE	Median	95% CrI	
Participant factor																								
Age	34	–0.093	0.002	–0.089	–0.655,	0.447	27	–0.555	0.0045	–0.554	–1.816	0.702	11	0.042	0.0215	0.003	–6.272,	6.998	13	–0.363	0.0085	–0.332	–2.92	2.021
BMI	33	0.113	0.0019	0.114	–0.403,	0.638	26	0.222	0.0037	0.219	–0.806,	1.247	11	0.064	0.0129	–0.044	–3.230,	4.488	13	**–1.846**	**0.0037**	**–1.843**	**–2.885,**	**–0.813**
Sex distribution[b]	34	0.254	0.0019	–0.260	–0.254,	0.772	26	–0.079	0.0133	–0.081	–3.873,	3.721	10	0.208	0.0366	0.003	–5.546,	7.263	13	**–1.614**	**0.003**	**–1.609**	**–2.471,**	**–0.785**
Area of population[c]	34	–0.154	0.0017	–0.154	–0.635,	0.335	27	0.172	0.0042	0.172	–1.004,	1.35	11	0.383	0.0242	0.027	–5.686,	10.551	13	0.147	0.0053	0.134	–1.302,	1.638
Health status[d]	34	–0.279	0.002	–0.278	–0.832,	0.268	27	–1.214	0.0061	–1.218	–2.863,	0.482	11	–0.123	0.0017	–0.022	–5.595,	4.467	13	0.149	0.038	0.201	–12.667,	11.774
Study design factor																								
ROB[e]	34	0.471	0.0024	0.469	–0.173,	1.136	27	0.173	0.0064	0.155	–0.587,	2.007	11	–0.242	0.0266	–0.021	–10.733,	5.884	13	0.54	0.0465	0.153	–12.252,	16.765
Follow-up duration	34	–0.346	0.0015	–0.346	–0.781,	0.088	27	–0.054	0.0045	–0.079	–1.242,	1.243	11	–0.653	0.0273	–0.045	–10.950,	5.832	13	**1.405**	**0.0023**	**1.404**	**0.772,**	**2.031**
Intervention factor																								
PS dose	34	–0.169	0.0022	–0.168	–0.768,	0.424	27	0.081	0.0038	0.082	–0.973,	1.161	11	0.09	0.0273	0.001	–8.695,	9.259	13	–1.2	0.0056	–1.196	–2.816,	0.372
% 1-RM	31	0.099	0.0015	0.01	–0.319,	0.521	25	0.249	0.0039	0.261	–0.372,	1.242	11	–0.021	0.0213	–0.01	–6.394,	6.862	13	–0.841	0.0041	–0.879	–1.940,	0.455
Treatment duration	34	–0.294	0.0015	–0.297	–0.713,	0.143	27	0.085	0.008	0.07	–2.114,	2.364	11	–0.421	0.0299	0.001	–14.443,	7.278	13	**1.411**	**0.0023**	**1.409**	**0.778,**	**2.051**

[a]Data represents the change in effects associated with the moderator indicated. B, beta coefficient; SE, standard error; 95% CrI, 95% credibility interval. Significant results ($p < 0.05$) are marked in bold.

[b]The proportion of female participants in sample.

[c]Code for regression model: America = 1; Asian = 2; Europe = 3; Oceania = 4.

[d]Code for regression model: Health = 1; Subhealthy = 2.

[e]Code for regression model: Low risk of bias = 0; High risk of bias = 1.

% 1-RM, percentage of one repetition maximum; 95% CrI, credible interval; BMI, body mass index; PS, protein supplementation; ROB, risk of bias; SPPB, Short Physical Performance Battery.

Table S11. Summary of compliance & adverse events.

Study (year) (reference number)[a]	Study arm	Group sample (n)	Withdraw, attrition rate, or drop out (number of patients)				Side effects and complications (number of patients)				Serious adverse event[b] (number of patients)		
			Related to PS	Related to RT	Unrelated to treatment	Total sum	Related to PS	Related to RT	Unrelated to treatment	Total sum	Related to treatment	Unrelated to treatment	Total sum
Aas 2019 [1]	Gr 1: MP+RT	11	0	0	0	0	0	0	0	0	0	0	0
	Gr 2: RC	11			0	0			0	0	0	0	0
Amasene 2019 [2]; 2021 [3]	Gr 1: WP+RT	21	2	3	1	6	1	0	0	1	0	1	1
	Gr 2: PLA+RT	20	0	3	4	7	0	0	0	0	0	2	2
Arciero 2014 [4]	Gr 1: WP+RT	27	1	4	0	5	NR	NR	NR		NR	NR	
	Gr 2: WP	24	6		0	6	NR		NR		NR	NR	
Arnarson 2013 [5]	Gr 1: WP+RT	83	0	0	8	8	NR	NR	NR		NR	NR	
	Gr 2: PLA+RT	78	0	0	12	12	NR	NR	NR		NR	NR	
Bagheri 2022 [6]	Gr 1: WP+RT	14	0	0	0	0	0	0	0	0	0	0	0
	Gr 2: PLA+RT	14	0	0	0	0	0	0	0	0	0	0	0
Bijeh 2022 [7]	Gr 1: SP+RT	15	0	0	0	0	0	0	0	0	0	0	0
	Gr 2: RT	15		0	0	0		0	0	0	0	0	0
	Gr 3: SP	15	0		0	0	0		0	0	0	0	0
	Gr 4: RC	15			0	0			0	0	0	0	0
Candow 2006 [8]	Gr 1: WP+RT	25	0	0	6	6	NR	NR	NR		NR	NR	
	Gr 2: PLA+RT	13	0	0	3	3	NR	NR	NR		NR	NR	
Candow 2008 [9]	Gr 1: WP+Cre+RT	12	0	0	2	2	1	0	0	1	NR	NR	
	Gr 2: Cre+RT	14	0	0	1	1	1	1	0	2	NR	NR	
	Gr 3: PLA+RT	14	0	0	2	2	2	2	0	4	0	0	0
Chale 2013 [10]	Gr 1: WP+RT	42	0	0	3	3	4	1	9	14	0	6	6
	Gr 2: PLA+RT	38	0	0	2	2	2	3	15	20	0	3	3
Chang 2019 [11]	Gr 1: Casein+RT	28	0	0	0	0	NR	NR	NR		NR	NR	
	Gr 2: RC	28			0	0			NR		NR	NR	
Chen 2017 [12]	Gr 1: WP+RT	20	0	0	0	0	NR	NR	NR		NR	NR	
	Gr 2: RC	20			0	0			NR		NR	NR	
Colonetti 2023 [13]	Gr 1: WP+RT	10	0	2	0	2	0	0	0	0	0	0	0
	Gr 2: PLA+RT	10	0	0	0	0	0	0	0	0	0	0	0
	Gr 3: RC	10			2	2			0	0	0	0	0
Daly 2014 [14]	Gr 1: Meat+RT	53	0	0	5	5	0	2	0	2	0	0	0
	Gr 2: CHO+RT	47	0	0	4	4	0	2	0	2	0	0	0
de Azevedo Bach 2022 [15]	Gr 1: WP+RT	18	0	3	0	3	0	0	0	0	0	0	0
	Gr 2: PLA+RT	18	1	1	0	2	0	0	0	0	0	0	0
de Carvalho Bastone 2020 [16]	Gr 1: WP+RT	20	0	0	4	4	NR	NR	NR		NR	NR	
	Gr 2: RT	20		0	3	3		NR	NR		NR	NR	
	Gr 3: WP	20	0		2	2	NR		NR		NR	NR	
	Gr 4: RC	20			2	2			NR		NR	NR	
Deer 2019 [17]	Gr 1: WP+RT	20	0	0	4	4	NR	NR	NR		NR	NR	
	Gr 2: PLA+RT	21	0	0	4	4	NR	NR	NR		NR	NR	
	Gr 3: WP	20	0		5	5	NR		NR		NR	NR	
	Gr 4: PLA	20	0		7	7	NR		NR		NR	NR	

Deibert 2011 [18]	Gr 1: SP+RT	14	0	0	1	1	NR	NR	NR		NR	NR	
	Gr 2: RT	14		0	1	1		NR	NR		NR	NR	
	Gr 3: RC	12			3	3			NR		NR	NR	
Duff 2014 [19]	Gr 1: MP+RT	19	0	0	0	0	2	0	0	2	0	0	0
	Gr 2: WP+RT	21	0	0	1	1	3	0	0	3	0	0	0
Dulac 2021 [20]	Gr 1: WP+RT	25	0	0	4	4	0	0	0	0	0	0	0
	Gr 2: Casein+RT	25	0	0	5	5	0	0	0	0	0	0	0
	Gr 3: PLA+RT	25	0	0	6	6	0	0	0	0	0	0	0
Eliot 2008 [21]; Bemben 2010 [22]	Gr 1: WP+Cre+RT	11	0	0	0	0	0	0	0	0	0	0	0
	Gr 2: WP+RT	11	0	0	0	0	0	0	0	0	0	0	0
	Gr 3: Cre+RT	10	0	0	0	0	0	0	0	0	0	0	0
	Gr 4: PLA+RT	10	0	0	0	0	0	0	0	0	0	0	0
Fiatarone 1994 [23]	Gr 1: SP+RT	25	0	0	1	1	1	1	0	2	0	0	0
	Gr 2: PLA+RT	25	0	1	1	2	0	2	1	3	0	0	0
	Gr 3: SP	24	0		2	2	1		0	1	0	0	0
	Gr 4: PLA	26	0		1	1	0		0	0	0	0	0
Formica 2020 [24]	Gr 1: Meat+RT	77	0	0	4	4	0	6	0	6	0	0	0
	Gr 2: RT	77		0	5	5		7	0	7	0	0	0
Francis 2017 [25]	Gr 1: MP+RT	29	7	0	13	20	NR	NR	NR		NR	NR	
	Gr 2: MP	28	9		13	22	NR		NR		NR	NR	
Gade 2019 [26]	Gr 1: MP+RT	83	0	2	3	5	0	0	0	0	0	0	0
	Gr 2: PLA+RT	82	0	1	4	5	0	0	0	0	0	0	0
Granic 2020 [27]	Gr 1: Whole-MP+RT	10	0	0	1	1	0	0	0	0	0	0	0
	Gr 2: Skimmed-MP+	10	0	0	0	0	0	0	0	0	0	0	0
	Gr 3: CHO+RT	10	0	0	0	0	0	0	0	0	0	0	0
Griffen 2022 [28]	Gr 1: WP+RT	9	0	0	0	0	0	0	0	0	0	0	0
	Gr 2: PLA+RT	10	0	0	1	1	0	1	0	1	0	0	0
	Gr 3: WP	10	0		1	1	0		0	0	0	0	0
	Gr 4: PLA	10	0		1	1	0		0	0	0	0	0
Gronstedt 2020 [29]	Gr 1: MP+RT	60	0	0	8	8	3	0	0	3	0	11	11
	Gr 2: RT	60		0	10	10		1	0	1	0	13	13
Hamarsland 2019 [30]	Gr 1: MP+RT	19	0	0	4	4	NR	NR	NR		NR	NR	
	Gr 2: WP+RT	19	0	0	4	4	NR	NR	NR		NR	NR	
Haß 2022 [31]	Gr 1: WP+Omega-3+	28	2	0	5	7	NR	NR	NR		NR	NR	
	Gr 2: WP+RT	27	2	0	5	7	NR	NR	NR		NR	NR	
	Gr 3: RT	22		0	2	2		NR	NR		NR	NR	
Haub 2002 [32]; 2005 [33]	Gr 1: Meat+RT	13	0	1	2	3	0	1	0	1	NR	NR	
	Gr 2: SP+RT	13	0	0	2	2	0	0	1	1	NR	NR	
He 2022 [34]	Gr 1: WP+RT	75	0	0	0	0	NR	NR	NR		NR	NR	
	Gr 2: RC	75			0	0			NR		NR	NR	
Holm 2008 [35]	Gr 1: WP+RT	13	0	0	5	5	NR	NR	NR		NR	NR	
	Gr 2: PLA+RT	16	0	0	4	4	NR	NR	NR		NR	NR	
Holwerda 2018 [36]	Gr 1: WP+RT	22	0	1	0	1	0	1	0	1	0	0	0
	Gr 2: PLA+RT	22	0	2	0	2	0	1	0	1	0	0	0
Kang 2020 [37]	Gr 1: MP+RT	60	0	0	6	6	1	0	2	3	0	0	0

	Gr 2: PLA+RT	60	0	2	3	5	0	0	0	0	0	0	0
Karelis 2015[38]	Gr 1: WP+RT	49	1	3	4	8	1	2	0	3	0	0	0
	Gr 2: Casein+RT	50	1	2	4	7	1	1	0	2	0	0	0
Kemmler 2020a [39]; 2020b [40]	Gr 1: WP+RT	21	0	0	2	2	NR	NR	NR		NR	NR	
	Gr 2: WP	22	0		2	2	NR		NR		NR	NR	
Krause 2019 [41]	Gr 1: WP+RT	11	0	0	0	0	NR	NR	NR		NR	NR	
	Gr 2: PLA+RT	10	0	0	0	0	NR	NR	NR		NR	NR	
	Gr 3: WP	7	0		0	0	NR		NR		NR	NR	
	Gr 4: PLA	10	0		0	0	NR		NR		NR	NR	
Kukuljan 2009a [42]; 2009b [43]	Gr 1: MP+RT	45	0	0	1	1	0	5	0	5	0	0	0
	Gr 2: RT	46		0	1	1		5	0	5	0	0	0
	Gr 3: MP	45	0		1	1	0		0	0	0	0	0
	Gr 4: RC	44			2	2			0	0	0	0	0
Kwon 2015 [44]	Gr 1: Meat+RT	30	0	0	4	4	NR	NR	NR		NR	NR	
	Gr 2: RT	28		0	3	3		NR	NR		NR	NR	
	Gr 3: RC	31			3	3			NR		NR	NR	
Lamb 2020 [45]	Gr 1: Peanut+RT	22	0	1	1	2	NR	NR	NR		NR	NR	
	Gr 2: RT	19		0	0	0		NR	NR		NR	NR	
Leenders 2013 [46]	Gr 1: MP+RT	30	0	0	3	3	NR	NR	NR		NR	NR	
	Gr 2: PLA+RT	30	0	0	4	4	NR	NR	NR		NR	NR	
Liao 2021 [47]	Gr 1: WP+RT	36	0	0	1	1	0	0	0	0	0	0	0
	Gr 2: RT	36		0	2	2		0	0	0	0	0	0
Maesta 2007 [48]	Gr 1: SP+RT	15	1	0	0	1	1	0	0	1	0	0	0
	Gr 2: PLA+RT	15	2	0	2	4	2	0	0	2	0	0	0
	Gr 3: SP	15	3		2	5	3		0	3	0	0	0
	Gr 4: PLA	15	3		1	4	3		0	3	0	0	0
McKenna 2021 [49]	Gr 1: Meat+RT	28	0	0	7	7	NR	NR	NR		NR	NR	
	Gr 2: RT	22		1	3	4		NR	NR		NR	NR	
Mertz 2021 [50]	Gr 1: WP+RT	72	0	0	10	10	NR	NR	NR		NR	NR	
	Gr 2: WP	50	0		6	6	NR		NR		NR	NR	
	Gr 3: PLA	36	0		2	2	NR		NR		NR	NR	
Miller 2021 [51]	Gr 1: WP+RT	98	0	1	10	11	35	11	0	46	0	0	0
	Gr 2: RT	100		3	17	20		12	0	12	0	0	0
Mitchell 2018 [52]	Gr 1: MP+RT	15	0	0	0	0	NR	NR	NR		NR	NR	
	Gr 2: PLA+RT	15	0	0	0	0	NR	NR	NR		NR	NR	
Molnar 2016 [53]	Gr 1: WP+RT	17	0	0	0	0	NR	NR	NR		NR	NR	
	Gr 2: RT	17		0	0	0		NR	NR		NR	NR	
Mori 2018 [54]	Gr 1: WP+RT	27	1	0	3	4	NR	NR	NR		NR	NR	
	Gr 2: RT	27		0	4	4		NR	NR		NR	NR	
	Gr 3: WP	27	1		2	3	NR		NR		NR	NR	
Mori 2022 [55]	Gr 1: WP+RT	27	0	0	2	2	NR	NR	NR		NR	NR	
	Gr 2: RT	27		0	2	2		NR	NR		NR	NR	
	Gr 3: WP	27	0		2	2	NR		NR		NR	NR	
Nabuco 2018 [56];	Gr 1: WP+RT	47	0	0	4	4	NR	NR	NR		NR	NR	
	Gr 2: PLA+RT	23	0	0	0	0	NR	NR	NR		NR	NR	

2019a [57]												
Nabuco 2019b [58]	Gr 1: WP+RT	13	0	0	0	0	NR	NR	NR		NR	NR	
	Gr 2: PLA+RT	13	0	0	0	0	NR	NR	NR		NR	NR	
Nabuco 2019c [59]	Gr 1: WP+RT	15	0	0	0	0	NR	NR	NR		NR	NR	
	Gr 2: PLA+RT	15	0	0	0	0	NR	NR	NR		NR	NR	
Nilsson 2020 [60]	Gr 1: MP+RT	22	0	0	6	6	NR	NR	NR		NR	NR	
	Gr 2: PLA+RT	23	0	0	7	7	NR	NR	NR		NR	NR	
Oesen 2015 [61]	Gr 1: WP+RT	36	0	0	11	11	0	0	0	0	0	0	0
	Gr 2: RT	41		0	10	10		0	0	0	0	0	0
	Gr 3: RC	40			14	14			0	0	0	0	0
Orsatti 2018 [62]	Gr 1: SP+RT	21	0	2	3	5	0	0	0	0	0	0	0
	Gr 2: PLA+RT	20	0	2	2	4	0	0	0	0	0	0	0
Roschel 2021 [63]	Gr 1: WP+RT	22	0	0	3	3	NR	NR	NR		NR	NR	
	Gr 2: SP+RT	22	0	0	2	2	NR	NR	NR		NR	NR	
	Gr 3: PLA+RT	22	0	0	1	1	NR	NR	NR		NR	NR	
Seino 2018 [64]	Gr 1: MP+RT	41	3	0	0	3	2	0	0	2	0	0	0
	Gr 2: RT	41		0	1	1		0	0	0	0	0	0
Shahar 2013 [65]	Gr 1: SP+RT	17	0	0	2	2	NR	NR	NR		NR	NR	
	Gr 2: RT	21		0	2	2		NR	NR		NR	NR	
	Gr 3: SP	18	0		3	3	NR		NR		NR	NR	
	Gr 4: RC	19			3	3			NR		NR	NR	
Shenoy 2013 [66]	Gr 1: SP+RT	20	0	0	0	0	NR	NR	NR		NR	NR	
	Gr 2: SP	20	0		0	0	NR		NR		NR	NR	
	Gr 3: RC	20			0	0			NR		NR	NR	
Soares 2023 [67]	Gr 1: WP+RT	14	0	0	1	1	NR	NR	NR		NR	NR	
	Gr 2: PLA+RT	14	0	0	1	1	NR	NR	NR		NR	NR	
Sugihara Junior 2018 [68]	Gr 1: WP+RT	15	0	0	0	0	NR	NR	NR		NR	NR	
	Gr 2: PLA+RT	16	0	0	0	0	NR	NR	NR		NR	NR	
Tang 2020 [69]	Gr 1: WP+RT	100	0	0	0	0	NR	NR	NR		NR	NR	
	Gr 2: RC	100			0	0			NR		NR	NR	
Thomson 2016 [70]	Gr 1: MP+RT	61	5	10	12	27	3	13	0	16	0	0	0
	Gr 2: SP+RT	64	6	20	12	38	1	13	0	14	0	0	0
	Gr 3: CHO+RT	62	15	13	11	39	4	12	0	16	0	0	0
Tieland 2012 [71]; Dirks 2017 [72]	Gr 1: MP+RT	31	0	0	5	5	NR	NR	NR		NR	NR	
	Gr 2: PLA+RT	31	0	0	6	6	NR	NR	NR		NR	NR	
Trevisan 2010 [73]	Gr 1: SP+RT	15	0	0	0	0	NR	NR	NR		NR	NR	
	Gr 2: PLA+RT	15	0	0	0	0	NR	NR	NR		NR	NR	
	Gr 3: SP	15	0		0	0	NR		NR		NR	NR	
	Gr 4: PLA	15	0		0	0	NR		NR		NR	NR	
Unterberger 2022 [74]	Gr 1: SP+RT	48	0	0	9	9	0	0	0	0	0	0	0
	Gr 2: PLA+RT	41	0	0	5	5	0	0	0	0	0	0	0
	Gr 3: RC	47			6	6			0	0	0	0	0
Verdijk 2009 [75]	Gr 1: Casein+RT	14	0	0	1	1	NR	NR	NR		NR	NR	
	Gr 2: PLA+RT	14	0	0	1	1	NR	NR	NR		NR	NR	
Verreijen 2015 [76]	Gr 1: WP+RT	40	0	3	5	8	0	3	0	3	0	0	0
	Gr 2: PLA+RT	40	0	3	4	7	0	3	0	3	0	0	0

Vijayakumaran 2023 [77]	Gr 1: WP+RT	8	0	0	0	0	0	0	0	0	0	0	0
	Gr 2: RT	8		0	0	0		0	0	0	0	0	0
Vikberg 2019 [78]	Gr 1: MP+RT	36	0	0	5	5	0	4	1	5	0	0	0
	Gr 2: RC	34			1	1			0	0	0	0	0
Villanueva 2014 [79]	Gr 1: WP+RT	8	0	0	1	1	0	0	0	0	0	0	0
	Gr 2: RT	8		0	1	1		0	0	0	0	0	0
	Gr 3: RC	9			1	1			0	0	0	0	0
Weisgarber 2015 [80]	Gr 1: WP+RT	17	0	0	2	2	0	0	0	0	0	0	0
	Gr 2: PLA+RT	17	0	0	3	3	0	0	0	0	0	0	0
Yamada 2019 [81]	Gr 1: WP+RT	28	2	2	2	6	0	0	0	0	0	0	0
	Gr 2: RT	28		2	2	4		0	0	0	0	0	0
	Gr 3: WP	28	4		2	6	0		0	0	0	0	0
	Gr 4: RC	28			2	2			0	0	0	0	0
Zdzieblik 2021 [82]	Gr 1: WP+RT	36	0	4	0	4	0	0	0	0	0	0	0
	Gr 2: PLA+RT	31	0	9	0	9	0	0	0	0	0	0	0
Zhao 2022 [83]	Gr 1: WP+RT	20	0	0	0	0	NR	NR	NR		NR	NR	
	Gr 2: WP	20	0			0	NR		NR		NR	NR	
	Gr 3: RC	20			0	0			NR		NR	NR	
Zhu 2019 [84]	Gr 1: SP+RT	36	0	0	8	8				4[c]			12[c]
	Gr 2: RT	40		0	16	16							
	Gr 3: RC	37			13	13							
Maltais 2016 [85]	Gr 1: MP+RT	10	0	0	2	2	NR	NR	NR		NR	NR	
	Gr 2: SP+RT	10	0	0	2	2	NR	NR	NR		NR	NR	
	Gr 3: PLA+RT	10	0	0	0	0	NR	NR	NR		NR	NR	

[a]The number denotes the citation of the indicated reference at the end of table.

[b]The reported events include cardiovascular onsets, fall injury, death, and other illness requiring inpatient hospitalization.

[c]Total number of participants in study.

CHO, carbohydrates; Cre, creatine; MP, milk protein; NR, not report; PLA, placebo; PS, protein supplementation; RT, resistance training; SP, soy protein; WP, whey protein RC, regular care.

References of supplementary Table S11

1. Aas SN, Seynnes O, Benestad HB, Raastad T: Strength training and protein supplementation improve muscle mass, strength, and function in mobility-limited older adults: a randomized controlled trial. *Aging Clin Exp Res* **2019**.
2. Amasene M, Besga A, Echeverria I, Urquiza M, Ruiz JR, Rodriguez-Larrad A, Aldamiz M, Anaut P, Irazusta J, Labayen I: Effects of Leucine-Enriched Whey Protein Supplementation on Physical Function in Post-Hospitalized Older Adults Participating in 12-Weeks of Resistance Training Program: A Randomized Controlled Trial. *Nutrients* **2019**, *11*.
3. Amasene M, Cadenas-Sanchez C, Echeverria I, Sanz B, Alonso C, Tobalina I, Irazusta J, Labayen I, Besga A: Effects of Resistance Training Intervention along with Leucine-Enriched Whey Protein Supplementation on Sarcopenia and Frailty in Post-Hospitalized Older Adults: Preliminary Findings of a Randomized Controlled Trial. *J Clin Med* **2021**, *11*.
4. Arciero PJ, Baur D, Connelly S, Ormsbee MJ: Timed-daily ingestion of whey protein and exercise training reduces visceral adipose tissue mass and improves insulin resistance: the PRISE

study. *Journal of applied physiology (Bethesda, Md : 1985)* **2014**, *117*:1-10.

5. Arnarson A, Gudny Geirsdottir O, Ramel A, Briem K, Jonsson PV, Thorsdottir I: Effects of whey proteins and carbohydrates on the efficacy of resistance training in elderly people: Double blind, randomised controlled trial. *Eur J Clin Nutr* **2013**, *67*:821-826.
6. Bagheri R, Hooshmand Moghadam B, Candow DG, Elliott BT, Wong A, Ashtary-Larky D, Forbes SC, Rashidlamir A: Effects of Icelandic yogurt consumption and resistance training in healthy untrained older males. *Br J Nutr* **2022**, *127*:1334-1342.
7. Bijeh N, Mohammadnia-Ahmadi M, Hooshamnd-Moghadam B, Eskandari M, Golestani F: Effects of Soy Milk in Conjunction With Resistance Training on Physical Performance and Skeletal Muscle Regulatory Markers in Older Men. *Biol Res Nurs* **2022**, *24*:294-307.
8. Candow DG, Chilibeck PD, Facci M, Abeysekara S, Zello GA: Protein supplementation before and after resistance training in older men. *Eur J Appl Physiol* **2006**, *97*:548-556.
9. Candow DG, Little JP, Chilibeck PD, Abeysekara S, Zello GA, Kazachkov M, Cornish SM, Yu PH: Low-dose creatine combined with protein during resistance training in older men. *Med Sci Sports Exerc* **2008**, *40*:1645-1652.
10. Chale A, Cloutier GJ, Hau C, Phillips EM, Dallal GE, Fielding RA: Efficacy of whey protein supplementation on resistance exercise-induced changes in lean mass, muscle strength, and physical function in mobility-limited older adults. *The journals of gerontology Series A, Biological sciences and medical sciences* **2013**, *68*:682-690.
11. Chang YM: Effects of resistance training combined with nutritional intervention on alleviating sarcopenia in the elderly. *Electron J Practical Clin Nurs Sci* **2019**, *4*:48-50.
12. Chen S, Sheng YL, Qi T, Sheng XM, Jin XW: Effects of fortified nutrition combining with resistance exercise on physical function and activity of daily living for elderly patients with sarcopenia. *J Nurs Sci* **2017**, *32*:8-10.
13. Colonetti T, Grande AJ, da Rocha FR, Ronconi Dondossola E, Tuon L, Gomes Batista Teles H, Minotto Bom B, Colonetti L, da Rosa MI: Whey protein and vitamin D supplementation in institutionalized older adults: A randomized trial. *Nutr Health* **2023**, *29*:129-138.
14. Daly RM, O'Connell SL, Mundell NL, Grimes CA, Dunstan DW, Nowson CA: Protein-enriched diet, with the use of lean red meat, combined with progressive resistance training enhances lean tissue mass and muscle strength and reduces circulating IL-6 concentrations in elderly women: a cluster randomized controlled trial. *Am J Clin Nutr* **2014**, *99*:899-910.
15. de Azevedo Bach S, Radaelli R, Beck Schemes M, Neske R, Garbelotto C, Roschel H, Silveira Pinto R, Dornelles Schneider C: Can supplemental protein to low-protein containing meals superimpose on resistance-training muscle adaptations in older adults? A randomized clinical trial. *Exp Gerontol* **2022**, *162*:111760.
16. de Carvalho Bastone A, Nobre LN, de Souza Moreira B, Rosa IF, Ferreira GB, Santos DDL, Monteiro N, Alves MD, Gandra RA, de Lira EM: Independent and combined effect of home-based progressive resistance training and nutritional supplementation on muscle strength, muscle mass and physical function in dynapenic older adults with low protein intake: A randomized controlled trial. *Arch Gerontol Geriatr* **2020**, *89*:104098.
17. Deer RR, Dickinson JM, Baillargeon J, Fisher SR, Raji M, Volpi E: A Phase I Randomized Clinical Trial of Evidence-Based, Pragmatic Interventions to Improve Functional Recovery After

Hospitalization in Geriatric Patients. *J Gerontol A Biol Sci Med Sci* **2019**, *74*:1628-1636.

18. Deibert P, Solleder F, König D, Vitolins MZ, Dickhuth HH, Gollhofer A, Berg A: Soy protein based supplementation supports metabolic effects of resistance training in previously untrained middle aged males. *Aging Male* **2011**, *14*:273-279.
19. Duff WR, Chilibeck PD, Rooke JJ, Kaviani M, Krentz JR, Haines DM: The effect of bovine colostrum supplementation in older adults during resistance training. *Int J Sport Nutr Exerc Metab* **2014**, *24*:276-285.
20. Dulac MC, Pion CH, Lemieux FC, Pinheiro Carvalho L, El Hajj Boutros G, Bélanger M, Gaudreau P, Chevalier S, Morais JA, Noirez P, et al: Effects of slow- v. fast-digested protein supplementation combined with mixed power training on muscle function and functional capacities in older men. *Br J Nutr* **2021**, *125*:1017-1033.
21. Eliot KA, Knehans AW, Bemben DA, Witten MS, Carter J, Bemben MG: The effects of creatine and whey protein supplementation on body composition in men aged 48 to 72 years during resistance training. *Journal of Nutrition, Health and Aging* **2008**, *12*:208-212.
22. Bemben MG, Witten MS, Carter JM, Eliot KA, Knehans AW, Bemben DA: The effects of supplementation with creatine and protein on muscle strength following a traditional resistance training program in middle-aged and older men. *J Nutr Health Aging* **2010**, *14*:155-159.
23. Fiatarone MA, O'Neill EF, Ryan ND, Clements KM, Solares GR, Nelson ME, Roberts SB, Kehayias JJ, Lipsitz LA, Evans WJ: Exercise Training and Nutritional Supplementation for Physical Frailty in Very Elderly People. *New England J Med* **1994**, *330*:1769-1775.
24. Formica MB, Gianoudis J, Nowson CA, O'Connell SL, Milte C, Ellis KA, Daly RM: Effect of lean red meat combined with a multicomponent exercise program on muscle and cognitive function in older adults: a 6-month randomized controlled trial. *Am J Clin Nutr* **2020**, *112*:113-128.
25. Francis P, Mc Cormack W, Toomey C, Norton C, Saunders J, Kerin E, Lyons M, Jakeman P: Twelve weeks' progressive resistance training combined with protein supplementation beyond habitual intakes increases upper leg lean tissue mass, muscle strength and extended gait speed in healthy older women. *Biogerontology* **2017**, *18*:881-891.
26. Gade J, Beck AM, Andersen HE, Christensen B, Rønholt F, Klausen TW, Vinther A, Astrup A: Protein supplementation combined with low-intensity resistance training in geriatric medical patients during and after hospitalisation: a randomised, double-blind, multicentre trial. *Br J Nutr* **2019**, *122*:1006-1020.
27. Granic A, Hurst C, Dismore L, Stevenson E, Sayer AA, Aspray T: Feasibility and acceptability of a milk and resistance exercise intervention to improve muscle function in community-dwelling older adults (MIlkMAN): Pilot study. *PLoS One* **2020**, *15*:e0235952.
28. Griffen C, Duncan M, Hattersley J, Weickert MO, Dallaway A, Renshaw D: Effects of resistance exercise and whey protein supplementation on skeletal muscle strength, mass, physical function, and hormonal and inflammatory biomarkers in healthy active older men: a randomised, double-blind, placebo-controlled trial. *Exp Gerontol* **2022**, *158*:111651.
29. Gronstedt H, Vikstrom S, Cederholm T, Franzen E, Luiking YC, Seiger A, Wimo A, Faxen-Irving G, Bostrom AM: Effect of Sit-to-Stand Exercises Combined With Protein-Rich Oral Supplementation in Older Persons: The Older Person's Exercise and Nutrition Study. *J Am Med Dir Assoc* **2020**, *21*:1229-1237.

30. Hamarsland H, Johansen MK, Seeberg F, Brochmann M, Garthe I, Benestad HB, Raastad T: Native Whey Induces Similar Adaptation to Strength Training as Milk, despite Higher Levels of Leucine, in Elderly Individuals. *Nutrients* **2019**, *11*.
31. Haß U, Kochlik B, Herpich C, Rudloff S, Norman K: Effects of an Omega-3 Supplemented, High-Protein Diet in Combination with Vibration and Resistance Exercise on Muscle Power and Inflammation in Old Adults: A Pilot Randomized Controlled Trial. *Nutrients* **2022**, *14*.
32. Haub MD, Wells AM, Tarnopolsky MA, Campbell WW: Effect of protein source on resistive-training-induced changes in body composition and muscle size in older men. *Am J Clin Nutr* **2002**, *76***:**511-517.
33. Haub MD, Wells AM, Campbell WW: Beef and soy-based food supplements differentially affect serum lipoprotein-lipid profiles because of changes in carbohydrate intake and novel nutrient intake ratios in older men who resistive-train. *Metabolism* **2005**, *54***:**769-774.
34. He LN, Li DM: Observation on the Clinical Effect of Dietary Nutrition Intervention Combined with Anti-resistance Training in Elderly Male Patients with Sarcopenia. *Smart Healthcare* **2022**, *8***:**44-47.
35. Holm L, Olesen JL, Matsumoto K, Doi T, Mizuno M, Alsted TJ, Mackey AL, Schwarz P, Kjaer M: Protein-containing nutrient supplementation following strength training enhances the effect on muscle mass, strength, and bone formation in postmenopausal women. *J Appl Physiol (1985)* **2008**, *105***:**274-281.
36. Holwerda AM, Overkamp M, Paulussen KJM, Smeets JSJ, van Kranenburg J, Backx EMP, Gijsen AP, Goessens JPB, Verdijk LB, van Loon LJC: Protein Supplementation after Exercise and before Sleep Does Not Further Augment Muscle Mass and Strength Gains during Resistance Exercise Training in Active Older Men. *J Nutr* **2018**, *148***:**1723-1732.
37. Kang Y, Kim N, Choi YJ, Lee Y, Yun J, Park SJ, Park HS, Chung YS, Park YK: Leucine-Enriched Protein Supplementation Increases Lean Body Mass in Healthy Korean Adults Aged 50 Years and Older: A Randomized, Double-Blind, Placebo-Controlled Trial. *Nutrients* **2020**, *12*.
38. Karelis AD, Messier V, Suppere C, Briand P, Rabasa-Lhoret R: Effect of cysteine-rich whey protein (immunocal(R)) supplementation in combination with resistance training on muscle strength and lean body mass in non-frail elderly subjects: a randomized, double-blind controlled study. *J Nutr Health Aging* **2015**, *19***:**531-536.
39. Kemmler W, Kohl M, Fröhlich M, Jakob F, Engelke K, von Stengel S, Schoene D: Effects of High-Intensity Resistance Training on Osteopenia and Sarcopenia Parameters in Older Men with Osteosarcopenia-One-Year Results of the Randomized Controlled Franconian Osteopenia and Sarcopenia Trial (FrOST). *J Bone Miner Res* **2020**, *35***:**1634-1644.
40. Kemmler W, Kohl M, Jakob F, Engelke K, von Stengel S: Effects of High Intensity Dynamic Resistance Exercise and Whey Protein Supplements on Osteosarcopenia in Older Men with Low Bone and Muscle Mass. Final Results of the Randomized Controlled FrOST Study. *Nutrients* **2020**, *12*.
41. Krause M, Crognale D, Cogan KE, Contarelli S, Egan B, Newsholme P, De Vito G: The effects of a combined bodyweight-based and elastic bands resistance training, with or without protein supplementation, on muscle mass, signaling and heat shock response in healthy older people. *Exp Gerontol* **2019**, *115***:**104-113.
42. Kukuljan S, Nowson CA, Bass SL, Sanders K, Nicholson GC, Seibel MJ, Salmon J, Daly RM: Effects of a multi-component exercise program and calcium-vitamin-D3-fortified milk on bone

mineral density in older men: a randomised controlled trial. *Osteoporos Int* **2009**, *20*:1241-1251.

43. Kukuljan S, Nowson CA, Sanders K, Daly RM: Effects of resistance exercise and fortified milk on skeletal muscle mass, muscle size, and functional performance in middle-aged and older men: an 18-mo randomized controlled trial. *J Appl Physiol (1985)* **2009**, *107*:1864-1873.
44. Kwon J, Yoshida Y, Yoshida H, Kim H, Suzuki T, Lee Y: Effects of a combined physical training and nutrition intervention on physical performance and health-related quality of life in prefrail older women living in the community: a randomized controlled trial. *J Am Med Dir Assoc* **2015**, *16*:263.e261-268.
45. Lamb DA, Moore JH, Smith MA, Vann CG, Osburn SC, Ruple BA, Fox CD, Smith KS, Altonji OM, Power ZM, et al: The effects of resistance training with or without peanut protein supplementation on skeletal muscle and strength adaptations in older individuals. *J Int Soc Sports Nutr* **2020**, *17*:66.
46. Leenders M, Verdijk LB, Van der Hoeven L, Van Kranenburg J, Nilwik R, Wodzig WK, Senden JM, Keizer HA, Van Loon LJ: Protein supplementation during resistance-type exercise training in the elderly. *Med Sci Sports Exerc* **2013**, *45*:542-552.
47. Liao CD, Liao YH, Liou TH, Hsieh CY, Kuo YC, Chen HC: Effects of Protein-Rich Nutritional Composition Supplementation on Sarcopenia Indices and Physical Activity during Resistance Exercise Training in Older Women with Knee Osteoarthritis. *Nutrients* **2021**, *13*:2487.
48. Maesta N, Nahas EA, Nahas-Neto J, Orsatti FL, Fernandes CE, Traiman P, Burini RC: Effects of soy protein and resistance exercise on body composition and blood lipids in postmenopausal women. *Maturitas* **2007**, *56*:350-358.
49. McKenna CF, Salvador AF, Hughes RL, Scaroni SE, Alamilla RA, Askow AT, Paluska SA, Dilger AC, Holscher HD, De Lisio M: Higher protein intake during resistance training does not potentiate strength, but modulates gut microbiota, in middle-aged adults: a randomized control trial. *Am J Physiology-Endocrinology and Metabolism* **2021**, *320*:E900-E913.
50. Mertz KH, Reitelseder S, Bechshoeft R, Bulow J, Højfeldt G, Jensen M, Schacht SR, Lind MV, Rasmussen MA, Mikkelsen UR, et al: The effect of daily protein supplementation, with or without resistance training for 1 year, on muscle size, strength, and function in healthy older adults: A randomized controlled trial. *Am J Clin Nutr* **2021**, *113*:790-800.
51. Miller EG, Nowson CA, Dunstan DW, Kerr DA, Menzies D, Daly RM: Effects of whey protein plus vitamin D supplementation combined with progressive resistance training on glycaemic control, body composition, muscle function and cardiometabolic risk factors in middle-aged and older overweight/obese adults with type 2 diabetes: A 24-week randomized controlled trial. *Diabetes Obes Metab* **2021**, *23*:938-949.
52. Mitchell CJ, D'Souza RF, Mitchell SM, Figueiredo VC, Miller BF, Hamilton KL, Peelor FF, 3rd, Coronet M, Pileggi CA, Durainayagam B, et al: Impact of dairy protein during limb immobilization and recovery on muscle size and protein synthesis; a randomized controlled trial. *J Appl Physiol (1985)* **2018**, *124*:717-728.
53. Molnar A, Jonasne Sztruhar I, Csontos AA, Ferencz C, Varbiro S, Szekacs B: Special nutrition intervention is required for muscle protective efficacy of physical exercise in elderly people at highest risk of sarcopenia. *Physiol Int* **2016**, *103*:368-376.
54. Mori H, Tokuda Y: Effect of whey protein supplementation after resistance exercise on the muscle mass and physical function of healthy older women: A randomized controlled trial.

Geriatr Gerontol Int **2018**, *18*:1398-1404.

55. Mori H, Tokuda Y: De-Training Effects Following Leucine-Enriched Whey Protein Supplementation and Resistance Training in Older Adults with Sarcopenia: A Randomized Controlled Trial with 24 Weeks of Follow-Up. *J Nutr Health Aging* **2022**, *26*:994-1002.
56. Nabuco HCG, Tomeleri CM, Sugihara Junior P, Fernandes RR, Cavalcante EF, Antunes M, Ribeiro AS, Teixeira DC, Silva AM, Sardinha LB, Cyrino ES: Effects of Whey Protein Supplementation Pre- or Post-Resistance Training on Muscle Mass, Muscular Strength, and Functional Capacity in Pre-Conditioned Older Women: A Randomized Clinical Trial. *Nutrients* **2018**, *10*.
57. Nabuco HCG, Tomeleri CM, Sugihara Junior P, Fernandes RR, Cavalcante EF, Venturini D, Barbosa DS, Silva AM, Sardinha LB, Cyrino ES: Effects of pre- or post-exercise whey protein supplementation on body fat and metabolic and inflammatory profile in pre-conditioned older women: A randomized, double-blind, placebo-controlled trial. *Nutr Metab Cardiovasc Dis* **2019**, *29*:290-300.
58. Nabuco HCG, Tomeleri CM, Fernandes RR, Sugihara Junior P, Cavalcante EF, Cunha PM, Antunes M, Nunes JP, Venturini D, Barbosa DS, et al: Effect of whey protein supplementation combined with resistance training on body composition, muscular strength, functional capacity, and plasma-metabolism biomarkers in older women with sarcopenic obesity: A randomized, double-blind, placebo-controlled trial. *Clin Nutr ESPEN* **2019**, *32*:88-95.
59. Nabuco HCG, Tomeleri CM, Fernandes RR, Sugihara Junior P, Cavalcante EF, Venturini D, Barbosa DS, Silva AM, Sardinha LB, Cyrino ES: Effects of Protein Intake Beyond Habitual Intakes Associated With Resistance Training on Metabolic Syndrome-Related Parameters, Isokinetic Strength, and Body Composition in Older Women. *J Aging Phys Act* **2019**, *27*:545–552.
60. Nilsson MI, Mikhail A, Lan L, Di Carlo A, Hamilton B, Barnard K, Hettinga BP, Hatcher E, Tarnopolsky MG, Nederveen JP, et al: A Five-Ingredient Nutritional Supplement and Home-Based Resistance Exercise Improve Lean Mass and Strength in Free-Living Elderly. *Nutrients* **2020**, *12*.
61. Oesen S, Halper B, Hofmann M, Jandrasits W, Franzke B, Strasser EM, Graf A, Tschan H, Bachl N, Quittan M, et al: Effects of elastic band resistance training and nutritional supplementation on physical performance of institutionalised elderly - A randomized controlled trial. *Exp Gerontol* **2015**, *72*:99-108.
62. Orsatti FL, Maestá N, de Oliveira EP, Nahas Neto J, Burini RC, Nunes PRP, Souza AP, Martins FM, Nahas EP: Adding Soy Protein to Milk Enhances the Effect of Resistance Training on Muscle Strength in Postmenopausal Women. *J Diet Suppl* **2018**, *15*:140-152.
63. Roschel H, Hayashi AP, Fernandes AL, Jambassi-Filho JC, Hevia-Larraín V, de Capitani M, Santana DA, Gonçalves LS, de Sá-Pinto AL, Lima FR, et al: Supplement-based nutritional strategies to tackle frailty: A multifactorial, double-blind, randomized placebo-controlled trial. *Clin Nutr* **2021**, *40*:4849-4858.
64. Seino S, Sumi K, Narita M, Yokoyama Y, Ashida K, Kitamura A, Shinkai S: Effects of Low-Dose Dairy Protein Plus Micronutrient Supplementation during Resistance Exercise on Muscle Mass and Physical Performance in Older Adults: A Randomized, Controlled Trial. *J Nutr Health Aging* **2018**, *22*:59-67.
65. Shahar S, Kamaruddin NS, Badrasawi M, Sakian NI, Abd Manaf Z, Yassin Z, Joseph L: Effectiveness of exercise and protein supplementation intervention on body composition, functional fitness, and oxidative stress among elderly Malays with sarcopenia. *Clin Interv Aging* **2013**, *8*:1365-1375.

66. Shenoy S, Bedi R, Sandhu JS: Effect of soy isolate protein and resistance exercises on muscle performance and bone health of osteopenic/osteoporotic post-menopausal women. *J Women Aging* **2013**, *25*:183-198.

67. Soares ALS, Machado-Lima A, Brech GC, Greve JMD, Dos Santos JR, Inojossa TR, Rogero MM, Salles JEN, Santarem-Sobrinho JM, Davis CL, Alonso AC: The Influence of Whey Protein on Muscle Strength, Glycemic Control and Functional Tasks in Older Adults with Type 2 Diabetes Mellitus in a Resistance Exercise Program: Randomized and Triple Blind Clinical Trial. *Int J Environ Res Public Health* **2023**, *20*.

68. Sugihara Junior P, Ribeiro AS, Nabuco HCG, Fernandes RR, Tomeleri CM, Cunha PM, Venturini D, Barbosa DS, Schoenfeld BJ, Cyrino ES: Effects of Whey Protein Supplementation Associated With Resistance Training on Muscular Strength, Hypertrophy, and Muscle Quality in Preconditioned Older Women. *Int J Sport Nutr Exerc Metab* **2018**, *28*:528-535.

69. Tang W, Yan X, Fan P, Xu D, Li Y: Analysis of the Clincal Effect ofDietary Nutrition Intervention Combined with Resistance Training on the Cognitive Function of Elderly Male Patients with Sarcopenia. *Progr in Mod Biomed* **2020**, *20*:2698-2701.

70. Thomson RL, Brinkworth GD, Noakes M, Buckley JD: Muscle strength gains during resistance exercise training are attenuated with soy compared with dairy or usual protein intake in older adults: A randomized controlled trial. *Clin Nutr* **2016**, *35*:27-33.

71. Tieland M, Dirks ML, van der Zwaluw N, Verdijk LB, van de Rest O, de Groot LC, van Loon LJ: Protein supplementation increases muscle mass gain during prolonged resistance-type exercise training in frail elderly people: a randomized, double-blind, placebo-controlled trial. *J Am Med Dir Assoc* **2012**, *13*:713-719.

72. Dirks ML, Tieland M, Verdijk LB, Losen M, Nilwik R, Mensink M, de Groot LCPGM, van Loon LJC: Protein Supplementation Augments Muscle Fiber Hypertrophy but Does Not Modulate Satellite Cell Content During Prolonged Resistance-Type Exercise Training in Frail Elderly. *J Am Med Dir Assoc* **2017**, *18*:608-615.

73. Trevisan MC, Souza JM, Marucci Mde F: Influence of soy protein intake and weight training on the resting energy expenditure of postmenopausal women. *Rev Assoc Med Bras (1992)* **2010**, *56*:572-578.

74. Unterberger S, Aschauer R, Zöhrer PA, Draxler A, Franzke B, Strasser EM, Wagner KH, Wessner B: Effects of an increased habitual dietary protein intake followed by resistance training on fitness, muscle quality and body composition of seniors: A randomised controlled trial. *Clin Nutr* **2022**, *41*:1034-1045.

75. Verdijk LB, Jonkers RAM, Gleeson BG, Beelen M, Meijer K, Savelberg HHCM, Wodzig KWHW, Dendale P, Van Loon LJC: Protein supplementation before and after exercise does not further augment skeletal muscle hypertrophy after resistance training in elderly men. *American Journal of Clinical Nutrition* **2009**, *89*:608-616.

76. Verreijen AM, Verlaan S, Engberink MF, Swinkels S, de Vogel-van den Bosch J, Weijs PJ: A high whey protein-, leucine-, and vitamin D-enriched supplement preserves muscle mass during intentional weight loss in obese older adults: A double-blind randomized controlled trial. *Am J Clin Nutr* **2015**, *101*:279-286.

77. Vijayakumaran RK, Daly RM, Tan VPS: " We Want More"-Perspectives of Sarcopenic Older Women on the Feasibility of High-Intensity Progressive Resistance Exercises and a Whey-Protein Nutrition Intervention. *Front Nutr* **2023**, *10*:1176523.

78. Vikberg S, Sörlén N, Brandén L, Johansson J, Nordström A, Hult A, Nordström P: Effects of Resistance Training on Functional Strength and Muscle Mass in 70-Year-Old Individuals With Pre-sarcopenia: A Randomized Controlled Trial. *J Am Med Dir Assoc* **2019**, *20***:**28-34.
79. Villanueva MG, He J, Schroeder ET: Periodized resistance training with and without supplementation improve body composition and performance in older men. *Eur J Appl Physiol* **2014**, *114***:**891-905.
80. Weisgarber KD, Candow DG, Farthing JP: Whey protein and high-volume resistance training in postmenopausal women. *J Nutr Health Aging* **2015**, *19***:**511-517.
81. Yamada M, Kimura Y, Ishiyama D, Nishio N, Otobe Y, Tanaka T, Ohji S, Koyama S, Sato A, Suzuki M, et al: Synergistic effect of bodyweight resistance exercise and protein supplementation on skeletal muscle in sarcopenic or dynapenic older adults. *Geriatr Gerontol Int* **2019**.
82. Zdzieblik D, Jendricke P, Oesser S, Gollhofer A, König D: The Influence of Specific Bioactive Collagen Peptides on Body Composition and Muscle Strength in Middle-Aged, Untrained Men: A Randomized Controlled Trial. *Int J Environ Res Public Health* **2021**, *18*.
83. Zhao T, Gao HL, Guo NZ, Chea ZM, Wu XY, He DL, Wei LQ, Shen XC, Lv WY: Effect of health education and whey protein supplementation combined with resistance exercise on the intervention of middle — aged and elderly people with sarcopenia. *J Clin Exp Med* **2022**, *12***:**90-94.
84. Zhu LY, Chan R, Kwok T, Cheng KC, Ha A, Woo J: Effects of exercise and nutrition supplementation in community-dwelling older Chinese people with sarcopenia: a randomized controlled trial. *Age Ageing* **2019**, *48***:**220-228.
85. Maltais ML, Ladouceur JP, Dionne IJ: The Effect of Resistance Training and Different Sources of Postexercise Protein Supplementation on Muscle Mass and Physical Capacity in Sarcopenic Elderly Men. *J Strength Cond Res* **2016**, *30***:**1680-1687.

Table S12: Judgments in each domain of GRADE ranking system for muscle mass outcome

Treatment (Common comparator: RC)	Involved studies (n)	Participants (n)	Study limitation: Contribution of risks of bias of direct estimates to network estimates			Study limitation	Inconsistency (incoherence): Treatment effect, SMD (95%CI)			Inconsistency (incoherence)	Indirection (transitivity)					Imprecision		Publication bias		Certainty of evidence[a]
			High	Mod	Low	Judgment	Direct estimate	Indirect estimate	Network estimate	Judgment	P	I	C	O	Judgment	Network estimate	Judgment	Begg–Mazumdar rank correlation test	Judgment	
Casein+RT	2	46	46.17%	53.40%	0.44%	Serious limitations		0.87 (-0.13, 1.87)	0.87 (-0.13, 1.87)	No serious	Older people	PS+RT	RC, PLA, active comparator	LBM	No serious	0.87 (-0.13, 1.87)	Serious	*p < 0.01*	Serious	⊕⊖⊖⊖
Meat+RT	3	131	46.45%	53.29%	0.27%	Serious limitations		1.23 (0.41, 2.04)	1.23 (0.41, 2.04)	No serious	Older people	PS+RT	RC, PLA, active comparator	LBM	No serious	1.23 (0.41, 2.04)	No serious	*p < 0.01*	Serious	⊕⊕⊖⊖
MP+RT	16	490	27.61%	72.33%	0.05%	Serious limitations	1.31 (0.41, 2.22)	1.22 (0.77, 1.68)	1.24 (0.83, 1.65)	No serious	Older people	PS+RT	RC, PLA, active comparator	LBM	No serious	1.24 (0.83, 1.65)	No serious	*p < 0.01*	Serious	⊕⊕⊖⊖
Peanut+RT	1	20	45.08%	54.78%	0.14%	Serious limitations		0.95 (-0.14, 2.05)	0.95 (-0.14, 2.05)	No serious	Older people	PS+RT	RC, PLA, active comparator	LBM	No serious	0.95 (-0.14, 2.05)	Serious	*p < 0.01*	Serious	⊕⊖⊖⊖
SP+RT	11	207	9.26%	88.56%	2.20%	Serious limitations	1.06 (0.49, 1.63)	1.05 (0.40, 1.70)	1.06 (0.63, 1.49)	No serious	Older people	PS+RT	RC, PLA, active comparator	LBM	No serious	1.06 (0.63, 1.49)	No serious	*p < 0.01*	Serious	⊕⊕⊖⊖
WP+RT	40	991	7.15%	91.88%	0.97%	Serious limitations	1.36 (0.90, 1.83)	1.21 (0.74, 1.68)	1.29 (0.96, 1.62)	No serious	Older people	PS+RT	RC, PLA, active comparator	LBM	No serious	1.29 (0.96, 1.62)	No serious	*p < 0.01*	Serious	⊕⊕⊖⊖
Meat	1	10	43.18%	56.49%	0.32%	Serious limitations		-0.38 (-1.95, 1.19)	-0.38 (-1.95, 1.19)	No serious	Older people	PS	RC, PLA, active comparator	LBM	No serious	-0.38 (-1.95, 1.19)	Serious	*p < 0.01*	Serious	⊕⊖⊖⊖
MP	4	99	32.00%	67.88%	0.12%	Serious limitations	0.31 (-0.62, 1.24)	0.58 (-0.30, 1.74)	0.45 (-0.19, 1.09)	No serious	Older people	PS	RC, PLA, active comparator	LBM	No serious	0.45 (-0.19, 1.09)	Serious	*p < 0.01*	Serious	⊕⊖⊖⊖
SP	6	90	9.69%	89.89%	0.42%	Serious limitations	0.19 (-0.49, 0.87)	-0.17 (-1.11, 0.78)	0.07 (-0.48, 0.62)	No serious	Older people	PS	RC, PLA, active comparator	LBM	No serious	0.07 (-0.48, 0.62)	Serious	*p < 0.01*	Serious	⊕⊖⊖⊖
WP	10	203	12.29%	87.39%	0.35%	Serious limitations	0.31 (-0.18, 0.81)	0.46 (-0.33, 1.26)	0.36 (-0.07, 0.78)	No serious	Older people	PS	RC, PLA, active comparator	LBM	No serious	0.36 (-0.07, 0.78)	Serious	*p < 0.01*	Serious	⊕⊖⊖⊖
RT	56	1399	9.64%	90.20%	0.18%	Serious limitations	0.79 (0.42, 1.15)	0.51 (-0.06, 1.09)	0.71 (0.40, 1.02)	No serious	Older people	RT	RC, PLA, active comparator	LBM	No serious	0.71 (0.40, 1.02)	No serious	*p < 0.01*	Serious	⊕⊕⊖⊖

[a]Certainty of evidence is graded as follows: High: ⊕⊕⊕⊕; Moderate: ⊕⊕⊕⊖; Low: ⊕⊕⊖⊖; Very low: ⊕⊖⊖⊖.

CI, confidence interval; GRADE, Grading of Recommendations, Assessment, Development and Evaluations; LBM, lean body mass; MP, milk protein; PLA, placebo supplementation; PS, protein supplementation; RT, resistance training; SMD, standardized mean difference; SP, soy protein; WP, whey protein RC, regular care

Table S13: Judgments in each domain of GRADE framework for handgrip strength outcome

Treatment (Common comparator: RC)	Involved studies (n)	Participants (n)	Study limitation				Inconsistency (incoherence)				Indirection (transitivity)					Imprecision		Publication bias		Certainty of evidence[a]
			Contribution of risks of bias of direct estimates to network estimates			Rate	Treatment effect, SMD (95%CI)			Rate	P	I	C	O	Rate	Network estimate	Rate	Begg–Mazumdar rank correlation test	Rate	
			High	Mod	Low		Direct estimate	Indirect estimate	Network estimate											
Casein+RT	2	48	86.51%	10.82%	2.66%	Serious limitations	2.98 (0.99, 4.97)	-0.13 (-1.90, 1.64)	1.24 (-0.08, 2.56)	Serious	Older people	PS+RT	RC, PLA, active comparator	HG	No serious	1.24 (-0.08, 2.56)	Serious	*p = 0.14*	Undetected	⊕⊖⊖⊖
Meat+RT	1	21	11.00%	86.44%	2.58%	Serious limitations	-0.88 (-2.82, 1.05)	-0.80 (-4.45, 2.85)	-0.87 (-2.57, 0.84)	No serious	Older people	PS+RT	RC, PLA, active comparator	HG	No serious	-0.87 (-2.57, 0.84)	Serious	*p = 0.14*	Undetected	⊕⊕⊖⊖
MP+RT	9	368	34.14%	60.42%	5.43%	Serious limitations	0.22 (-1.68, 2.13)	1.48 (0.64, 2.32)	1.27 (0.50, 2.04)	No serious	Older people	PS+RT	RC, PLA, active comparator	HG	No serious	1.27 (0.50, 2.04)	No serious	*p = 0.14*	Undetected	⊕⊕⊕⊖
SP+RT	4	99	20.46%	24.56%	54.99%	No serious limitations	1.51 (0.11, 2.90)	1.31 (0.60, 2.56)	1.40 (0.47, 2.33)	No serious	Older people	PS+RT	RC, PLA, active comparator	HG	No serious	1.40 (0.47, 2.33)	No serious	*p = 0.14*	Undetected	⊕⊕⊕⊕
WP+RT	19	596	65.33%	29.84%	4.82%	Serious limitations	1.53 (0.88, 2.19)	1.30 (0.34, 2.26)	1.46 (0.92, 2.00)	No serious	Older people	PS+RT	RC, PLA, active comparator	HG	No serious	1.46 (0.92, 2.00)	No serious	*p = 0.14*	Undetected	⊕⊕⊕⊖
SP	1	15	12.52%	16.60%	70.87%	No serious limitations	0.90 (-1.08, 2.88)	-1.17 (-4.24, 1.91)	0.29 (-1.37, 1.96)	No serious	Older people	PS	RC, PLA, active comparator	HG	No serious	0.29 (-1.37, 1.96)	Serious	*p = 0.14*	Undetected	⊕⊕⊕⊖
WP	6	121	83.35%	13.55%	3.11%	Serious limitations	0.12 (-0.77, 1.01)	1.19 (-0.09, 2.48)	0.47 (-0.26, 1.20)	No serious	Older people	PS	RC, PLA, active comparator	HG	No serious	0.47 (-0.26, 1.20)	Serious	*p = 0.14*	Undetected	⊕⊕⊖⊖
RT	25	737	33.37%	58.84%	7.77%	Serious limitations	0.56 (-0.13, 1.26)	1.65 (0.78, 2.52)	0.99 (0.44, 1.53)	No serious	Older people	RT	RC, PLA, active comparator	HG	No serious	0.99 (0.44, 1.53)	No serious	*p = 0.14*	Undetected	⊕⊕⊕⊖

[a]Certainty of evidence is graded as follows: High: ⊕⊕⊕⊕; Moderate: ⊕⊕⊕⊖; Low: ⊕⊕⊖⊖; Very low: ⊕⊖⊖⊖.

CI, confidence interval; GRADE, Grading of Recommendations, Assessment, Development and Evaluations; HG, handgrip strength; MP, milk protein; PLA, placebo supplementation; PS, protein supplementation; RT, resistance training; SMD, standardized mean difference; SP, soy protein; WP, whey protein RC, regular care

Table S14: Judgments in each domain of GRADE ranking system for leg strength outcome

Treatment (Common comparator: RC)	Involved studies (n)	Participants (n)	Study limitation: Contribution of risks of bias of direct estimates to network estimates			Study limitation	Inconsistency (incoherence): Treatment effect SMD (95%CI)			Inconsistency (incoherence)	Indirection (transitivity)					Imprecision		Publication bias		Certainty of evidence[a]
			High	Mod	Low	Judgment	Direct estimate	Indirect estimate	Network estimate	Judgment	P	I	C	O	Judgment	Network estimate	Judgment	Begg–Mazumdar rank correlation test	Judgment	
Casein+RT	1	33	39.88%	59.28%	0.86%	Serious limitations		1.08 (-0.17; 2.32)	1.08 (-0.17; 2.32)	No serious	Older people	PS+RT	RC, PLA, active comparator	Leg strength	No serious	1.08 (-0.17; 2.32)	Serious	*p* < 0.01	Strongly suspected	⊕⊖⊖⊖
Meat+RT	3	130	45.89%	53.83%	0.26%	Serious limitations		1.27 (0.49; 2.05)	1.27 (0.49; 2.05)	No serious	Older people	PS+RT	RC, PLA, active comparator	Leg strength	No serious	1.27 (0.49; 2.05)	No serious	*p* < 0.01	Strongly suspected	⊕⊕⊖⊖
MP+RT	11	309	21.89%	77.91%	0.20%	Serious limitations	0.69 (-0.47; 1.85)	1.45 (0.94, 1.97)	1.33 (0.86; 1.80)	No serious	Older people	PS+RT	RC, PLA, active comparator	Leg strength	No serious	1.33 (0.86; 1.80)	No serious	*p* < 0.01	Strongly suspected	⊕⊕⊖⊖
SP+RT	10	204	6.60%	91.12%	2.27%	Serious limitations	1.73 (1.14; 2.32)	0.80 (0.13, 1.47)	1.32 (0.88; 1.77)	Serious	Older people	PS+RT	RC, PLA, active comparator	Leg strength	No serious	1.32 (0.88; 1.77)	No serious	*p* < 0.01	Strongly suspected	⊕⊖⊖⊖
WP+RT	29	754	6.39%	92.35%	1.27%	Serious limitations	1.30 (0.73; 1.87)	1.50 (0.99, 2.01)	1.41 (1.03; 1.79)	No serious	Older people	PS+RT	RC, PLA, active comparator	Leg strength	No serious	1.41 (1.03; 1.79)	No serious	*p* < 0.01	Strongly suspected	⊕⊕⊖⊖
Meat	1	10	40.29%	59.35%	0.38%	Serious limitations		-0.35 (-1.88; 1.18)	-0.35 (-1.88; 1.18)	No serious	Older people	PS	RC, PLA, active comparator	Leg strength	No serious	-0.35 (-1.88; 1.18)	Serious	*p* < 0.01	Strongly suspected	⊕⊖⊖⊖
MP	3	88	23.23%	76.68%	0.09%	Serious limitations	-0.05 (-0.91; 0.82)	0.94 (-0.22, 2.09)	0.31 (-0.38; 1.00)	No serious	Older people	PS	RC, PLA, active comparator	Leg strength	No serious	0.31 (-0.38; 1.00)	Serious	*p* < 0.01	Strongly suspected	⊕⊖⊖⊖
SP	4	70	2.72%	96.81%	0.50%	Serious limitations	0.41 (-0.32; 1.14)	-1.31 (-2.68, 0.07)	0.03 (-0.61; 0.68)	Serious	Older people	PS	RC, PLA, active comparator	Leg strength	No serious	0.03 (-0.61; 0.68)	Serious	*p* < 0.01	Strongly suspected	⊕⊖⊖⊖
WP	5	127	12.93%	86.61%	0.46%	Serious limitations	0.20 (-0.46; 0.85)	0.07 (-0.80, 0.94)	0.15 (-0.37; 0.67)	No serious	Older people	PS	RC, PLA, active comparator	Leg strength	No serious	0.15 (-0.37; 0.67)	Serious	*p* < 0.01	Strongly suspected	⊕⊖⊖⊖
RT	40	1040	7.66%	92.06%	0.25%	Serious limitations	0.94 (0.53; 1.36)	1.06 (0.41, 1.70)	0.98 (0.63; 1.33)	No serious	Older people	RT	RC, PLA, active comparator	Leg strength	No serious	0.98 (0.63; 1.33)	No serious	*p* < 0.01	Strongly suspected	⊕⊕⊖⊖

[a]Certainty of evidence is graded as follows: High: ⊕⊕⊕⊕; Moderate: ⊕⊕⊕⊖; Low: ⊕⊕⊖⊖; Very low: ⊕⊖⊖⊖.

CI, confidence interval; GRADE, Grading of Recommendations, Assessment, Development and Evaluations; LBM, lean body mass; MP, milk protein; PLA, placebo supplementation; PS, protein supplementation; RT, resistance training; SMD, standardized mean difference; SP, soy protein; WP, whey protein RC, regular care

Table S15: Judgments in each domain of GRADE ranking system for walking speed outcome

Treatment (Common comparator: RC)	Involved studies (n)	Participants (n)	Study limitation				Inconsistency (incoherence)				Indirection (transitivity)					Imprecision		Publication bias		Certainty of evidence[a]
			Contribution of risks of bias of direct estimates to network estimates			Judgment	Treatment effect SMD (95%CI)			Judgment	P	I	C	O	Judgment	Network estimate	Judgment	Begg–Mazumdar rank correlation test	Judgment	
			High	Mod	Low		Direct estimate	Indirect estimate	Network estimate											
Meat+RT	2	101	41.24%	58.76%	0.00%	Serious limitations	0.04 (-1.15, 1.22)	0.97 (-0.08, 2.02)	0.56 (-0.23, 1.35)	No serious	Older people	PS+RT	RC, PLA, active comparator	Walking speed	No serious	0.56 (-0.23, 1.35)	Serious	*p = 0.14*	Undetected	⊕⊕⊖⊖
MP+RT	10	350	31.47%	68.53%	0.00%	Serious limitations	0.19 (-0.94, 1.32)	0.60 (0.09, 1.11)	0.53 (0.07, 1.00)	No serious	Older people	PS+RT	RC, PLA, active comparator	Walking speed	No serious	0.53 (0.07, 1.00)	No serious	*p = 0.14*	Undetected	⊕⊕⊕⊖
SP+RT	3	87	18.87%	81.13%	0.00%	Serious limitations	0.55 (-0.28, 1.37)	0.65 (-0.30, 1.60)	0.59 (-0.03, 1.21)	No serious	Older people	PS+RT	RC, PLA, active comparator	Walking speed	No serious	0.59 (-0.03, 1.21)	Serious	*p = 0.14*	Undetected	⊕⊕⊖⊖
WP+RT	21	601	25.39%	74.60%	0.00%	Serious limitations	0.84 (0.43, 1.25)	0.50 (-0.11, 1.10)	0.73 (0.39, 1.07)	No serious	Older people	PS+RT	RC, PLA, active comparator	Walking speed	No serious	0.73 (0.39, 1.07)	No serious	*p = 0.14*	Undetected	⊕⊕⊕⊖
MP	2	70	64.36%	35.66%	0.00%	Serious limitations	-0.28 (-1.41, 0.85)	-0.16 (-1.28, 0.96)	-0.22 (-1.02, 0.57)	No serious	Older people	PS	RC, PLA, active comparator	Walking speed	No serious	-0.22 (-1.02, 0.57)	Serious	*p = 0.14*	Undetected	⊕⊕⊖⊖
SP	3	24	70.25%	29.77%	0.00%	Serious limitations	0.47 (-0.72, 1.66)	0.08 (-1.82, 1.97)	0.36 (-0.65, 1.37)	No serious	Older people	PS	RC, PLA, active comparator	Walking speed	No serious	0.36 (-0.65, 1.37)	Serious	*p = 0.14*	Undetected	⊕⊕⊖⊖
WP	9	189	17.46%	82.55%	0.00%	Serious limitations	0.76 (0.30, 1.22)	-0.21 (-1.05, 0.62)	0.53 (0.13, 0.94)	Serious	Older people	PS	RC, PLA, active comparator	Walking speed	No serious	0.53 (0.13, 0.94)	No serious	*p = 0.14*	Undetected	⊕⊕⊖⊖
RT	29	872	26.45%	73.56%	0.00%	Serious limitations	0.59 (0.22, 0.95)	0.28 (-0.39, 0.95)	0.52 (0.19, 0.84)	No serious	Older people	RT	RC, PLA, active comparator	Walking speed	No serious	0.52 (0.19, 0.84)	No serious	*p = 0.14*	Undetected	⊕⊕⊕⊖

[a]Certainty of evidence is graded as follows: High: ⊕⊕⊕⊕; Moderate: ⊕⊕⊕⊖; Low: ⊕⊕⊖⊖; Very low: ⊕⊖⊖⊖.
CI, confidence interval; GRADE, Grading of Recommendations, Assessment, Development and Evaluations; LBM, lean body mass; MP, milk protein; PLA, placebo supplementation; PS, protein supplementation; RT, resistance training; SMD, standardized mean difference; SP, soy protein; WP, whey protein RC, regular care

Table S16: Judgments in each domain of GRADE ranking system for chair rise outcome

Treatment (Common comparator: RC)	Involved studies (n)	Participants (n)	Study limitation				Inconsistency (incoherence)				Indirection (transitivity)					Imprecision		Publication bias		Certainty of evidence[a]
			Contribution of risks of bias of direct estimates to network estimates			Judgment	Treatment effect SMD (95%CI)			Judgment	P	I	C	O	Judgment	Network estimate	Judgment	Begg–Mazumdar rank correlation test	Judgment	
			High	Mod	Low		Direct estimate	Indirect estimate	Network estimate											
Meat+RT	1	43	20.68%	74.68%	4.66%	Serious limitations		0.34 (-0.29, 0.97)	0.34 (-0.29, 0.97)	No serious	Older people	PS+RT	RC, PLA, active comparator	Chair rise	No serious	0.34 (-0.29, 0.97)	Serious	*p* = 0.72	Undetected	⊕⊕⊖⊖
MP+RT	10	307	39.35%	56.54%	4.12%	Serious limitations	0.40 (-0.23, 1.03)	0.68 (0.33, 1.04)	0.62 (0.31, 0.92)	No serious	Older people	PS+RT	RC, PLA, active comparator	Chair rise	No serious	0.62 (0.31, 0.92)	No serious	*p* = 0.72	Undetected	⊕⊕⊕⊖
SP+RT	2	58	18.69%	19.10%	62.21%	No serious limitations	0.92 (0.31, 1.54)	0.82 (0.20, 1.45)	0.87 (0.44, 1.31)	No serious	Older people	PS+RT	RC, PLA, active comparator	Chair rise	No serious	0.87 (0.44, 1.31)	No serious	*p* = 0.72	Undetected	⊕⊕⊕⊕
WP+RT	16	488	49.42%	44.70%	5.88%	Serious limitations	0.98 (0.64, 1.32)	0.61 (0.20, 1.03)	0.84 (0.57, 1.10)	No serious	Older people	PS+RT	RC, PLA, active comparator	Chair rise	No serious	0.84 (0.57, 1.10)	No serious	*p* = 0.72	Undetected	⊕⊕⊕⊖
MP	2	25	57.59%	39.29%	3.10%	Serious limitations		0.31 (-0.44, 1.05)	0.31 (-0.44, 1.05)	No serious	Older people	PS	RC, PLA, active comparator	Chair rise	No serious	0.31 (-0.44, 1.05)	Serious	*p* = 0.72	Undetected	⊕⊕⊖⊖
WP	4	70	81.51%	15.63%	2.86%	Serious limitations	0.02 (-0.36, 0.41)	0.75 (-0.13, 1.63)	0.14 (-0.21, 0.49)	No serious	Older people	PS	RC, PLA, active comparator	Chair rise	No serious	0.14 (-0.21, 0.49)	Serious	*p* = 0.72	Undetected	⊕⊕⊖⊖
RT	24	737	30.55%	62.56%	6.89%	Serious limitations	0.82 (0.53, 1.11)	0.35 (-0.12, 0.83)	0.69 (0.44, 0.94)	No serious	Older people	RT	RC, PLA, active comparator	Chair rise	No serious	0.69 (0.44, 0.94)	No serious	*p* = 0.72	Undetected	⊕⊕⊕⊖

[a]Certainty of evidence is graded as follows: High: ⊕⊕⊕⊕; Moderate: ⊕⊕⊕⊖; Low: ⊕⊕⊖⊖; Very low: ⊕⊖⊖⊖.

CI, confidence interval; GRADE, Grading of Recommendations, Assessment, Development and Evaluations; LBM, lean body mass; MP, milk protein; PLA, placebo supplementation; PS, protein supplementation; RT, resistance training; SMD, standardized mean difference; SP, soy protein; WP, whey protein RC, regular care

Table S17: Judgments in each domain of GRADE ranking system for timed up-and-go outcome

Treatment (Common comparator: RC)	Involved studies (n)	Participants (n)	Study limitation: Contribution of risks of bias of direct estimates to network estimates				Inconsistency (incoherence): Treatment effect SMD (95%CI)				Indirection (transitivity)					Imprecision		Publication bias		Certainty of evidence[a]
			High	Mod	Low	Judgment	Direct estimate	Indirect estimate	Network estimate	Judgment	P	I	C	O	Judgment	Network estimate	Judgment	Begg–Mazumdar rank correlation test	Judgment	
Meat+RT	2	120	66.66%	33.33%	0.00%	Serious limitations		0.15 (-0.74, 1.03)	0.15 (-0.74, 1.03)	No serious	Older people	PS+RT	RC, PLA, active comparator	TUG	No serious	0.15 (-0.74, 1.03)	Serious	*p* = 0.17	Undetected	⊕⊕⊖⊖
MP+RT	2	47	100.00%	0.00%	0.00%	Serious limitations	0.33 (-0.16, 0.82)		0.33 (-0.16, 0.82)	No serious	Older people	PS+RT	RC, PLA, active comparator	TUG	No serious	0.33 (-0.16, 0.82)	Serious	*p* = 0.17	Undetected	⊕⊕⊖⊖
SP+RT	1	22	29.32%	41.35%	29.32%	Serious limitations		-0.30 (-1.30, 0.70)	-0.30 (-1.30, 0.70)	No serious	Older people	PS+RT	RC, PLA, active comparator	TUG	No serious	-0.30 (-1.30, 0.70)	Serious	*p* = 0.17	Undetected	⊕⊕⊖⊖
WP+RT	4	244	33.08%	65.39%	1.52%	Serious limitations		0.15 (-0.72, 1.02)	0.15 (-0.72, 1.02)	No serious	Older people	PS+RT	RC, PLA, active comparator	TUG	No serious	0.15 (-0.72, 1.02)	Serious	*p* = 0.17	Undetected	⊕⊕⊖⊖
RT	7	360	50.00%	50.00%	0.00%	Serious limitations		0.12 (-0.73, 0.97)	0.12 (-0.73, 0.97)	No serious	Older people	RT	RC, PLA, active comparator	TUG	No serious	0.12 (-0.73, 0.97)	Serious	*p* = 0.17	Undetected	⊕⊕⊖⊖

[a]Certainty of evidence is graded as follows: High: ⊕⊕⊕⊕; Moderate: ⊕⊕⊕⊖; Low: ⊕⊕⊖⊖; Very low: ⊕⊖⊖⊖.

CI, confidence interval; GRADE, Grading of Recommendations, Assessment, Development and Evaluations; LBM, lean body mass; MP, milk protein; PLA, placebo supplementation; PS, protein supplementation; RT, resistance training; SMD, standardized mean difference; SP, soy protein; TUG, timed up and go; WP, whey protein RC, regular care

Table S18: Judgments in each domain of GRADE ranking system for global mobility (SPPB) outcome

Treatment (Common comparator: RC)	Involved studies (n)	Participants (n)	Study limitation				Inconsistency (incoherence)				Indirection (transitivity)					Imprecision		Publication bias		Certainty of evidence[a]
			Contribution of risks of bias of direct estimates to network estimates			Judgment	Treatment effect SMD (95%CI)			Judgment	P	I	C	O	Judgment	Network estimate	Judgment	Begg–Mazumdar rank correlation test	Judgment	
			High	Mod	Low		Direct estimate	Indirect estimate	Network estimate											
Casein+RT	1	28	100.00%	0.00%	0.00%	Serious limitations	0.90 (-0.26, 2.06)		0.90 (-0.26, 2.06)	No serious	Older people	PS+RT	RC, PLA, active comparator	SPPB	No serious	0.90 (-0.26, 2.06)	Serious	*p* = 0.38	Undetected	⊕⊕⊖⊖
MP+RT	5	151	69.51%	30.49%	0.00%	Serious limitations	0.33 (-0.80, 1.46)	1.46 (0.47, 2.45)	0.97 (0.22, 1.71)	No serious	Older people	PS+RT	RC, PLA, active comparator	SPPB	No serious	0.97 (0.22, 1.71)	No serious	*p* = 0.38	Undetected	⊕⊕⊕⊖
WP+RT	7	222	83.29%	16.70%	0.00%	Serious limitations	1.79 (1.09, 2.48)	022 (-1.12, 1.56)	1.45 (0.84, 2.07)	Serious	Older people	PS+RT	RC, PLA, active comparator	SPPB	No serious	1.45 (0.84, 2.07)	No serious	*p* = 0.38	Undetected	⊕⊕⊖⊖
WP	3	15	26.44%	73.57%	0.00%	Serious limitations	0.63 (-0.64, 1.89)	2.94 (0.75, 5.14)	1.21 (0.11, 2.30)	No serious	Older people	PS	RC, PLA, active comparator	SPPB	No serious	1.21 (0.11, 2.30)	No serious	*p* = 0.38	Undetected	⊕⊕⊕⊖
RT	9	209	64.04%	35.95%	0.00%	Serious limitations	0.30 (-0.95, 1.55)	1.53 (0.72, 2.33)	1.17 (0.49, 1.84)	No serious	Older people	RT	RC, PLA, active comparator	SPPB	No serious	1.17 (0.49, 1.84)	No serious	*p* = 0.38	Undetected	⊕⊕⊕⊖

[a]Certainty of evidence is graded as follows: High: ⊕⊕⊕⊕; Moderate: ⊕⊕⊕⊖; Low: ⊕⊕⊖⊖; Very low: ⊕⊖⊖⊖.

CI, confidence interval; GRADE, Grading of Recommendations, Assessment, Development and Evaluations; LBM, lean body mass; MP, milk protein; PLA, placebo supplementation; PS, protein supplementation; RT, resistance training; SMD, standardized mean difference; SP, soy protein; SPPB, Short Physical Performance Battery; WP, whey protein RC, regular care
